# A clinically translatable, irreversibly attenuated* Salmonella* strain as a next-generation adjuvant for checkpoint immunotherapy

**DOI:** 10.7150/thno.129698

**Published:** 2026-05-29

**Authors:** Dinh-Huy Nguyen, Sung-Hwan You, Khuynh Van Nguyen, Phuong Thi-Minh Nguyen, Hien Thi-Thu Ngo, Khang Vuong Tran, Thanh Quang Tran, Miryoung Song, Yeongjin Hong, Jung-Joon Min

**Affiliations:** 1 Institute for Molecular Imaging and Theranostics, Chonnam National University, Hwasun, Jeonnam 61469, Republic of Korea.; 2 Department of Nuclear Medicine, Chonnam National University Medical School and Hwasun Hospital, Hwasun, Jeonnam 61469, Republic of Korea.; 3 Institute of Biomedical Science, Hwasun Chonnam National University Hospital, Gwangju 61469, Republic of Korea.; 4 CNCure Co., Ltd., Hwasun, Jeonnam 58128, Republic of Korea.; 5 Department of Biomedical Science (BrainKorea21 Plus), Chonnam National University Graduate School, Gwangju 61469, Republic of Korea.; 6 Department of Bioscience and Biotechnology, Hankuk University of Foreign Studies, Yongin, Republic of Korea.; 7 Department of Biochemistry, Hanoi Medical University, No 1, Ton That Tung St., Dong Da, Hanoi 100000, Vietnam.; 8 Department of Microbiology and Immunology, Chonnam National University Medical School, Gwangju 61469, Republic of Korea.

**Keywords:** bacterial immunotherapy, *Salmonella typhimurium*, tumor microenvironment reprogramming, immune checkpoint blockade (ICB), DAMPs (damage-associated molecular patterns), single-cell RNA sequencing

## Abstract

**Rationale:**

The immunosuppressive tumor microenvironment (TME) remains a major barrier to the efficacy of immune checkpoint blockade (ICB) therapy, underscoring the need for strategies that can safely reprogram the TME to enhance cancer immunity.

**Methods:**

Here, we developed CNC018, a clinically translatable *Salmonella typhimurium* (*SL*) strain designed to reprogram the TME and potentiate responses to ICB. CNC018 was constructed using the ppGpp-defective *Salmonella* strain (*SL*∆ppGpp, Δ*relA/*Δ*spoT* deletion) as a genetic backbone*,* with the additional deletion of *Salmonella* pathogenicity island 1 and 2 (SPI-1 and SPI-2), which are essential for host cell invasion and intracellular survival. These modifications effectively eliminated the risk of virulence restoration.

**Results:**

CNC018 exhibited markedly reduced intracellular invasiveness, was rapidly cleared from non-tumor tissues, and displayed a 2.2-fold higher median lethal dose compared with *SL*∆ppGpp. Preferentially accumulating within tumors, CNC018 inhibited both primary and metastatic tumor growth in murine, human, and patient-derived xenograft models. CNC018 also induced tumor-derived damage-associated molecular patterns, which activated DCs and tumor-specific CD8⁺ T cells through the TLR4-NF-κB, JAK-STAT-IRF1, and NLRP3 inflammasome signaling pathways. Flow cytometry and single-cell RNA-sequencing revealed that CNC018 dramatically modulated immune checkpoint expression in the TME and tumor-draining lymph nodes, upregulating PD-L1 on tumor cells and dendritic cells and CTLA-4 on regulatory T cells, while downregulating PD-1 on effector T cells. This checkpoint modulation sensitized tumors to anti-PD-L1 and anti-CTLA-4 therapy, achieving synergistic tumor eradication and inducing durable, tumor-specific T-cell memory against tumor rechallenge.

**Conclusions:**

CNC018 represents a promising next-generation bacterial adjuvant with strong translational potential to safely enhance ICB efficacy in clinical cancer therapy.

## Introduction

Immune checkpoint blockade (ICB) therapies targeting regulatory proteins such as programmed death-ligand 1 (PD-L1), programmed cell death protein 1 (PD-1), and cytotoxic T-lymphocyte antigen 4 (CTLA-4) have revolutionized cancer immunotherapy, prolonging survival in patients with advanced malignancies 1. However, a significant proportion of patients exhibit poor or no response, remaining a major clinical challenge 2. ICB resistance is often driven by an immunosuppressive tumor microenvironment (TME), characterized by downregulated antigen presentation, impaired T-cell recognition, insufficient immune priming, and limited expression of immune checkpoint molecules 2. Therefore, safe and effective strategies to reprogram the TME are urgently needed to broaden the clinical benefit of ICB-based immunotherapy.

Induction of immunogenic cell death (ICD) is a clinically recognized mechanism that can help overcome tumor-induced immune suppression and restore effective anti-tumor immunity 3. Certain chemotherapeutic agents (e.g., oxaliplatin, anthracyclines) and specific types of radiotherapy have been shown to induce ICD, resulting in the release or exposure of damage-associated molecular patterns (DAMPs) such as calreticulin (CALR, an “eat-me” signal), adenosine triphosphate (ATP, a “find-me” signal), and high-mobility group box 1 (HMGB1, an “activate-me” signal), which serve as potent immunological signals. These molecules promote dendritic cell (DC) recruitment and maturation, thereby priming robust cytotoxic CD8^+^ T-cell responses that can break tumor-induced immune tolerance 3. However, the capacity of conventional chemotherapy and radiotherapy to elicit robust and durable ICD responses remains limited and highly context-dependent, often constrained by suboptimal DAMP release and concurrent immunosuppressive effects within the TME 4, 5. Moreover, although these therapies can remodel the TME by upregulating immune checkpoint molecules such as PD-L1 and PD-1 on tumor and immune cells 6, 7, this process may paradoxically reinforce local immunosuppression 5. Therefore, alternative therapeutic strategies capable of inducing potent ICD and favorably reprogramming the TME are needed to enhance the efficacy of ICB.

Attenuated strains of* Salmonella typhimurium* (*SL*) have emerged as promising immunotherapeutic agents owing to their intrinsic tumor tropism and potent immunostimulatory properties 8-10. The ppGpp-deficient strain, *SL*ΔppGpp, was generated by deleting the *relA* and *spoT* genes (*relA^-^spoT^-^*), which are required for the biosynthesis of ppGpp, a signaling molecule essential for the expression of virulence-associated genes such as those in the Type III secretion system (T3SS) 11. The LD_50_ value of *SL*ΔppGpp is approximately 10^5^-10^6^-fold higher than that of the wild-type strain 11, and we have utilized this strain in various forms for cancer therapy 12, 13. Because the T3SS, a needle-like apparatus used for infecting eukaryotic cells, is ppGpp-dependent, *SL*ΔppGpp exhibits defects in invasion and intracellular growth 11. The defect in intracellular invasion and survival is beneficial, as this strain can be easily cleared by macrophages in reticuloendothelial (RE) organs, where systemically administered bacteria initially and transiently localize 14. Nevertheless, a low yet detectable level of bacteria (10^3^-10^5^ CFU/g tissue) can transiently localize in non-tumor RE organs following systemic administration, where the bacteria may encounter host commensal microbiota, raising a theoretical concern of horizontal gene transfer 13, 15-17. To eliminate any theoretical possibility of virulence restoration arising from such events and to ensure maximal biosafety for clinical application, further genetic attenuation was pursued. These considerations demonstrate the importance of irreversibly attenuated *Salmonella* strains with enhanced safety profiles.

Here, we developed CNC018, a translational candidate* Salmonella typhimurium* strain derived from *SL*ΔppGpp with additional deletions of *Salmonella* pathogenicity island 1 (SPI-1) and 2 (SPI-2). These loci encode two distinct T3SSs that are critical for host cell invasion (SPI-1) and intracellular replication within phagocytic cells (SPI-2) 18. The complete removal of both SPI-1 and SPI-2 provides an additional layer of biosafety, eliminating the major virulence mechanisms and ensuring irreversible attenuation without compromising tumor-colonizing capacity. Moreover, CNC018 selectively colonizes tumors and reprograms the TME by inducing ICD with DAMP release, activating anti-tumor immune responses via TLR4-NF-κB signaling, JAK-STAT-IRF1-dependent checkpoint induction, and NLRP3 inflammasome activation, thereby synergizing with ICB to induce complete tumor regression. Collectively, CNC018 represents a safe, highly potent, and clinically translatable bacterial immunotherapy platform for cancer treatment.

## Materials and Methods

### Bacterial strains, plasmids, and cancer cell lines

Wild-type *S. typhimurium* strain 14028S was obtained from the American Type Culture Collection (ATCC), USA. The *SL*∆ppGpp strain, deficient in ppGpp biosynthesis, was engineered by deleting the *relA* and *spoT* genes, which encode its synthases 11. The bioluminescent strain* SL∆*ppGpp-*lux* was generated via phage transduction of the *lux* operon 11, 19. Plasmids used for homologous gene deletion, including pKD13, pKD46, and pCP20, were provided by Professor Hyon E Choy, Department of Microbiology, Chonnam National University Medical School, Korea.

Murine tumor cell lines CT26 (colon carcinoma; ATCC CRL-2638, RRID: CVCL_7256), 4T1 (breast carcinoma; ATCC CRL-2539, RRID: CVCL_0125), 4T1-Luc2 (ATCC CRL-2539-LUC2, RRID: CVCL_A4BM), and B16F10 (melanoma; ATCC CRL-6475; RRID: CVCL_0159) were purchased from ATCC (USA). MC38 cells (murine colon adenocarcinoma; Kerafast ENH204-FP, RRID: CVCL_B288) were obtained from Kerafast (USA). HepG2-Luc cells (Cat# HB-806, RRID: CVCL_JG47) and MDA-MB-231-Luc-GFP cells (Cat# SC044, RRID: CVCL_0062) were sourced from GenTarget Inc. (USA).

CT26, MC38, and B16F10 cells were maintained in Dulbecco’s Modified Eagle’s Medium (DMEM) (Welgene, Korea). RPMI-1640 medium (Welgene, Korea) was used for 4T1, 4T1-Luc2, and MDA-MB-231-Luc-GFP cells, whereas HepG2-Luc cells were grown in Minimum Essential Medium (MEM) (Welgene, Korea). Each culture medium was supplemented with 10% fetal bovine serum (FBS) and 1% penicillin-streptomycin. All cell lines were propagated at 37 °C with 5% CO_2_ and were screened for mycoplasma contamination using a PCR-based assay kit (Intron Biotechnology, Korea) before experimental use.

### Generation and characterization of CNC018

CNC018 was generated from the *SL*ΔppGpp background by sequential deletion of SPI-1 and SPI-2. SPI-1 and SPI-2 encode the T3SS-1 and T3SS-2 required for host-cell invasion and intracellular replication, respectively. SPI-1 was deleted from *SL*ΔppGpp using the λ Red recombination system carried by the helper plasmid pKD46. Briefly, homologous recombination using PCR amplicons generated from the pKD13 template, with primers targeting the first gene (*STM14_3500*) and the last gene (*sitD*) of the locus. Purified PCR products were introduced into *SL*ΔppGpp by electroporation, and transformants were selected on kanamycin (50 µg/mL) at 37 °C. Candidate colonies were screened by PCR using locus-spanning primers (Table S2). The pKD46 helper plasmid was subsequently cured by incubation at 42 °C, and the kanamycin-resistance cassette was excised via FLP recombinase after transformation with the temperature-sensitive plasmid pCP20.

SPI-2 was deleted in the SPI-1-deleted background using the same λ Red recombination protocol, with primers targeting the first gene (*ydhE*) and the last gene (*orf242*) of the SPI-2 locus. The resulting strain, designated CNC018, carries deletions of 73 genes relative to the wild-type *SL*14028S genome, comprising two ppGpp biosynthesis genes, 38 SPI-1 genes, and 33 SPI-2 genes. Successful genome modification was confirmed by whole-genome sequencing (Macrogen, Korea).

CNC018-*lux*, *SL*∆ppGpp*-lux*, and VNP20009-*lux* were generated by phage transduction with the *lux* operon 11, 19. These strains were used for bioluminescence imaging.

### Bacterial genotyping and growth

CNC018 was genotyped using multiplex polymerase chain reaction (PCR) with primers specific for *relA*, *spoT*, *SpaO* (SPI-1), and *sseF* (SPI-2), using the AccuPower PCR PreMix kit (Bioneer, Korea) (Table S2). A single colony was directly transferred into reaction mixtures containing premix, primer pairs, and distilled water. Thermal cycling (TP600 thermocycler, Takara Bio, Japan) comprised initial denaturation, then 40 cycles of denaturation, annealing, and extension. PCR products were loaded on agarose gels containing NEOgreen dye (Kangsan Science, Korea) and separated by electrophoresis. DNA was visualized under UV light and imaged using the GDS-200C digital imaging system (Labtech, Korea).

To assess bacterial growth in rich and minimal media, bacteria from frozen glycerol stocks were streaked on agar plates containing Luria-Bertani (LB) (Difco, USA) or M9 minimal media (Welgene, Korea) and incubated at 37 °C overnight. The bacterial colonies were observed the following day.

### Measurement of bacterial growth curve

A single bacterial colony from an overnight LB agar plate was inoculated into LB broth and cultured at 37 °C with shaking. The overnight culture was diluted 100-fold into fresh LB broth and sub-cultured at 37 °C with shaking for 24 h. The optical density at 600 nm (OD_600_) was measured hourly or every 2 h using a spectrophotometer.

### Bacterial invasion assay

CT26 cells (5 × 10^5^ cells/well) were seeded in 6-well plates with DMEM supplemented with 10% FBS and 1% penicillin-streptomycin and incubated at 37 °C in 5% CO_2_ for 24 h. Overnight bacterial cultures were diluted 100-fold in fresh LB broth and grown to early stationary phase (OD_600_ 2-2.2). Bacteria were centrifuged at 4,000 rpm for 4 min and washed twice with phosphate-buffered saline (PBS). The bacterial pellet was resuspended in PBS and added to CT26 cells at a multiplicity of infection (MOI) of 10:1. After 30 min of incubation at 37 °C in 5% CO₂, wells were washed three times with PBS, and fresh medium containing gentamicin (150 μg/mL; Sigma, USA) was added for 60 min. Cells were lysed with 0.05% Triton X-100 in PBS (pH 7.4), and intracellular bacteria were plated on brain heart infusion agar (BD DIFCO, Korea). Bacterial colonies were counted the next day after incubation at 37 °C.

### Minimum Inhibitory Concentration (MIC) testing

Female BALB/c mice were subcutaneously injected in the right flank with 5 x 10^5^ of CT26 cells. When tumors reached a volume of approximately 100 mm³, mice received a single intravenous injection of 2 x 10^7^ CFU of CNC018 suspended in 100 μL of sterile saline. To assess phenotypic stability within the TME, tumors were harvested on days 1, 7, 14, and 21 post-administration (*n* = 3 mice/time point). Tissues were mechanically homogenized (Precellys Evolution, Bertin Technologies) and plated on LB agar. To evaluate antibiotic susceptibility, five random colonies per tumor were isolated and uniformly swabbed onto fresh LB plates. Minimum inhibitory concentrations (MIC) were determined using MIC test strips (KisanBio, Korea) for amoxicillin, ciprofloxacin, ceftriaxone, and cefixime following a 16 h incubation at 37 °C. *Ex vivo* MIC profiles were compared directly against the pre-administration CNC018 parental strain to confirm retained antibiotic susceptibility and verify the absence of acquired resistance.

### Measurement of viability, DAMPs, and cytokines in tumor cells treated with bacteria

Tumor cells (5 × 10^5^) were seeded into a 6-well plate containing 1 mL of medium with 10% FBS (no antibiotics) and cultured at 37 °C overnight. The cells were then treated with CNC018 (5 × 10^6^ colony-forming units [CFU]) and further cultured for 18 h. Culture supernatants were collected by centrifugation at 1,200 rpm for 5 min and filtered by a 40-µm nylon membrane strainer (SPL, Korea). Tumor cells were detached using 200 µL of 0.05% Trypsin/EDTA (1X) per well (Welgene, Korea) and washed twice with ice-cold PBS.

Cell viability was assessed by measuring lactate dehydrogenase (LDH) activity with the LDH cytoTox 96 Non-Radioactive Cytotoxicity Assay kit (Promega, USA). A 50 µL aliquot of culture supernatant was used to determine LDH activity. For the maximum LDH release control, an equivalent volume of supernatant was incubated with 10 µL of 10x lysis buffer (provided in the kit) for 45 min at 37 °C. The OD value was measured at 490 nm and 492 nm on a VersaMax microplate reader (Molecular Devices, USA). Results were normalized by the total protein amount. ATP quantification was performed by using an ATP commercial kit (Abcam, UK) following the manufacturer's protocol.

Released HMGB1 in culture supernatants was analyzed by Western blotting. Culture supernatant (20 µg of total protein) was loaded onto 12% sodium dodecyl sulfate polyacrylamide gel electrophoresis (SDS-PAGE) and separated by electrophoresis. Proteins were then transferred to a nitrocellulose membrane (GE Healthcare, USA) and blocked for 2 h at room temperature in 5% (w/v) skim milk in Tris-buffered saline with 0.1% Tween-20 (TBS-T; Sigma-Aldrich, Germany). The membrane was incubated with primary αHMGB1 antibody (1 µg/mL in blocking buffer) for 2 h. After three washes with TBS-T, the membrane was incubated with horseradish peroxidase (HRP)-conjugated secondary antibody for 1 h. After three additional washes with TBS-T, chemiluminescent HRP substrate (Merck Millipore, USA) was added, and specific protein bands were visualized using a ChemiDoc^TM^ XRS+ system imager (Bio-Rad, USA).

Surface CALR and additional tumor-cell markers were evaluated by flow cytometry after staining with PE-conjugated antibodies. An isotype-matched control defined background fluorescence. A FACS Canto cytometer (BD Biosciences, USA) captured ≥ 10,000 events/specimen; data analysis utilized Flowjo software (BS Biosciences, USA).

Antibody information is summarized in Table S3.

### Bacterial toxicity in mice

Female 6-week-old BALB/c mice (Orient, Korea) were fasted overnight prior to bacterial injection. To determine 50% lethal dose (LD_50_) values, groups of four animals received an i.v. injection of bacteria. Mortality was monitored for 30 days, and LD_50_ values were calculated by the Reed and Muench method 20.

### Animal models and tumor establishment

Mouse strains originated from multiple sources: female BALB/c, C57BL/6, BALB/c athymic nu/nu, and TLR4-deficient (TLR4^-/-^) mice (C57BL/6 background) from Orient (Korea); female TLR5-deficient (TLR5^-/-^) mice (C57BL/6 background) provided from Prof. Shee Eun Lee, (Chonnam National University); and female NSG mice (NOD-SCID IL2Rγnull) from the Jackson Laboratory. The mice (6-week-old, ~19 g) were maintained for a week at a specific pathogen-free facility. The mice were randomly selected from different cages and housed together in a new cage per group, with each mouse used only once per experiment. The Chonnam National University Animal Ethics Committee approved all protocols (CNU IACUC-H-2026-3) following institutional welfare guidelines 21.

Syngeneic tumor models employed isoflurane anesthesia (2%) for s.c. flank implantations: CT26 or MC38 cells (5 × 10^5^) into BALB/c or C57BL/6 mice, respectively; 4T1-Luc2 cells (5 × 10^5^) into BALB/c or athymic nu/nu BALB/c mice. B16F10 melanoma utilized either s.c. (5 × 10^5^ cells) or i.v. (2.5 × 10^5^ cells) routes in C57BL/6 mice. Xenograft models included HepG2-Luc cells (3 × 10^5^, i.p.) in athymic nu/nu BALB/c mice for liver metastasis and MDA-MB-231-Luc-GFP cells (1 × 10^6^, mammary fat pad) in NSG mice for breast cancer with metastatic capacity.

Checkpoint blockade employed i.p. administration of anti-PD-L1, anti-PD-1, or anti-CTLA-4 antibodies (200 μg in 200 μL PBS), started 1 day prior to bacterial treatment and continued twice weekly for a total of five doses.

Patient-derived xenograft (PDX) was established as described 22. Fresh colon cancer specimens obtained from Chonnam National University Hwasun Hospital were processed to establish patient-derived xenografts (Table S4). Tumor tissues were either implanted immediately after resection or maintained briefly in ice-cold DPBS before implantation. Samples were minced into small fragments and inserted subcutaneously into NSG mice within 60 min. Once tumors reached 2 - 3 cm^2^, animals were euthanized, and the tumor pieces were transplanted into new recipient mice for expansion. Tumor material was additionally characterized by flow cytometry, histology, and molecular profiling when feasible and stored in DMEM containing 20% FBS and 10% DMSO in liquid nitrogen for future use. After three passages, cryopreserved PDX tumor cells were thawed, washed, suspended in PBS, and implanted subcutaneously into NSG mice at 1 × 10^6^ cells per mouse. Twenty-one days later, animals were treated intravenously with 2 × 10^7^ CFU of CNC018. Tumor growth and immune profiling were then assessed 3 days after bacterial administration.

Overnight bacterial cultures underwent 1:100 dilution in fresh LB broth and expansion to early stationary growth (OD_600_ = 2.0 - 2.2). Cultures were pelleted by centrifugation (4,000 rpm, 4 min), washed with PBS, and resuspended in sterile PBS (100 µL final volume) to achieve 2 × 10^7^ or 1 × 10^8^ CFU per injection (calibration: OD_600_ of 1.0 = 8 × 10⁸ CFU/mL).

Tumor size (mm³) was measured every three days using the equation (length × height × width)/2. According to the guidelines of the Chonnam National University Institutional Animal Care and Use Committee, mice were sacrificed when tumor size reached ≥ 1500 mm³. In rare cases where this threshold was exceeded, no mice remained above it for more than 3 days. Mice showing pain, discomfort, or distress were immediately euthanized. Experiments and analyses were blinded to minimize bias.

### Single-cell sequencing

Single-cell sequencing was performed on solid tumor-derived mononuclear cells, including both tumor cells and tumor-infiltrating immune cells, isolated from CT26 tumor-bearing mice treated with PBS or CNC018. Tumors were excised on day 3 post-treatment and transferred to Humanizing Genomics Macrogen for single-cell sequencing.

### Single-cell RNA-seq (scRNA-seq) analysis

Raw count matrices from 10x Genomics were processed using Seurat v5.0.0 23, 24 in R. Low-quality cells were excluded if *nFeature_RNA* ≤ 500, *nFeature_RNA* ≥ 8,000, or mitochondrial gene content ≥ 5%. Doublets were detected and removed using scDblFinder v1.14.0 25.

Each dataset was log-normalized, and 2,000 highly variable genes were identified using the “vst” method. Data were scaled, and principal component analysis (PCA) was performed using the top 50 principal components. Batch effects were corrected with Harmony v0.1.1 26. Graph-based clustering was conducted using the Louvain algorithm at resolutions ranging from 0.2 to 1.2. Dimensionality reduction for visualization was performed using UMAP on the Harmony-corrected embeddings.

To quantify mRNA expression from the scRNA-seq data, we calculated the percentage of the total cell population and of the *Cd3d^+^* T cell compartment exhibiting detectable transcript levels (log-normalized expression > 0). All results were summarized and visualized using ggplot2 v3.4.4 27. Given the limited number of biological replicates (*n* = 2 pooled tumors/group), these scRNA-seq findings are presented as descriptive transcriptional snapshots intended to support and refine the broader immunophenotypic changes observed via flow cytometry.

### Gene module score calculation

Gene module scores were computed by taking the mean expression of the genes within each module and subtracting the mean expression of a match reference gene set, in which control genes were randomly sampled from a predefined gene pool and binned by similar average expression. For each module gene set, including the TLR4-NF-κB, JAK-STAT-IRF1, NLRC3 inflammasome, and cGAS-STING signaling pathways, module scores were calculated using the *AddModuleScore* function in Seurat 24.

### Evaluation of synergism for the combination of CNC018 and ICB

To determine whether CNC018 and ICB therapy produced synergistic anti-tumor effects, we calculated the coefficient of drug interaction (CDI) according to the formula: CDI = (AB/control)/[(A/control) × (B/control)]. In this equation, A and B denote individual treatment effects (CNC018 alone or ICB alone), while AB represents the dual-therapy outcome, each normalized to untreated controls. Synergistic interactions were defined as CDI < 1; additive effects as CDI = 1; and antagonistic interactions as CDI > 1. We considered CDI values below 0.7 to reflect strong synergy. For these computations, average tumor size at day 12 (CT26 tumors) or day 9 (MC38 tumors) served as input data.

### Statistical analysis

Statistical analysis was performed using Prism 9.0 software (GraphPad, USA), with *P-*values < 0.05 considered significant. Statistical tests and *P*-values are detailed in figure legends. Student’s *t*-test was used for single-variable comparisons, two-way ANOVA with Tukey’s or Sidak’s correction for multiple comparisons, and Kaplan-Meier curves with log-rank (Mantel-Cox) tests for survival analysis. Data are presented as mean ± standard error of the mean (s.e.m.).

## Results

### Generation and characterization of the CNC018 strain

**Figure 1:**
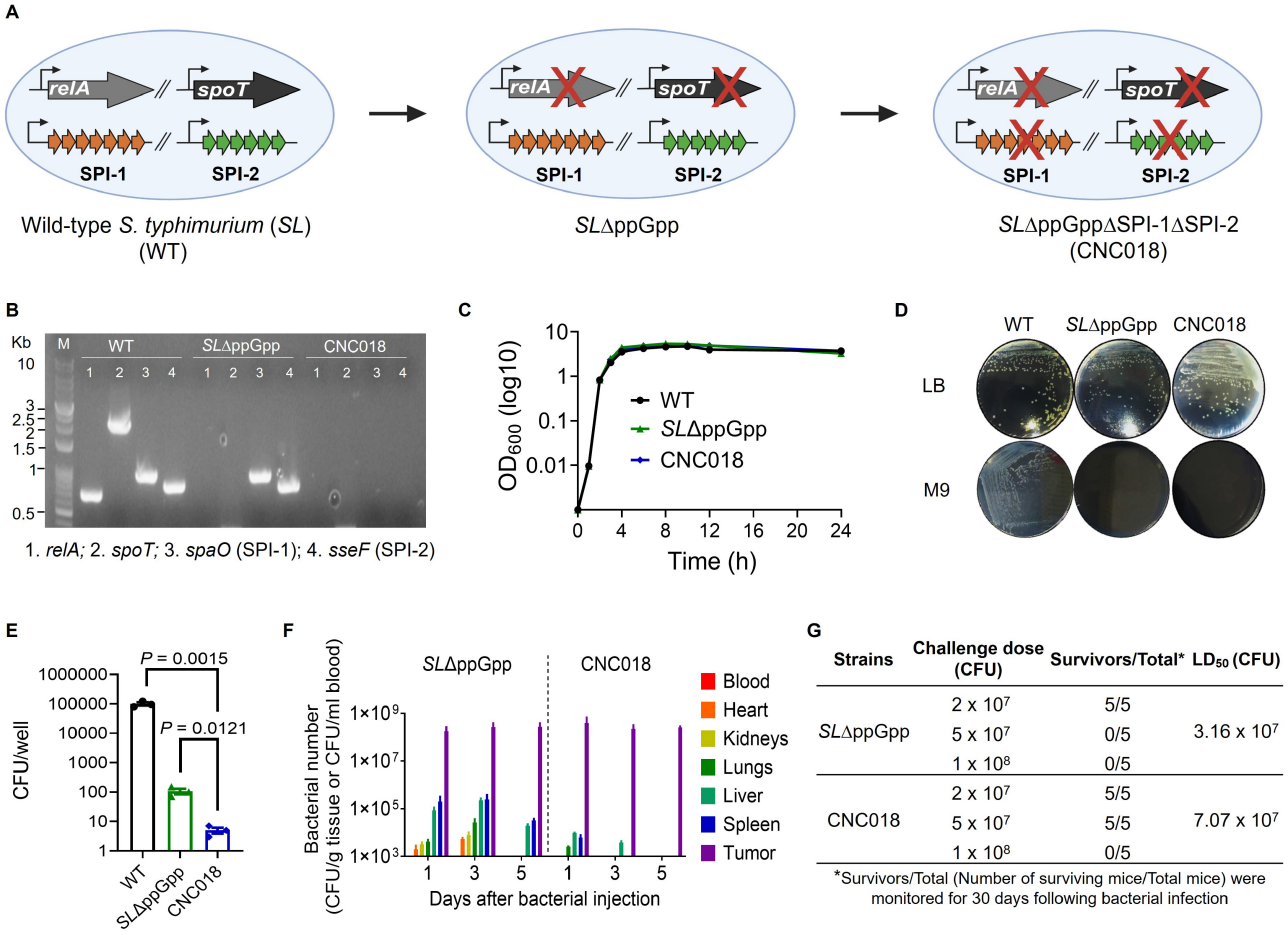
** Generation and characterization of CNC018 (*SL*∆ppGpp∆SPI-1∆SPI-2).** (**A**) Generation of CNC018. The* SL*∆ppGpp strain, deficient in the synthesis of the virulence gene regulator guanosine 5’-diphosphate 3’-diphosphate (ppGpp), was generated by deleting the *relA* and *spoT* genes from wild-type *Salmonella typhimurium* 14028S (WT). Subsequently, the SPI-1 and SPI-2 gene clusters, encoding invasion and intracellular proliferation, respectively, were deleted from *SL*∆ppGpp to generate CNC018. Black crosses (X) indicate gene deletions. (**B**) Genotyping of CNC018. PCR analysis of bacterial colonies grown on LB agar was performed using primers specific for *relA*, *spoT*, *spaO* (SPI-1), and *sseF* (SPI-2). Expected PCR product sizes were 715 bp (*relA*), 2,185 bp (*spoT*), 990 bp (*spaO*), and 843 bp (*sseF*). M, size marker. (**C**) Growth curve of CNC018. Bacteria were cultured in LB broth at 37 °C, and OD_600_ was measured at the indicated time points (dots) for 24 h (*n* = 3 independent experimental replicates). (**D**) Phenotyping of CNC018. Bacteria were streaked on LB agar (top) or M9 minimal agar (bottom) plates and incubated (37 °C, 18 h). (**E**) Bacterial invasion assay. MC38 cells (5 × 10⁵) were co-cultured with CNC018 at MOI10 for 1 h, followed by gentamicin treatment (150 µg/mL) for 1 h. Cell lysates were plated on LB agar, and bacterial colonies were counted (37 °C, 18h) (*n* = 3 technical replicates; unpaired two-tailed *t*-test). (**F**) Biodistribution of CNC018 in CT26 tumor-bearing mice. BALB/c mice bearing s.c. tumors (~120 mm³) received *SL*∆ppGpp or CNC018 via i.v. injection (2 × 10⁷ CFU/mouse, *n* = 3 mice/group) (day 0). At the indicated time points, bacteria were isolated from tumors and normal organs, plated on agar, and enumerated (37 °C, 18h). (**G**) Median lethal dose (LD_50_) in mice following bacterial infection. BALB/c mice (*n* = 5 mice/group) were treated with *SL∆*ppGpp or CNC018 (2 × 10^7^ to 1 × 10^8^ CFU) via i.v. injection. Mouse survival was monitored for 30 days to determine the LD_50_ using the Reed and Muench method.

We first confirmed the targeted gene deletions in CNC018 by polymerase chain reaction (PCR) using primers specific to *relA*, *spoT*, SPI-1, and SPI-2 (Figure 1A-B; Table S1, S2). Whereas wild-type *SL*14028S (WT) yielded amplicons for all four PCR bands (*relA*, *spoT*, *spaO* (SPI-1), and *sseF* (SPI-2)), CNC018 showed no detectable bands, confirming complete deletion of *relA*/*spoT* and the entire SPI-1 and SPI-2 (Figure 1B). These deletions were further validated by whole-genome sequencing (see data viability). Loss of SPI-1 and SPI-2 did not impair growth in nutrient-rich conditions; CNC018 proliferated comparably to WT and *SL*∆ppGpp in Luria-Bertani (LB) broth at 37 °C (Figure 1C). However, CNC018 failed to grow on M9 minimal agar, mirroring the auxotrophy of *SL*∆ppGpp 11 (Figure 1D). Consistent with a prior report 28, *SL*∆ppGpp exhibited a 10^3^-fold reduction in invasion of CT26 cells relative to the WT strain. CNC018 exhibited more attenuated invasiveness, with invasion efficiency reduced by over 10-fold *in vitro* (Figure 1E) and approximately 3-fold *in vivo* compared to *SL*∆ppGpp (Figure S1), attributable to the deletions of SPI-1 and SPI-2. Despite its attenuated invasiveness, CNC018 maintained intratumoral colonization and reached a steady-state plateau comparable to those of *SL*ΔppGpp and the T3SS-intact VNP20009 strain (Figure 1F; Figure S1, S6B). These data support that T3SS-mediated cellular invasion is not a rate-limiting determinant of tumor targeting, colonization, and intratumoral proliferation 29-31. Together, our findings indicate that CNC018 effectively colonizes and proliferates within tumors without requiring entry into host cells.

For successful clinical translation, CNC018 must retain both absolute genomic integrity and a stable phenotypic attenuation. We therefore evaluated its stability under cumulative selective pressure *in vitro* and *in vivo* contexts (Figure S2A). PCR-based genotyping confirmed the definitive retention of all four engineered deletions (*ΔrelA, ΔspoT, ΔspaO* (SPI-1)*,* and *ΔsseF* (SPI-2)) throughout 30 serial passages in non-selective LB broth (Figure S2B, top). To stimulate a high-risk environment permissive for selection-driven bacterial evolution, we performed 5 serial passages of CNC018 in immunodeficient NSG mice, which lack the selective pressure of adaptive immunity. Recovered isolates exhibited no evidence of genotypic reversion (Figure S2B, bottom), confirming the inherent stability of the irreversible chromosomal deletions via the λ Red recombination system. This phenotypic stability was further corroborated by preservation of metabolic auxotrophy. Although passaged isolates exhibited growth kinetics in nutrient-rich media comparable to the parental strain, they remained unable to proliferate in minimal M9 medium (Figure S2C). Critically, these isolates retained potent anti-tumor efficacy (Figure S2D) and specific tumor tropism, with intratumoral tumor densities exceeding those in the liver or spleen by over 1,000-fold (Figure S2E). Furthermore, CNC018 remained highly susceptible to a panel of clinically relevant antibiotics, with minimum inhibitory concentration (MIC) remaining stable (Figure S2F). Collectively, these data demonstrate that CNC018 possesses the genetic and phenotypic robustness required to prevent pathogenic recrudescence, establishing a favorable safety profile for human clinical trials.

We next directly compared the biodistribution of CNC018 with that of *SL*∆ppGpp in CT26 subcutaneous (s.c.) tumor-bearing BALB/c mice following intravenous (i.v.) injection. CNC018 accumulated in tumors at levels comparable to those of *SL*∆ppGpp at 1, 3, and 5 days post-injection (dpi) (Figure 1F). In contrast, CNC018 exhibited significantly accelerated clearance from normal tissues, including the spleen, liver, kidney, lung, heart, and blood, compared to *SL*∆ppGpp. Notably, by day 5 post-injection, no bacteria were detectable in any organs other than the tumor, suggesting a favorable safety profile and preferential tumor localization. These findings were further validated through *in vivo* and *ex vivo* bioluminescence imaging using *lux*-expressing strains (*SL*ΔppGpp-*lux* and CNC018-*lux*), which revealed equivalent tumor-specific signals for both strains, whereas detectable signals in the liver and spleen were uniquely restricted to *SL*ΔppGpp-*lux* (Figure S3A). Accordingly, CNC018-treated mice exhibited diminished body weight loss and attenuated splenomegaly compared to those receiving *SL*ΔppGpp (Figure S3B-C), despite comparable inhibition of tumor growth across CT26, MC38, and 4T1-Luc2 tumor models (Figure S3D-F). Additionally, CNC018 showed a 2.2-fold higher median lethal dose (LD_50_) than *SL*∆ppGpp in BALB/c mice via i.v. injection (3.16 × 10^7^ CFU for *SL*∆ppGpp versus 7.07 x 10^7^ CFU for CNC018), with no evidence of systemic toxicity (Figure 1G, Figure S4). The long-term safety profile of CNC018 was further confirmed by its rapid clearance of CNC018 from healthy organs and the absence of significant weight loss, organomegaly, or histopathological injury over the follow-up period (Figure S5A-D). Furthermore, CNC018 did not elicit the hyper-inflammatory cytokine storm typically associated with systemic bacterial administration (Figure S5E). This favorable safety profile remained consistent even under a repeated-dose regimen (three therapeutic cycles), after which mice displayed stable body weight, 100% survival, and baseline serum transaminase levels (ALT/AST), thereby confirming the high tolerability of cumulative dosing (Figure S5F-I). To further examine its translational potential, we benchmarked CNC018 against VNP20009, a *S. typhimurium* strain previously evaluated in clinical trials 32. While CNC018 exhibited tumor-targeting capacity and therapeutic efficacy equivalent to VNP20009 (Figure S6A-C), its safety profile was markedly superior (Figure S6D-G). Notably, whereas VNP20009-treated mice suffered from persistent weight loss, chronic splenomegaly, and hepatomegaly, CNC018-treated mice recovered rapidly to baseline and showed no signs of organomegaly or elevated serum transaminases (Figure S6D-F). Crucially, the LD_50_ for CNC018 was 7.94-fold higher than that of VNP20009, confirming a substantially broadened therapeutic window that supports its clinical viability (Figure S6G). Collectively, these findings indicate that CNC018 retains tumor-targeting and anti-tumor efficacy comparable to *SL*∆ppGpp and VNP20009, while achieving faster clearance from normal tissues and superior systemic safety.

### CNC018 reprograms the tumor immune microenvironment

**Figure 2:**
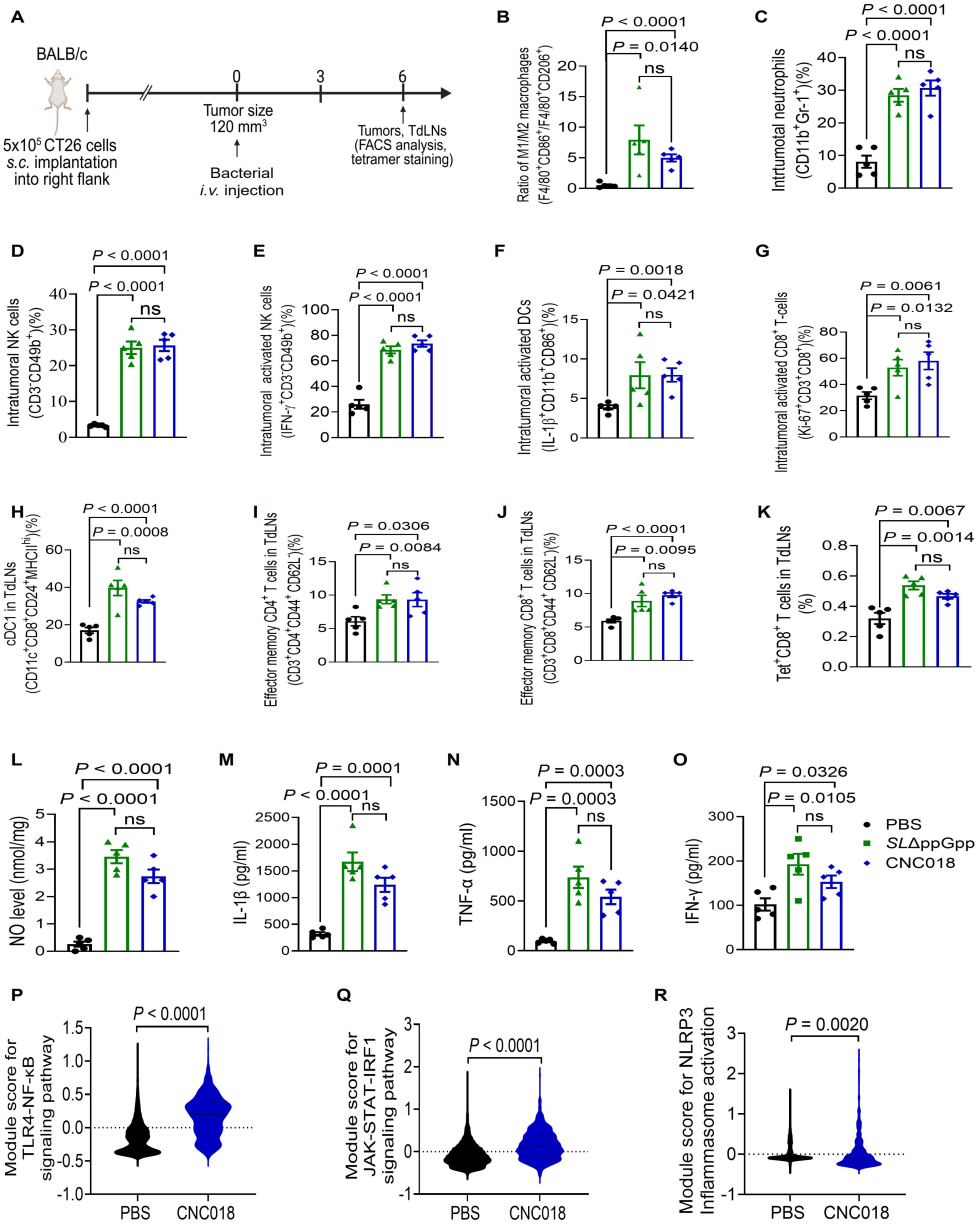
** Characteristics of anti-tumor immune responses induced by CNC018.** (**A**) Experimental scheme. BALB/c mice were implanted s.c. with CT26 cells. When tumors reached 120-140 mm^3^ (day 0), mice were treated with PBS, *SL∆*ppGpp, or CNC018 (2 × 10⁷ CFU/mouse) via i.v. injection. TdLNs were collected on day 3 or 6 for FACS, cytokine, and scRNA-seq analysis. (**B**) Ratio of intratumoral M1 macrophages (F4/80⁺CD86⁺) to M2 macrophages (F4/80⁺CD206⁺). (**C-K**) Immune cell profiles. (**C**) Frequency of intratumoral neutrophils (CD11b⁺Gr-1⁺). (**D**) Frequency of intratumoral NK cells (CD3⁻CD49b⁺). (**E**) Frequency of intratumoral activated NK cells (IFN-γ⁺CD3⁻CD49b⁺). (**F**) Frequency of intratumoral activated DCs (IL-1β^+^CD11b⁺CD86⁺). (**G**) Frequency of intratumoral proliferating CD8⁺ T cells (Ki-67⁺CD3⁺CD8⁺). (**H**) Frequency of cDC1 cells in TdLNs (CD11b⁺CD8⁺CD24⁺MHCII^hi^). (**I**) Frequency of effector memory CD4⁺ T cells in TdLNs (CD3⁺CD4⁺CD44⁺CD62L^-^). (**J**) Frequency of effector memory CD8⁺ T cells in TdLNs (CD3⁺CD8⁺CD44⁺CD62L⁻). (**K**) Frequency of tumor-specific CD8^+^ T cells in TdLNs (H-2L^d^ MuLV gp70 tetramer^+^ CD8^+^). (**L-O**) Intratumoral cytokine profiles. CT26 tumors were harvested and homogenized at 2 dpi. Supernatants were analyzed by ELISA assay for (**L**) NO, (**M**) IL-1β**,** (**N**) TNF-α, and (**O**) IFN-γ. *P*-values in B-O were determined by an unpaired two-tailed *t*-test (*n* = 4 - 5 mice/group; ns, not significant). (**P-R**) CNC018-induced activation of pro-inflammatory signaling pathways. ScRNA-seq module score analysis of PBS- and CNC018-treated tumors harvested on day 3. Violin plots represent mRNA expression module scores for (**P**) TLR4-NF-κB signaling (*Tlr4, Myd88, Irak4, Nfκb*), (**Q**) JAK-STAT-IRF1 signaling (*Ifng*, *Jak1/2*, *Stat1/2*, *Irf1*), and (**R**) NLRP3 inflammasome activation (*Nlrp3*, *Casp1*, *P2xr7*) (*n* = 2 pooled tumors/group; unpaired two-tailed *t*-test).

To characterize the anti-tumor immune responses induced by CNC018, we performed immunophenotyping of tumor tissues and tumor-draining lymph nodes (TdLNs) in CT26 s.c. tumor-bearing BALB/c mice (Figure 2A). Compared to PBS controls, both *SL*ΔppGpp and CNC018 significantly increased the intratumoral M1/M2 macrophage ratio (F4/80^+^CD86^+^/F4/80^+^CD206^+^), as well as the frequencies of neutrophils (CD11b^+^Gr-1^+^), total NK cells (CD3^-^CD49b^+^), activated natural killer (NK) cells (IFN-γ^+^CD3^-^CD49b^+^), activated dendritic cells (DCs, IL-1β^+^CD11b^+^CD86^+^), and proliferating CD8^+^ T cells (Ki-67^+^CD3^+^CD8^+^) (Figure 2B-G, Figure S7). In TdLNs, *SL*ΔppGpp and CNC018 similarly expanded conventional type 1 dendritic cells (cDC1; CD11c^+^CD8^+^CD24^+^MHCII^hi^), effector memory CD4^+^ T cells (CD3^+^CD4^+^CD44^+^CD62L^-^), effector memory CD8^+^ T cells (CD3^+^CD8^+^CD44^+^CD62L^-^), and tumor-specific CD8^+^ T cells recognizing the H-2L^d^-restricted MuLV gp70 peptide (SPSYVYHQF Tet^+^CD8^+^ T cells) (Figure 2H-K, Figure S7**)**. Additionally, treatment with *SL*ΔppGpp or CNC018 also markedly elevated intratumoral levels of nitric oxide (NO) and pro-inflammatory cytokines, including IL-1β, TNF-α, and IFN-γ (Figure 2L-O). To dissect the transcriptional circuitry underlying this immune reshaping, we performed single-cell RNA sequencing (scRNA-seq). Analysis of module score revealed a significant enrichment of signatures associated with the TLR4-NF-κB (*Tlr4*, *Myd88*, *Irak4*, and *Nfκb*), JAK-STAT-IRF1 (*Ifngr1/2*, *Jak1/2*, *Stat1/2*, and *Irf1*), and NLRP3 inflammasome (*P2rx7, Nlrp3*, and *Casp1*) pathways in CNC018-treated tumors (Figure 2P-R, Figure S8A-C). Notably, no significant induction of the cGAS-STING pathway (*Cgas*, *Tbk1*, *Irf3*, and *Tmem173)* was observed, suggesting that CNC018 promotes anti-tumor immunity through a STING-independent mechanism, likely mediated by surface-exposed pathogen-related molecular pattern (PAMP) and inflammasome activation (Figure S8D-E). Transcriptomic analysis of tumor-infiltrating T cells (*Cd3d^+^*) following CNC018 treatment revealed elevated expression of activation/cytotoxicity markers (*Cd44*, *Gzmb*, and *Prf1*) and polyfunctional cytokines (*Tnf* and* Ifng*). This was accompanied by the upregulation of exhaustion markers (*Tox, Havcr2,* and* Lag3*) and the downregulation of progenitor markers (*Tcf7* and* Il2*) (Figure S9). This specific signature characterizes the CNC018-induced T-cell compartment as an activated yet exhausted population, indicating a checkpoint-regulated state that is primed for reinvigoration by ICB 33. Collectively, these results demonstrate that CNC018 effectively reprograms the immunosuppressive TME into an immunostimulatory state. Upon selective colonization, CNC018 orchestrates robust innate and adaptive anti-tumor immune responses with a potency comparable to that of the parent *SL*ΔppGpp strain.

### CNC018 induces ICD to promote DC maturation

**Figure 3:**
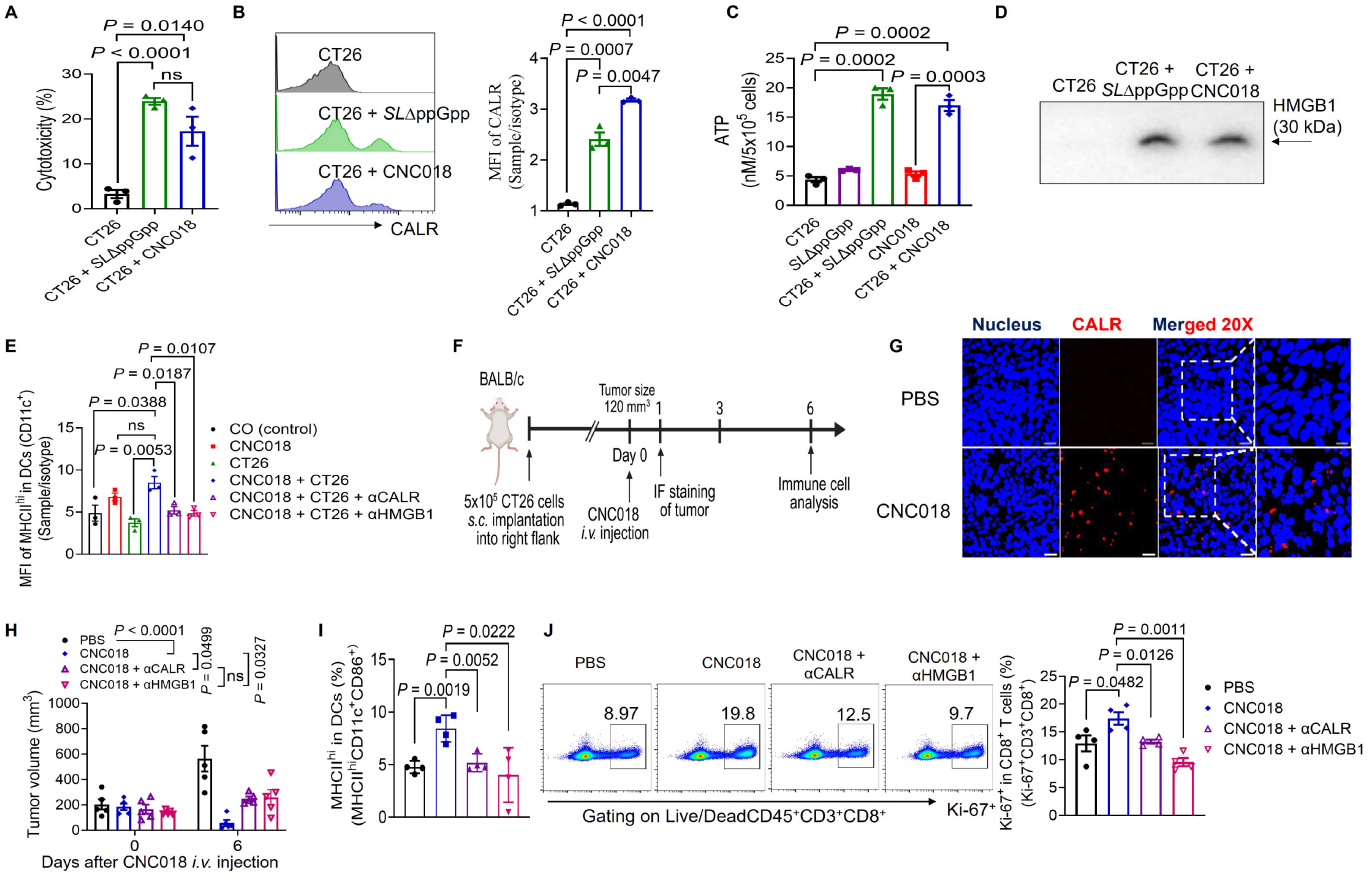
** DAMPs released from CNC018-induced dying tumor cells promote DC maturation**. CT26 tumor cells (5 × 10⁵ cells) were seeded overnight in 6-well plates. Cells were then co-cultured with *SL∆*ppGpp or CNC018 (MOI 10) for 20 - 24 h (A-D). (**A**) Cytotoxicity assay. Cytotoxicity was assessed by measuring lactate dehydrogenase (LDH) release (*n* = 3 technical replicates). (**B**) CALR exposure on tumor cells. Cells were stained with αCALR antibody or isotype control, and mean fluorescence intensity (MFI) was measured by FACS analysis (*n* = 3 technical replicates). (**C**) ATP secretion from tumor cells. ATP levels in culture supernatants were quantified (*n* = 3 technical replicates). (**D**) HMGB1 release from tumor cells. HMGB1 release in culture supernatants (20 µg total protein) was analyzed by SDS-PAGE and Western blotting using anti-HMGB1 antibody (αHMGB1) (three independent biological replicates). (**E**) *In vitro* DC maturation (MHCII^hi^CD11c⁺) triggered by CNC018-treated CT26 cells. CT26 cells were treated with CNC018 for 20 h, incubated with DAMP-neutralizing antibodies (αCALR or αHMGB1) for 6 h, and then co-cultured with immature bone marrow-derived DCs (imBMDCs). DC maturation (MHCII^hi^CD11c⁺ cells) was assessed by FACS. CO, CD11c⁺ imBMDCs only (*n* = 3 technical replicates; ns, not significant; unpaired two-tailed *t*-test). (**F**) *In vivo* experimental scheme (related to G-J). BALB/c mice bearing CT26 s.c. tumors (~120 mm³) were treated with CNC018 (2 × 10⁷ CFU) via i.v. injection starting on day 0. αCALR (28 µg) or αHMGB1 (20 µg) antibodies were injected i.p. (twice-weekly schedule) starting on day -1. Splenic immune cells were isolated on day 6 and analyzed by flow cytometry. (**G**) Immunofluorescence staining of CALR exposure in tumor tissues. CT26 tumors were excised 28 h after bacterial treatment, stained with DAPI (nuclei; blue) and αCALR (red) antibody, and imaged at both low (50 µm scale bar) and high (20 µm scale bar) magnification. (**H**) Average tumor volume (*n* = 5 mice/group; ns, not significant; two-way ANOVA with Tukey’s multiple comparisons test). (**I**) Frequency of activated splenic DCs (MHCII^hi^CD11c⁺CD86⁺). (**J**) Frequency of proliferating splenic CD8⁺ T cells (Ki-67⁺CD3⁺CD8⁺). *P*-values in A-C, I, and J were calculated using an unpaired two-tailed *t*-test (*n* = 4 mice/group; ns, not significant).

Given the immunomodulatory anti-tumor activity of *Salmonella* strains such as *SL*∆ppGpp 9, 10, 13, 34, we investigated whether CNC018 induces ICD capable of initiating adaptive immunity. CNC018 exhibited cytotoxicity against multiple murine cancer cell lines (CT26, MC38, 4T1, and B16F10) (Figure 3A, Figure S10A) and triggered the release of canonical ICD hallmarks, including surface-exposed CALR, secreted ATP, and released HMGB1 (Figure 3B-D, Figure S10B-D). Co-culture of CD11c^+^ bone marrow-derived DCs (CD11c^+^BMDCs) with CNC018-treated CT26 cells markedly promoted DC maturation (MHCII^hi^CD11c^+^), an effect that was abrogated by neutralizing anti-CALR (αCALR) or anti-HMGB1 (αHMGB1) antibody (Figure 3E). These findings are consistent with previous reports showing that CALR and HMGB1 from dying cancer cells drive DC maturation and antigen cross-presentation 4, which can subsequently induce PD-L1 expression on DCs 35.

We next examined the functional relevance of these DAMPs *in vivo* using CT26 tumor-bearing BALB/c mice (Figure 3F, Figure S11). CNC018 induced rapid CALR exposure on tumor cells within 28 h (Figure 3G). Tumor growth suppression by CNC018 was significantly attenuated on day 6 when αCALR or αHMGB1 antibodies were administered (Figure 3H). Consistently, blockade of CALR or HMGB1 markedly reduced splenic populations of activated DCs (MHCII^hi^CD11c^+^CD86^+^) and proliferating CD8^+^ T cells (Ki-67^+^CD3^+^CD8^+^) (Figure 3I-J). Collectively, these results demonstrate that CNC018 functions as a potent ICD inducer, releasing tumor-derived DAMPs that drive DC maturation and sustain downstream CD8^+^ T cell-mediated anti-tumor immunity.

### CNC018 induces innate immunity to inhibit primary and metastatic tumors

**Figure 4:**
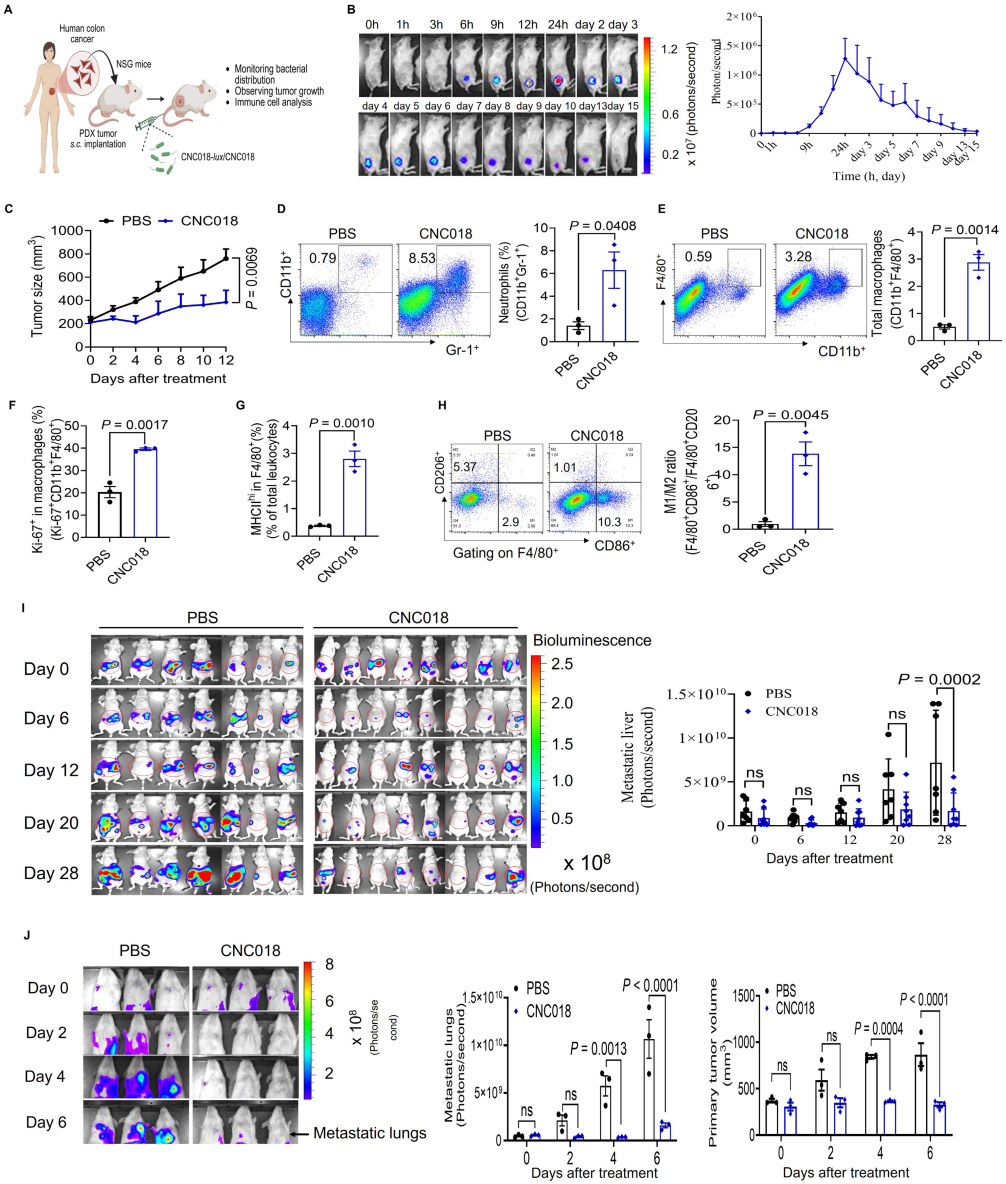
** CNC018 stimulates innate anti-tumor immunity.** (**A**) Experimental scheme. Colorectal adenocarcinoma cells from patients were inoculated s.c. into the right hind flank of NSG mice to establish the first-passage (P0) PDX tumors. Tumors reaching ~200 mm³ in volume were harvested, minced, and reimplanted into new recipient mice. After four passages (P3, ~200 mm³), mice received CNC018-*lux* (4 × 10⁷ CFU) or CNC018 (2 × 10⁷ CFU) via i.v. injection on day 0. Immune cells from PDX tumors were collected on day 3 for FACS analysis (*n* = 3 mice/group). (**B**) PDX tumor-targeting CNC018. Bioluminescence images were acquired at the indicated time points (h or days) after CNC018-*lux* injection. Left, representative mouse images; right, quantification of bioluminescence intensity in tumors (*n* = 3 mice/group). (**C**) PDX tumor growth curves. Mice bearing PDX tumors were treated with PBS or CNC018. Tumors was monitored and plotted as the average for each group (*n* = 3 mice/group). (**D-H**) Intratumoral immune cell profiles by flow cytometry (*n* = 3 mice/group; unpaired two-tailed *t-*test). (**D**) Frequency of neutrophils (CD11b⁺Gr-1⁺). (**E**) Frequency of total macrophages (CD11b⁺F4/80⁺). (**F**) Frequency of proliferating macrophages (Ki-67⁺CD11b⁺F4/80⁺). (**G**) Frequency of activated macrophages (MHCII^hi^F4/80⁺). (**H**) Ratio of M1-like macrophages (CD45⁺F4/80⁺CD86⁺) to M2-like macrophages (CD45⁺F4/80⁺CD206⁺). (**I**) CNC018 inhibits liver metastasis. Liver metastases were established by i.p. injection of HepG2-Luc cells into athymic *nu⁻/nu⁻* BALB/c mice. Three days after tumor implantation, CNC018 (2 × 10⁷ CFU) was administered via i.v. injection (day 0). *In vivo* bioluminescence imaging was visualized after i.p. injection of D-luciferin to assess FLuc activity. Left, whole-body images; right, bioluminescence intensity in metastatic livers (*n* = 7 - 8 mice/group; two independent experiments). (**J**) CNC018 inhibits lung metastasis. Lung metastases were established by mammary fat pad injection of human MDA-MB-231-Luc-GFP cells into NSG mice. CNC018 (2 × 10⁷ CFU) was injected i.v. into tumor-bearing mice when the primary tumor reached approximately 300 mm^3^ in volume. Fluc activity in metastatic lungs was measured by bioluminescence imaging after i.p. injection of D-luciferin (150 mg/kg). Left, representative images; middle, quantification of bioluminescence intensity of metastatic lungs; right, average primary tumor volume (*n* = 3 mice/group). *P*-values in C, I, and J were calculated using two-way ANOVA with Tukey’s or Sidak’s multiple comparisons test, respectively; ns, not significant.

To evaluate CNC018 efficacy in human-relevant models, patient-derived xenografts (PDXs) from human colon cancer were serially implanted into NOD-SCID IL2Rγ null (NSG) mice through four s.c. passages (P0-P3) (Figure S12, Table S4). On day 21 post-implantation (P3), mice received CNC018-*lux* or CNC018 (Figure 4A). Tumor-localized bioluminescence from CNC018-*lux* appeared at 6 h, peaked at 24 h, and remained detectable for up to 15 dpi (Figure 4B). Consistent with this, CNC018 treatment significantly delayed tumor growth compared with PBS control (Figure 4C). Immunophenotyping of the xenograft tumor tissues revealed a robust innate immune compartment typical of the NSG background. CNC018 treatment significantly increased the frequencies of neutrophils (CD11b⁺Gr-1⁺), total macrophages (CD11b⁺F4/80⁺), activated macrophages (Ki-67⁺CD11b⁺F4/80⁺, MHCIIʰⁱF4/80⁺, SIRPα⁺CD11b⁺F4/80⁺), TNF-α-producing M1 macrophages (TNF-α⁺F4/80⁺CD86⁺), and an increased M1/M2 macrophage ratio (F4/80⁺CD86⁺/F4/80⁺CD206⁺) (Figure 4D-H, Figure S13A-D). These changes were consistent with high levels of nitric oxide (NO) release (Figure S13E), which collectively induce tumor cell death and tumor inhibition 13. However, in the NSG model (lacking functional B, T, and NK cells), this innate response alone was insufficient to achieve durable tumor eradication. These findings highlight a critical mechanistic requirement: while CNC018-induced innate activation can suppress human tumor growth, the engagement of the adaptive immune arm is indispensable for sustained therapeutic response.

We next examined the ability of CNC018 to inhibit metastatic progression in immunocompromised mice. Human liver metastasis (HepG2-Luc, i.p. injection in BALB/c *nu/nu*), human breast cancer lung metastasis (MDA-MB-231-Luc-GFP, mammary fat pad in NSG), and murine breast cancer lung metastasis (4T1-Luc2, s.c. implantation in BALB/c *nu/nu*) models were established. CNC018 significantly inhibited both primary tumor growth and metastatic burden across all models (Figure 4I-J, Figure S14, S15). Altogether, these results demonstrate that CNC018 harnesses innate immunity to potently suppress both primary solid tumors and distant metastases, even in immunocompromised mice lacking T cells.

### CNC018 modulates immune checkpoint expression to synergize with ICB therapy

**Figure 5:**
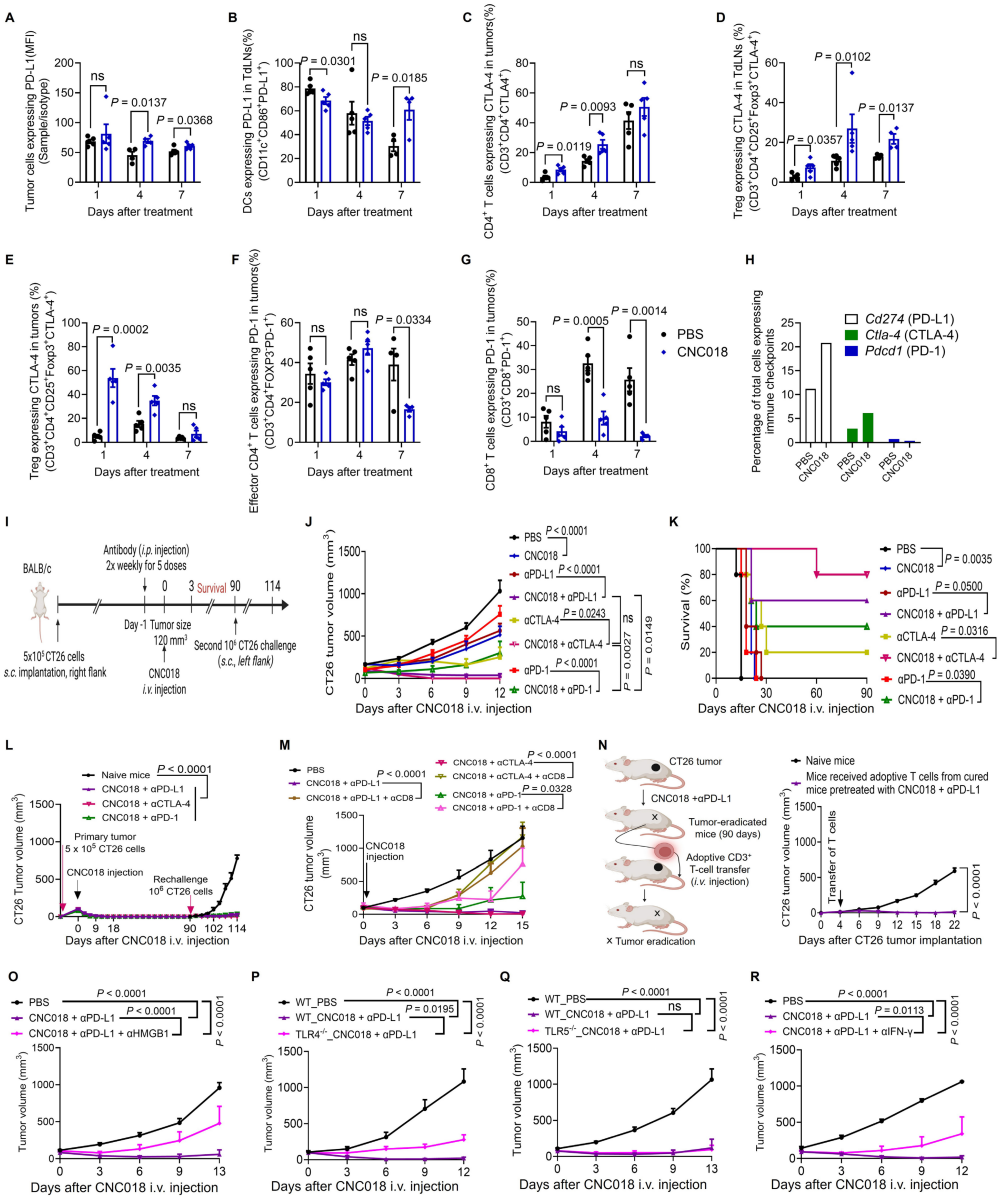
** CNC018 potentiates anti-tumor efficacy of ICB through generating tumor-specific T-cell memory.** (**A-G**) Flow cytometric analysis of checkpoint molecules on CT26 tumor cells and immune cells. CT26-bearing BALB/c mice (s.c. tumors, ~ 120 mm³, day 0) underwent tumors or TdLNs collected for flow cytometry analysis on days 1, 4, and 7 (*n* = 4 - 5 mice/group; ns, not significant; unpaired two-tailed *t*-test). (**A**) Frequency of tumor cells expressing PD-L1 (CD45^-^PD-L1^+^). (**B**) Frequency of DCs expressing PD-L1 in TdLNs (CD45⁺CD11c⁺CD86^+^PD-L1^+^). (**C**) Frequency of CD4+ T cells expressing CTLA-4 in tumors (CD45⁺CD3^+^CD4^+^CTLA-4^+^). (**D**) Frequency of Tregs expressing CTLA-4 in TdLNs (CD45⁺CD3^+^CD4^+^CD25^+^Foxp3^+^CTLA-4^+^). (**E**) Frequency of Tregs expressing CTLA-4 in tumors (CD45⁺CD3^+^CD4^+^CD25^+^Foxp3^+^CTLA-4^+^). (**F**) Frequency of effector CD4^+^ T cells expressing PD-1 in tumors (CD45⁺CD3^+^CD4⁺Foxp3^-^PD-1^+^). (**G**) Frequency of CD8^+^ T cells expressing PD-1 in tumors (CD45⁺CD3^+^CD8⁺PD-1^+^). (**H**) ScRNA-seq analysis of CT26 tumor-bearing BALB/c mice treated with PBS or CNC018 on day 3 (*n* = 2 pooled tumors/group). The percentages of cells expressing *Cd274* (PD-L1), *Ctla-4* (*CTLA-4*), and *Pdcd1* (PD-1) mRNA were quantified. CNC018-treated tumors showed increased *Cd274* and *Ctla-4* expression compared to PBS controls, whereas *Pdcd1* expression remained low in both groups. Data are presented as percentages of total cells. (**I**) Experimental scheme (related to J-L). CT26 tumor-bearing BALB/c mice were treated with CNC018 and/or ICB therapies (αPD-L1, αCTLA-4, or αPD-1). CNC018 (2 × 10⁷ CFU) was i.v. injected on day 0. ICB antibodies (200 µg/mouse, twice-weekly schedule for five doses) were i.p. injected starting on day -1. For CD8⁺ T-cell depletion, HMGB1 neutralization, or IFN-γ neutralization, anti-CD8 antibody (αCD8) (200 µg/mouse), anti-HMGB1 neutralizing antibody (αHMGB1) (20 μg/mouse), and anti-IFN-γ neutralizing antibody (αIFN-γ) (200 µg/mouse) were given i.p. a twice-weekly schedule for three to five doses. For tumor rechallenge experiments, CT26 tumor cells (1 × 10⁶) were s.c. reimplanted on the opposite flank of tumor-eradicated mice on day 90. Naïve age-matched control mice were also inoculated with CT26 cells (1 × 10⁶). (**J**) Average CT26 tumor growth curves in BALB/c mice (*n* = 5 mice/group). (**K**) Kaplan-Meier survival curves of CT26 tumor-bearing BALB/c mice [*n* = 5 mice/group; log-rank (Mantel-Cox) test]. (**L**) Average growth curves of primary and rechallenged CT26 tumors in BALB/c mice (*n* = 3 mice for CNC018 + αPD-L1; *n* = 4 mice for CNC018 + αCTLA-4; *n* = 2 mice for CNC018 + αPD-1). (**M**) Average CT26 tumor growth curves in mice treated with CNC018 + ICB after CD8⁺ T-cell depletion (*n* = 3 mice/group). (**N**) Adoptive T-cell transfer experiment in CT26 tumor-bearing mice after pretreatment with CNC018 and anti-PD-L1 therapy. Left, experimental scheme; right, average CT26 tumor growth curves (*n* = 5 mice/group). (**O-R**) Mechanistic requirements for synergistic efficacy. BALB/c WT, C57BL/6 WT, C57BL/6 TLR4^-/-^ KO, and C57BL/6 TLR5^-/-^ KO mice were s.c. implanted with CT26 or MC38 cells. When tumor volume reached ~ 100-120 mm³, mice received CNC018 (2x10^ 7^ CFU/mouse, i.v. injection, day 0) and an αPD-L1 antibody and/or neutralizing antibodies (αHMGB1, αIFN-γ). (**O**) Average CT26 tumor growth curves in BALB/c mice treated with CNC018 + anti-PD-L1 immunotherapy after HMGB1 neutralization (*n* = 3-5 mice/group). (**P**) Average MC38 tumor growth curves in mice treated with CNC018 + anti-PD-L1 immunotherapy in C57BL/6 mice (WT and TLR4^-/-^ KO) (*n* = 5 mice/group). (**Q**) Average MC38 tumor growth curves in mice treated with CNC018 + anti-PD-L1 immunotherapy in C57BL/6 mice (WT and TLR5^-/-^ KO) (*n* = 5 mice/group). (**R**) Average CT26 tumor growth curves in BALB/c mice treated with CNC018 + anti-PD-L1 immunotherapy after IFN-γ neutralization (*n* = 3 - 4 mice/group). *P*-values in J and L-R were determined using two-way ANOVA with Tukey’s multiple comparisons test; ns, not significant.

Building on the scRNA-seq findings (Figure S9), which revealed that CNC018 reprograms the T-cell compartment into an activated, yet exhausted state primed for reinvigoration, we examined the temporal expression profiles of immune checkpoint molecules in CT26 tumor-bearing BALB/c mice after CNC018 treatment using flow cytometry. Basal PD-L1 expression was first examined across four cultured tumor cell lines (CT26, MC38, 4T1, and B16F10) *in vitro* (Figure S16). CNC018 treatment led to a significant upregulation of PD-L1 on both tumor cells (days 4 and 7 post-injection) and on dendritic cells (day 7) within the CT26 tumor (Figure 5A-B), suggesting that CNC018 may modulate the TME to enhance responsiveness to anti-PD-L1 therapy. We further found that CNC018 induced significant upregulation of CTLA-4 expression on total CD4^+^ T cells (CD3^+^CD4^+^) within tumors and regulatory T cells (Tregs; CD3^+^CD4^+^CD25^+^Foxp3^+^) in both TdLNs and tumors, compared to the PBS control group at all time points (Figure 5C-E). This suggests that Tregs constitute a key immunosuppressive population that anti-CTLA-4 therapy could effectively deplete or inhibit, thereby enhancing effector T-cell responses against tumors 36. By contrast, PD-1 expression remained low on effector CD4*^+^* T cells (CD3^+^CD4^+^Foxp3^-^) and CD8^+^ T cells (Figure 5F-G), suggesting that CNC018 sustains T cell functionality and may limit the potential synergy with anti-PD-1 therapy. To obtain an unbiased profile of immune checkpoint expression (PD-L1, CTLA-4, and PD-1), we performed scRNA-seq on tumor-infiltrating mononuclear cells isolated from CT26 tumor-bearing mice treated with PBS or CNC018 (see methods). Consistent with flow cytometry results, CNC018 treatment increased mRNA expression of *Cd274* (encoding PD-L1) and *Ctla-4* (encoding CTLA-4), while downregulating *Pdcd1* (encoding PD-1) (Figure 5H). Collectively, these results indicate that CNC018 upregulates PD-L1 and CTLA-4 while downregulating PD-1, thereby priming the TME for synergy with anti-PD-L1 (αPD-L1) and anti-CTLA-4 (αCTLA-4), yet potentially limiting responsiveness to anti-PD-1 (αPD-1) blockade.

To validate these findings, we evaluated the therapeutic efficacy of CNC018 combined with ICBs in the CT26 syngeneic model (Figure 5I). While all monotherapies inhibited tumor growth, CNC018-ICB combinations yielded markedly superior suppression with minimal systemic toxicity, as evidenced by < 10% body weight loss across all groups (Figure 5J, Figure S17A-B). Notably, while monotherapies induced limited (1 of 5 mice (20%) for αCTLA-4) or no CRs, the addition of CNC018 drastically increased CR frequencies by day 90, reaching 80% with αCTLA-4 (4/5), 60% with αPD-L1 (3/5), and 40% with αPD-1 (2/5) (Figure 5K). Collectively, these results demonstrate that CNC018 synergistically potentiates ICB efficacy, most prominently for αPD-L1 and αCTLA-4, consistent with its role in modulating the immune checkpoint landscape (Table S5).

To assess long-term immune protection, CR mice were rechallenged with CT26 cells on day 90. Rechallenged tumors exhibited minimal or no growth until day 114, whereas age-matched naïve control mice developed progressive tumors (Figure 5L, Figure S17C), indicating durable immune memory in cured mice. Depletion of CD8^+^ T cells completely abolished the anti-tumor effects of CNC018 + ICB combinations (Figure 5M), underscoring their essential role in tumor control. Consistently, adoptive transfer of CD3^+^ T cells from CNC018 + αPD-L1-cured donors conferred tumor regression in 4 of 5 (80%) recipient mice by day 22, whereas none of the naïve T-cell recipients showed regression (0/5; 0%) (Figure 5N, Figure S17D). Collectively, these data show that CNC018 acts synergistically with ICB therapy to induce a potent CD8^+^ T-cell-dependent anti-tumor immune response and establish long-lasting, tumor-specific immune memory.

We next investigated the molecular mechanism underlying the synergistic antitumor activity of CNC018 and αPD-L1, focusing on the induction of ICD. HMGB1, a hallmark DAMP released during ICD, acts as an endogenous ligand for TLR4 to initiate pro-inflammatory responses 37-39. To assess its functional necessity, we neutralized extracellular HMGB1, which significantly abrogated the therapeutic efficacy, survival benefit, and protective antitumor immunity of the CNC018 and αPD-L1 combination (Figure 5O, Figure S18). Given that CNC018 treatment specifically upregulates *Tlr4*, but not *Tlr5*, within the TME (Figure S19), we hypothesized that TLR4 is the primary sensor for this bacterial-induced ICD. Indeed, the synergistic antitumor effect was completely lost in TLR4^-/-^ mice, whereas efficacy remained fully intact in WT and TLR5^-/-^ mice (Figure 5P-Q, Figure S20). These data corroborate and refine previous findings that the antitumor activity of *Salmonella*-based immunotherapy is driven primarily by the TLR4 axis rather than flagellin-mediated TLR5 signaling 13. Since the HMGB1-TLR4 axis typically promotes effector cytokine production to sustain immune surveillance 40-42, we examined the requirement for IFN-γ. Systemic neutralization of IFN-g significantly diminished tumor control, confirming its essential role in this therapeutic cascade (Figure 5R, Figure S21). Collectively, these findings support a functional circuit in which CNC018-induced ICD triggers HMGB1-TLR4 signaling to drive IFN-γ-dependent immunity, thereby providing the molecular priming necessary to sensitize tumors to ICB.

### CNC018 potentiates the anti-tumor efficacy of anti-PD-L1 therapy in multiple tumor models

**Figure 6:**
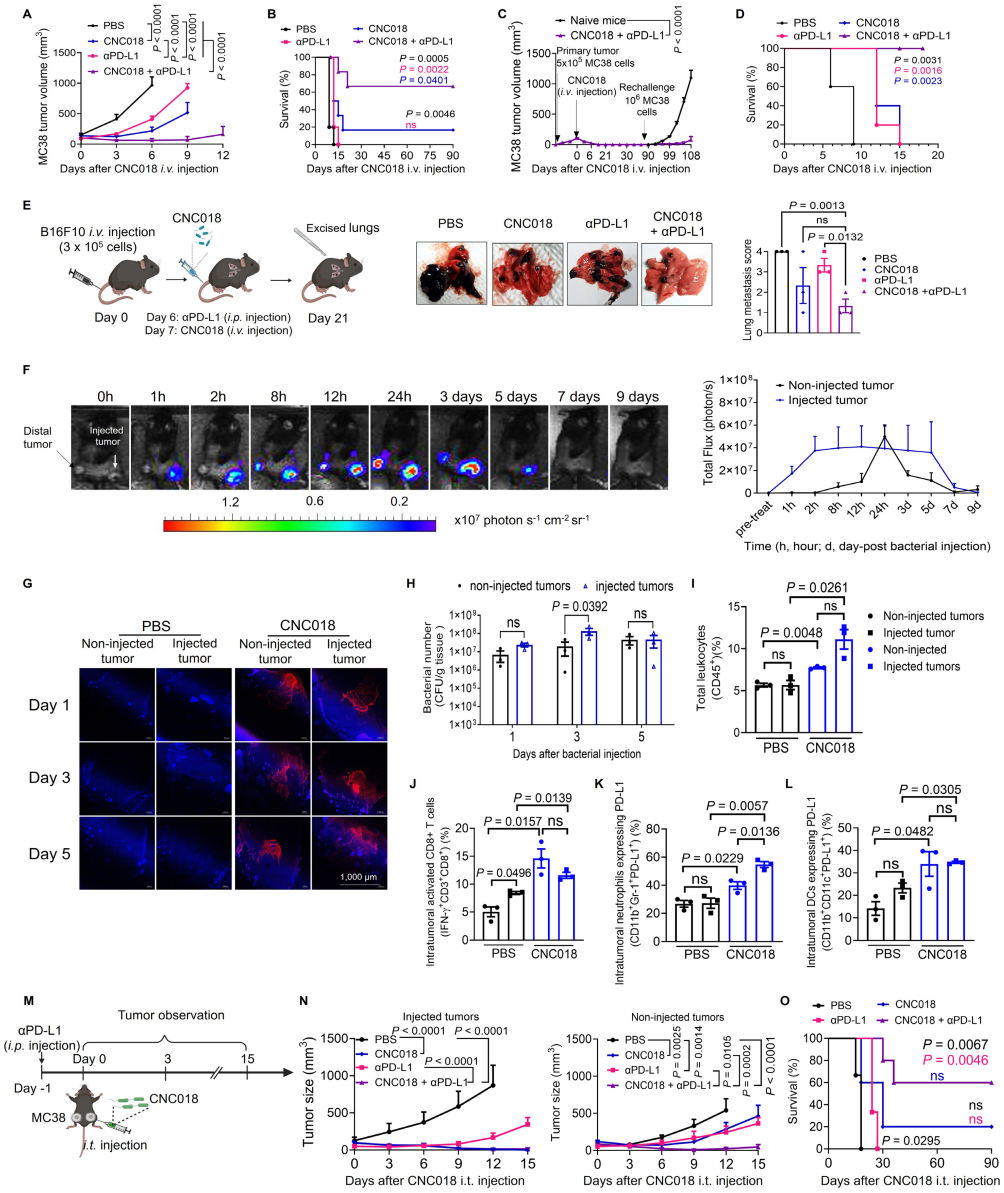
** CNC018 potentiates anti-tumor efficacy of anti-PD-L1 therapy in multiple tumor models.** (**A**) Average MC38 tumor growth curves. C57BL/6 mice were inoculated subcutaneously with MC38 cells. When tumor volume were ~ 100-120 mm³ (day 0), mice were treated with CNC018 (2 × 10⁷ CFU, i.v. injection, day 0) and/or αPD-L1 antibody. Tumor growth was observed (*n* = 5 - 6 mice/group). (**B**) Kaplan-Meier survival analysis of MC38 tumor-bearing mice from (A) [*n* = 5 - 6 mice/group; ns, not significant; log-rank (Mantel-Cox) test]. (**C**) Rechallenge experiment assessing immunological memory. At day 90, cured mice from (B) were *s.c.* reimplanted with MC38 cells (1 × 10⁶) on the opposite flank. Naïve age-matched C57BL/6 mice were inoculated with the same tumor cells (1 x 10⁶) and served as controls (*n* = 3 - 4 mice/group). (**D**) B16F10 survival curves. tumor-bearing C57BL/6 mice. The mice were s.c. implanted with B16F10 cells. Treatments were as described in (A), and survival curves of mice were observed [*n* = 5 mice/group; ns, not significant; log-rank (Mantel-Cox) test]. (**E**) Effect of CNC018 on B16F10 lung metastases. Left, experimental scheme. B16F10 melanoma cells were injected i.v. into C57BL/6 mice (day 0), CNC018 (2 × 10⁷ CFU) was injected i.v. (day 7), and an αPD-L1 antibody (200 µg/mouse, twice-weekly schedule for five doses) was injected i.p. (day 6). Lungs were excised on day 21 for metastasis assessment. Middle, representative lung images. Right, quantification of black metastatic nodules on the lung surface (*n* = 3 mice/group). (**F-H**) Synergistic combination of CNC018 and αPD-L1 in MC38 dual tumor-bearing mice. Bilateral s.c. MC38 inoculation (5 × 10⁵ cells/flank) in C57BL/6 mice. At 150 – 200 mm^3^ bilateral tumor burden, right-side tumors received intratumoral CNC018-*lux* (1× 10^8^ CFU) or CNC018 (1 × 10^8^ CFU) (day 0). αPD-L1 antibody (200 µg/mouse, twice-weekly schedule for five doses) was administered i.p. (day -1). (**F**) Bioluminescence images at the indicated time points of a mouse treated with CNC018-*lux* (left) and quantification of average bioluminescence intensities in injected and non-injected tumors (right) (*n =* 3 mice). (**G**) Immunofluorescence microscopy of tumor sections (day 3): anti-*SL* (red, bacterial detection); DAPI (4′,6-diamidino-2-phenylindole, blue, nuclear counterstain). Scale bar, 1000 μm. (**H**) Viable bacteria were counted in tumors on days 1, 3, and 5 (*n* = 3 mice/group). (**I-L**) Immune cell profiles of non-injected and injected tumors on day 3. (**I**) Frequency of total leukocytes (CD45^+^). (**J**) Frequency of intratumoral activated CD8+ T cells (IFN-γ^+^CD3^+^CD8⁺). (**K**) Frequency of intratumoral neutrophils expressing PD-L1 (PD-L1⁺CD11b^+^Gr-1⁺). (**L**) Frequency of intratumoral DCs expressing PD-L1 (PD-L1^+^CD11b⁺CD11c⁺). *P*-values in E, I-L were determined by an unpaired two-tailed *t*-test (*n* = 3 mice/group; ns, not significant). (**M-O**) Abscopal anti-tumor effect. (**M**) Experimental scheme of CNC018 and αPD-L1 administration in the dual tumor model. (**N**) Average growth curves of CNC018-injected (left) and non-injected (right) tumors in mice treated with PBS, CNC018, αPD-L1, or CNC018 + αPD-L1 (*n* = 3 - 5 mice/group). (**O**) Kaplan-Meier survival curves of MC38 tumor-bearing C57BL/6 mice from (N) [*n* = 5 - 6 mice/group; ns, not significant; log-rank (Mantel-Cox) test]. *P*-values in A, C, H, and N were determined using two-way ANOVA with Tukey’s multiple comparisons test.

Compared to αCTLA-4, αPD-L1 exhibits lower systemic toxicity and therefore provides a more favorable efficacy-to-toxicity profile 43. We thus extended our investigation to assess the anti-tumor efficacy of CNC018 combined with αPD-L1 in additional syngeneic models, including C57BL/6 mice bearing s.c. MC38 colon tumors, s.c. B16F10 melanomas, or B16F10 lung metastases. In the MC38 model, the combination produced the strongest anti-tumor effect, followed by CNC018 monotherapy, αPD-L1 monotherapy, and PBS control, with body weight changes not exceeding 10% in all treatment groups **(**Figure 6A, Figure S22A-B, Table S5). CR was achieved by the combination treatment in 4 of 6 (66.7%) mice by day 20, and all remained tumor-free through day 90 (Figure 6B). Upon MC38 rechallenge, CR mice resisted tumor growth until day 108, whereas age-matched naïve mice developed progressive tumors within 6 days (Figure 6C, Figure S22C). Given the intrinsic resistance of B16F10 melanoma to αPD-L1 therapy 44, we next examined whether CNC018 could enhance therapeutic responsiveness in this model. αPD-L1 monotherapy provided minimal survival benefit, whereas CNC018 + αPD-L1 combination significantly prolonged survival and reduced pulmonary metastatic burden, although no CR mice were observed (Figure 6D-E). To evaluate the translational potential of CNC018, we benchmarked its efficacy against a clinically relevant chemo-immunotherapy regimen comprising 5-fluorouracil (5-FU) and αPD-L1. Notably, the CNC018 + αPD-L1 combination achieved markedly superior tumor suppression and extended survival compared to the 5-FU + αPD-L1 (Figure S23). These results indicate that CNC018-mediated immune priming surpasses the immunostimulatory capacity of conventional chemotherapy, providing a more potent signal to sensitize tumors to ICB. Collectively, these data position this bacterial immunotherapy platform as a compelling strategy to enhance therapeutic outcomes beyond what is achievable with current chemo-immunotherapy standards.

To explore the broader applicability, we investigated whether locally administered CNC018 could affect distant tumors. In dual s.c. MC38 tumor-bearing C57BL/6 mice, CNC018-*lux* was injected intratumorally (i.t.) into one tumor (Figure 6F). Bioluminescence signals appeared within 1 h and persisted for 5 days at the injected site (Figure 6F, left). In the contralateral, non-injected tumor, signals became detectable at 8 h, peaked at 24 h, and remained evident for 4 days (Figure 6F, right). This systemic “homing” was further confirmed by confocal microscopy and quantitative bacterial enumeration, which demonstrated comparable CNC018 colonization densities in both injected and distal tumors (Figure 6G-H). Consistent with the colonization of distal sites, immunophenotyping revealed a synchronized pro-inflammatory shift in both the injected and non-injected tumors compared to PBS-treated controls. This immunological remodeling was characterized by increased frequencies of total leukocytes (CD45^+^), activated effector CD8^+^ T cells (IFN-γ^+^CD3^+^CD8^+^), and PD-L1^+^ innate populations, including neutrophils (PD-L1^+^CD11b^+^Gr-1^+^) and DCs (PD-L1^+^CD11b^+^CD11c^+^) (Figure 6I-L). Collectively, these results demonstrate that unilateral i.t. injection of CNC018 facilitates selective bacterial translocation to distal tumors, effectively priming the TME for enhanced responsiveness to αPD-L1 immunotherapy. To investigate whether localized CNC018 treatment could elicit systemic anti-tumor immunity, we utilized a dual-tumor model in which mice received unilateral i.t. injection of CNC018 combined with i.p. administration of αPD-L1 (Figure 6M). In the injected tumors, both CNC018 alone and the CNC018 + αPD-L1 combination achieved robust and comparable tumor regression, each yielding an 80% (4/5) complete response (CR) rate by day 18 (Figure 6N). In contrast, the synergistic potential of the combination therapy was most evident in the distal, non-injected tumors. While αPD-L1 monotherapy failed to induce any CR, and CNC018 alone showed only limited efficacy (20% CR), the CNC018 + αPD-L1 combination significantly augmented distal tumor suppression, achieving a 60% (3/5) CR rate and markedly prolonging survival (Figure 6O). Safety was maintained throughout the study, with body weight changes remaining within 10% (Figure S24). These results, echoing the systemic efficacy observed with i.v. administration (Figure 5J), demonstrate that local CNC018 treatment effectively primes the systemic immune environment, sensitizing both local and distant tumors to ICB.

## Discussion

**Figure 7:**
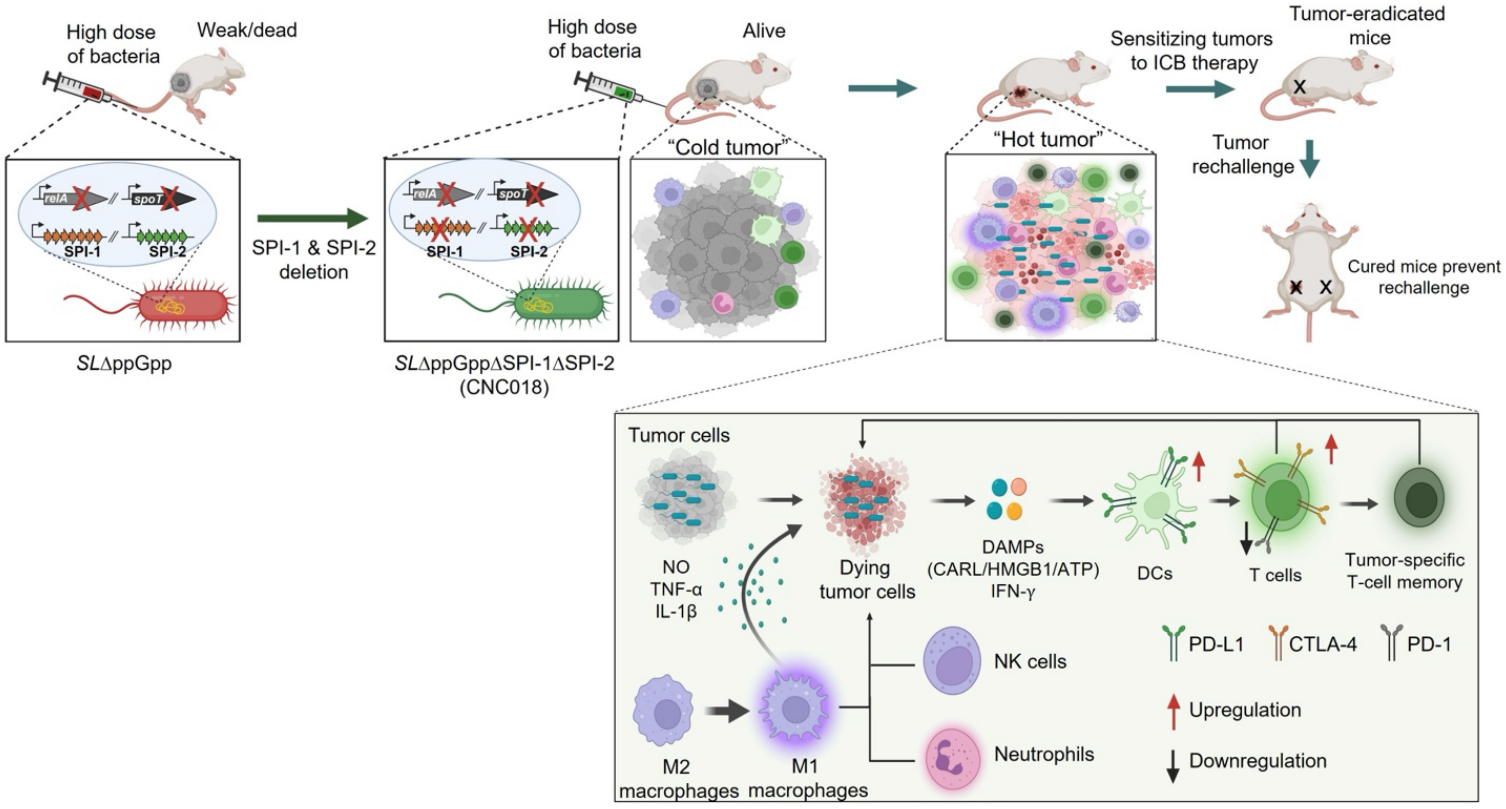
** Clinically translatable, irreversibly attenuated *Salmonella s*train CNC018 reprograms TME to sensitize tumors to ICB immunotherapy.** The graphical abstract illustrates the generation of the *S. typhimurium* strain CNC018 by deleting SPI-1 and SPI-2 from *SL*∆ppGpp, thereby eliminating the risk of virulence restoration and enhancing safety. CNC018 selectively colonizes tumors, triggers ICD and DAMP release, activates anti-tumor immunity, and upregulates immune checkpoints. These changes sensitize tumors to ICB therapy, enabling tumor eradication and the establishment of durable, tumor-specific T-cell memory that prevents tumor rechallenge.

Here, we developed a novel, clinically translatable* Salmonella typhimurium* strain, CNC018, designed to overcome the critical safety limitations of conventional bacterial therapies while enhancing the efficacy of ICB across multiple syngeneic tumor models. Derived from *SL*∆ppGpp through targeted deletion of the SPI-1 and SPI-2 gene clusters, CNC018 retains the potent anti-tumor activity of its parental strain while exhibiting significantly enhanced systemic safety in mice. Building on previous findings that bacteria can potentiate ICB responses 10, 45, we comprehensively investigated how CNC018 remodels the TME and delineated its underlying mechanisms. CNC018 effectively suppressed primary and metastatic tumors by activating innate and adaptive immune responses. Moreover, CNC018-induced DAMPs released from tumor cells promoted DC maturation and expansion of tumor-specific CD8^+^ T cells via the TLR4-NF-κB, JAK-STAT-IRF, and NLRP3 inflammasome signaling pathways. Notably, tumor-colonizing CNC018 modulated the expression of immune checkpoint molecules, thereby priming the TME for synergistic ICB therapy and establishing long-lasting, tumor-specific memory T cells that conferred protection against tumor rechallenge (Figure 7).

The clinical utility of bacterial therapeutics is fundamentally defined by the balance between therapeutic potency and systemic safety. Previous studies have shown that *SL*ΔppGpp achieves substantial attenuation through *relA* and *spoT* deletion; however, its localization of a detectable level (10^3^-10^5^ CFU/g) in non-tumor tissues 13, 39, 46 can limit its clinical translation. Furthermore, the theoretical risk of virulence restoration through horizontal gene transfer and reactivation of SPI-1 and SPI-2 15-17 raises additional safety concerns. CNC018 addresses these limitations through irreversible deletion of 73 genes, including the two primary mediators of ppGpp biosynthesis (*relA* and *spoT*), 38 genes within the SPI-1 cluster, and 33 genes in SPI-2, engineered using the λ Red recombination system. This comprehensive genetic ablation effectively eliminates the risk of virulence reversion while markedly reducing invasiveness and facilitating rapid clearance from non-tumor tissues. This clearance likely results from the heightened susceptibility of CNC018 to phagocytic elimination by neutrophils and inflammatory monocytes 47-50. By integrating tumor-selective colonization with rapid systemic clearance (minimal off-target exposure), metabolic safety, and robust genetic and pharmacological controllability, CNC018 demonstrates exceptional potential as a clinically translatable bacterial platform for intravenous administration. Although the 3.54-fold margin between the LD_50_ and the effective therapeutic dose may represent a relatively narrow window, CNC018 exhibits a superior safety profile without overt systemic toxicity compared to VNP20009, the clinical-grade *S. typhimurium* strain evaluated in phase I clinical trials. Nevertheless, further investigation is needed to define dose-escalation limits and to evaluate therapeutic efficacy in aged mice and comorbidity models that more accurately reflect clinical patient heterogeneity.

A pivotal mechanism underlying the efficacy of CNC018 is its ability to trigger ICD, which serves as a critical bridge between bacterial colonization and adaptive immunity; through this mechanism, we demonstrated that CNC018 effectively reprograms the TME from an immunologically “cold” to a “hot” state, thereby sensitizing tumors to ICB. This transformation is driven by the induction of ICD, as evidenced by the surface exposure of CALR and the release of DAMPs, including ATP and HMGB1. Our findings underscore the functional indispensability of these signals; specifically, the neutralization of CALR or HMGB1 during the early response significantly impaired DC maturation and T-cell activation. Most notably, HMGB1 neutralization completely abrogated the curative potential of the CNC018 + anti-PD-L1 combination, reducing the CR rate from 80% to 0%. Conversely, mice that achieved a CR under the CNC018-based regimen successfully resisted tumor rechallenge, confirming the establishment of a durable, systemic memory response. Collectively, these data indicate that HMGB1-dependent ICD signaling is not merely a transient correlative event but is the essential mechanistic bridge linking initial bacterial sensing to a durable, antigen-specific antitumor response.

Beyond inducing pro-inflammatory cytokines, CNC018 transforms the immune checkpoint landscape by engaging a coordinated signaling network. Specifically, our data demonstrate that CNC018-mediated PD-L1 upregulation is orchestrated by the integration of the canonical JAK-STAT-IRF1 pathway 51-53 with auxiliary innate signals, including the TLR4-NF-κB 54 and NLRP3 inflammasome axes 55. At the core of this circuit is a functional interaction between CNC018-induced HMGB1 and the TLR4 receptor, which triggers a pro-inflammatory milieu that promotes IFN-γ secretion 40-42 to reinforce the JAK-STAT-IRF1 signaling cascade 52, 53. Consistent with these integrated transcriptional profiles shown in Figure 2 and Figure S8, the therapeutic synergy of CNC018 and αPD-L1 was markedly diminished in TLR4^-/-^ mice and upon the systemic neutralization of either HMGB1 or IFN-γ. These results support a mechanistic model in which the HMGB1-TLR4-NF-κB axis acts as a critical catalyst for downstream IFN-γ production, subsequently driving sustained PD-L1 expression via the JAK-STAT-IRF1 pathway 56. By engaging this circuit, CNC018 effectively remodels the TME, rendering tumors highly susceptible to PD-L1 blockade. This immune potentiation is further bolstered by additional hallmarks of CNC018-induced ICD: specifically, surface-exposed CALR and the release of tumor-derived ATP, which activates the NLRP3 inflammasome, provide the essential “eat me” and “danger” signals required for DC maturation and the cross-presentation of tumor-associated antigens to CD8^+^ T cells 39, 57-59. While the precise upstream triggers for these auxiliary pathways merit further investigation, they likely synergize with the HMGB1-TLR4-IFN-γ axis to prime the TME for durable antitumor efficacy via PD-L1-targeted immunotherapy.

Given the requirement of the HMGB1-TLR4-IFN-γ axis for tumor control, bacterial lipopolysaccharide (LPS) serves as a potent exogenous PAMP that facilitates the initiation of this innate cascade 13. As a specific TLR4 agonist, CNC018-derived LPS likely synergizes with host-derived DAMPs to orchestrate a comprehensive activation of both innate and adaptive immunity. While immunosuppressive M2-like macrophages can induce apoptosis of tumor-specific T cells through Fas-FasL signaling 60, CNC018 counteracted this suppression by promoting neutrophil recruitment, M2-to-M1 repolarization, and NK cell activation. Notably, these immunomodulatory effects are sufficient to inhibit both primary and metastatic tumor growth even in T-cell-deficient models, underscoring the intrinsic potency of CNC018-induced innate responses. Furthermore, this robust innate activation provides a critical bridge to adaptive immunity, creating a TME highly conducive to ICB therapy. Future studies to further elucidate the molecular mechanisms regulating macrophage-T cell crosstalk will be essential for optimizing such integrated innate-adaptive therapeutic strategies.

The spatiotemporally distinct modulation of immune checkpoints by CNC018 provides a strategic framework to overcome the inherent resistance of “cold” tumors to ICB. By actively reprogramming the immune landscapes of both the tumor and TdLN, CNC018 addresses the critical constraints of insufficient T-cell activation and dysfunctional antigen presentation that typically limit clinical responses. Within 24 h of treatment, the frequency of CTLA-4-expressing Tregs increased in both tumors and TdLNs, while the population of PD-1⁺CD8⁺ T cells within tumors was markedly reduced. This was followed by the upregulation of PD-L1 on tumor cells by day 4 and on DCs in TdLNs by day 7. These temporal orchestrations indicate that CNC018 elicits a robust T-cell-mediated anti-tumor response that is accompanied by early CTLA-4-dependent regulatory feedback and a delayed IFN-γ-driven PD-L1 adaptive resistance program 61, 62. Simultaneously, CNC018 reshapes the intratumoral T-cell compartment away from a PD-1^high^ exhausted phenotype. Consistent with these mechanistic dynamics, CNC018 monotherapy yielded partial tumor suppression, whereas combination with ICBs targeting CTLA-4 or PD-L1 significantly enhanced therapeutic activity. Specifically, anti-CTLA-4 therapy improved CNC018 efficacy by counteracting the early expansion of CTLA-4^+^ Tregs, while anti-PD-L1 further augmented tumor control by overcoming the late-phase PD-L1-mediated suppressive axis. These findings identify CTLA-4 and PD-L1 as the primary immunoregulatory pathways engaged by CNC018, positioning them as critical targets for therapeutic augmentation.

Furthermore, our scRNA-seq analysis provided deeper insight into this unique immune state; while CNC018-induced T cells exhibited a PD-1^low^ phenotype, they simultaneously upregulated several transcriptomic signatures associated with exhaustion. This apparent paradox suggests that CNC018 reprograms T cells into a distinct, “primed-for-reinvigoration” state, where the molecular machinery for exhaustion is engaged yet the primary inhibitory receptor, PD-1, remains low or is downregulated. This results in a striking immunological asymmetry within the TME: CNC018 induces a profound upregulation of the PD-L1 ligand on tumor cells and DCs, while maintaining a relatively low density of the PD-1 receptor on infiltrating T cells. Such an imbalance, characterized by high ligand availability and low receptor expression, likely explains why the CNC018 + αPD-L1 combination exhibited superior synergistic efficacy compared to αPD-L1 therapy. By specifically targeting the ligand-dominant axis of this asymmetry, CNC018-based immunotherapy provides a strategic molecular priming that maximizes the curative potential of ICB, positioning it as a next-generation platform for overcoming tumor-intrinsic resistance.

The superior synergy observed with αPD-L1 compared to αPD-1 therapy following CNC018 treatment suggests a complex immunomodulatory landscape that extends beyond simple receptor occupancy. While the PD-1^low^ phenotype observed on infiltrating T cells may contribute to the reduced sensitivity to αPD-1, this phenomenon likely integrates multiple biological variables. Notably, CNC018 induced a robust upregulation of PD-L1 on both tumor cells and myeloid populations, including neutrophils and dendritic cells, as shown in Figure 5A-B, and Figure 6K-L. Given that PD-L1-targeted therapies can modulate myeloid-driven suppression and influence antigen presentation 63-66, the broad induction of PD-L1 across these compartments may render the TME particularly vulnerable to PD-L1 blockade. Other factors, such as the spatial compartmentalization of these molecules within the tumor and the potential for Fc-dependent effector functions to enhance the clearance of PD-L1-expressing suppressive cells, cannot be ruled out. While the observed asymmetry between high ligand availability and low receptor expression remains a striking feature of the CNC018-treated TME, further investigation is required to fully elucidate the relative contributions of these diverse mechanisms to the overall therapeutic outcome.

## Conclusions

In summary, we present CNC018 as a rationally engineered *Salmonella typhimurium* platform that reconciles the long-standing conflict between bacterial potency and systemic safety. By integrating large-scale genetic ablation with the induction of immunogenic cell death, CNC018 effectively orchestrates a “cold-to-hot” TME transition, creating a molecular landscape that is uniquely sensitized to immune checkpoint blockade. Our findings demonstrate that CNC018 not only surpasses conventional chemo-immunotherapy in efficacy but also establishes a durable, systemic anti-tumor memory. As a next-generation bacterial adjuvant with a robust safety profile and clear mechanistic advantages, CNC018 holds extraordinary translational promise for breaking tumor-intrinsic resistance and redefining the standard of care in clinical oncology.

## Supplementary Material

Supplementary materials and methods, figures and tables.

## Data Availability

The whole-genome sequencing data generated in this study have been deposited in the Figshare database under accession at https://doi.org/10.6084/m9.figshare.28675010. All of the data reported in this study are available from the corresponding authors upon reasonable request.

## Abbreviations

TME: Tumor microenvironment
ICB: immune checkpoint blockade
*SL*: *Salmonella typhimurium*
WT: *SL14028S*
*SL*∆ppGpp: ppGpp-defective *SL*
SPI: *Salmonella* pathogenicity island
SPI-1: *Salmonella* pathogenicity island 1
SPI-2: *Salmonella* pathogenicity island 2
dpi: Days post-injection
ITP: *In vitro* passage
IVP: *In vivo* passage
FBS: Fetal bovine serum
MOI: multiplicity of infection
PDX: Patient-derived xenograft
DAMPs: Damage-associated molecular patterns
scRNA-seq: Single-cell RNA-sequencing
TdLNs: Tumor-draining lymph nodes
CTLA-4: Cytotoxic T-Lymphocyte Antigen 4
αCTLA-4: anti-CTLA-4
PD-L1: Programmed death ligand 1
αPD-L1: anti-PD-L1
PD-1: Programmed cell death 1
αPD-1: anti-PD-1
Tregs: regulatory T cells
DCs: Dendritic cells
NK: Natural killer cells
ICD: Immunogenic cell death
CALR: Calreticulin
anti-CALR: αCALR
ATP: Adenosine triphosphate
HMGB1: High-mobility group box 1
anti-HMGB1: αHMGB1
T3SS: Type III secretion system
RE: reticuloendothelial
PCR: polymerase chain reaction
LB: Luria-Bertani
i.v.: intravenous
i.p.: intraperitoneal
i.t.: intratumoral
BMDCs: Bone marrow-derived DCs
NK: Natural killer
NSG: NOD-SCID IL2Rγnull
CR: Complete tumor regression
IFN-γ: Interferon-γ
IL: Interleukin
TNF-α: Tumor necrosis factor-α
NO: Nitric oxide
CR: Complete response
TLR: Toll-like receptor
NR-κB: Nuclear factor kappa-light-chain-enhancer of activated B cells
Myd88: Myeloid differentiation primary response 88
Irak4: Interleukin-1 Receptor-Associated Kinase 4
Ifng: Interferon-γ receptor
Jak1/2: Janus kinase 1/2
Stat1: Signal transducer and activator of transcription 1
Irf1: Interferon regulatory factor 1
NLRP3: NLR Family Pyrin Domain Containing 3
Casp1: Caspase 1
5-Fluorouracil: 5-FU
LPS: Lipopolysaccharide
PAMPs: Pathogen-related molecular patterns
