# Supplementary material for: A clinically translatable, irreversibly attenuated Salmonella strain as a next-generation adjuvant for checkpoint immunotherapy
Source: Theranostics. 2026 May 29;16(13):7139–59. doi: 10.7150/thno.129698 (PMC13294988; doi:10.7150/thno.129698)
Supplement: Supplementary file 1 — Supplementary materials and methods, figures and tables. [file thnov16p7139s1.pdf]

Supplementary Materials for

# A clinically translatable, irreversibly attenuated *Salmonella* strain as a next-generation adjuvant for checkpoint immunotherapy

Dinh-Huy Nguyen *et al.*

**Corresponding authors:**

Jung-Joon Min (jjmin@jnu.ac.kr) and Yeongjin Hong (yjhong@jnu.ac.kr)

**This PDF file includes:**

Materials and Methods

Figure S1 to S24

Table S1 to S5

**Materials and Methods**

**NO colorimetric assay**

The tumor-derived nitric oxide (NO) levels were measured indirectly by determining nitrite + nitrate concentrations with the Nitric Oxide Colorimetric Assay Kit (Abcam, Cat # ab65328, UK) following the manufacturer's protocol. Three days after bacterial inoculation, tumors were excised, minced, and homogenized on ice in lysis buffer (1 g tissue: 4 mL buffer, w/v) containing a protease-inhibitor cocktail. After a 1-hour incubation on ice, the homogenates were centrifuged at 10,000 × g for 10 min at 4 °C; the resulting supernatants were collected, and protein concentrations were measured with a BCA assay. To improve NO stability, the supernatants were passed through a 10 kDa molecular-weight-cut-off filter (Abcam) and stored at -80 °C. For the assay, each sample and its corresponding standards were incubated with nitrate reductase and its co-factor for 1 h at room temperature to convert nitrate to nitrite. The Griess-reaction reagents were then added, allowing nitrite to form a purple azo-chromophore; the color was allowed to develop for 10 min. Absorbance was read at 540 nm, providing a detection limit of 1 μM based on a linear calibration curve. NO concentrations were finally expressed as μM/mg of total protein.

**Measurement of cytokine levels**

The levels of the pro-inflammatory cytokines IL-4, IL-6, IL-10, IL-1β, TNF-α, and IFN-γ were quantified by enzyme-linked immunosorbent assay (ELISA) using commercial kits (ABclonal Technology, China) according to the manufacturers' protocols.

**Bacterial biodistribution in tumor-bearing mice**

Bioluminescent *SL*ΔppGpp-*lux* and CNC018-*lux* bacteria ($4 \times 10^7$ CFU) in 100 μL of PBS were injected i.v. into CT26 s.c. tumor-bearing mice. Bioluminescence signals were imaged in the whole body on days 1, 3, and 5 after bacterial treatment using an IVIS Lumina system (PerkinElmer, USA). Bioluminescent signals were quantified within regions of interest as maximum photons per second (p/s) units.

To evaluate *in vivo* bacterial distribution, *SL*ΔppGpp ($2 \times 10^7$ CFU) was administrated via tail-vein injection. At the indicated time points (1, 3, and 5 days), tumors and normal organs were

harvested, weighed, and subjected to mechanical homogenization in PBS (3:1 ratio of volume (mL) per tissue gram (g)) using an IKAT10 Basic ULTRA-TURRAX homogenizer (IKA Dispersers, Germany). Tissue homogenates were serially diluted 10-fold in sterile PBS and plated onto LB agar. Following an overnight incubation at 37 °C, the resulting colonies were enumerated. Bacterial burdens were subsequently normalized and reported as CFU per gram of tissue or per milliliter of blood.

**Antibiotic susceptibility profile of CNC018 in tumor-bearing mice**

To investigate the antibiotic-mediated clearance of CNC018, CT26-tumor-bearing mice (tumor volume ~100 - 120 $mm^3$) were intravenously challenged with the bacteria ($2 \times 10^7$ CFU) on day 0. Oral ciprofloxacin therapy was initiated on day 5 with an initial dose of 60 mg/kg, followed by subsequent daily administrations of 100 mg/kg. To determine the rate of bacterial elimination, tumor tissues and major host organs were harvested on days 4, 7, 14, and 21 for quantitative colony counting. The effectiveness of the antibiotic regimen was assessed by comparing the residual bacterial burden across these time points.

**DC maturation assay**

Primary bone marrow cells were isolated from the tibiae and femurs of BALB/c mice. These cells were maintained in RPMI-1640 (Gibco-BRL, USA) containing 10% FBS, and 1% penicillin-streptomycin and supplemented with murine recombinant GM-CSF (20 ng/mL) and IL-4 (10 ng/mL) (both from R&D Systems). Fresh cytokine-enriched medium was replenished by half-volume exchange on days 2 and 4. On the sixth day of culture, cells were harvested using mechanical scraping and prepared in PBS. For purification, the cells were labeled with CD11c-conjugated magnetic beads (Miltenyi Biotec, USA) for 30 min, and $CD11c^+$ immature dendritic cells (imDCs) were isolated using a MACS Separator (Miltenyi Biotec, Germany) following the provided guidelines. To generate mature DCs (mDCs), the imDCs were further incubated at 37 °C for an additional 48 h.

To evaluate DC activation, CT26 cells ($5 \times 10^5$) were first cultured overnight in 6-well plates using 10% FBS-DMEM and then exposed to CNC018 ($5 \times 10^5$ CFU) for 18 h. Subsequently, either the CNC018-primed CT26 cells ($4 \times 10^5$) or their conditioned media were retrieved and co-incubated with imDCs ($2 \times 10^5$) for 24 h. In neutralizing experiments, CNC018-treated CT26

cells were pre-incubated with 10 μg/mL of anti-HMGB1 or anti-CALR antibodies (Abcam, UK) for 5 h before being introduced to the imDCs. The phenotypic maturation of DCs was subsequently characterized through flow cytometric analysis of surface marker expression.

**Treatment of neutralizing antibody in tumor-bearing mice**

For the systemic depletion of $CD8^+$ T cells, mice were administered 200 μg of an anti-mouse CD8 monoclonal antibody (Bio X Cell, USA) via intraperitoneal (i.p.) injection. This regimen (approximately 10 mg/kg) commenced 24 h prior to bacterial inoculation and was maintained twice weekly for a total of five doses throughout the experimental timeline. For DAMP sequestration studies, either a rabbit anti-HMGB1 antibody (20 μg/mouse; Arigo, Taiwan) or a rabbit anti-CALR antibody (25 μg/mouse; Abcam, UK), diluted in 200 μL of PBS, was delivered i.p. following the same administration frequency and timing established for the T-cell depletion groups.

**Characterization of tumor-associated immune populations**

At 3 or 6 days post-bacterial administration, solid tumor masses, spleens, and TdLNs were excised and placed in 2 mL of chilled isolation medium (RPMI 1640 containing 5% FBS, 1% L-glutamine, 1% penicillin-streptomycin, and 10 mM HEPES). Following mechanical dissociation, tissue fragments were enzymatically digested using 1 mg/mL collagenase type IV and 50 μg/mL DNase I (both from Roche, Switzerland) for 20 - 30 min at 37 °C. Erythrocytes were eliminated by adding an equal volume of 1× RBC lysis buffer (BioLegend, USA) to the 2-mL cell suspension for 4 min at 37 °C. The resulting cell suspensions were passed through 100-μm and then 40-μm mesh filters (BD Falcon, USA). Before antibody labeling, cells were treated with Fc receptor blocking buffer (BioLegend, USA) for 10 min at room temperature. The leukocytes were then incubated on ice for 30 min with fluorophore-conjugated antibodies (Table S3) and quantified using flow cytometry.

To identify tumor-antigen-specific $CD8^+$ T cells, TdLNs were collected on day 6 from mice in the PBS, CNC018, and *SL*ΔppGpp treatment groups. These cells were simultaneously stained with an APC-labeled H-2Ld MuLV gp70 tetramer (SPSYVYHQF; MBL, USA) and a PE-labeled anti-CD8 monoclonal antibody (Ebioscience, USA) before flow cytometric evaluation.

## Supplementary Information

## Supplementary Figures

**Figure S1.** *In vivo* invasion of tumor-colonizing bacteria in CT26 tumors measured by gentamicin-protection assay.

**Figure S2.** Genetic stability and safety of CNC018 during serial passaging.

**Figure S3.** *In vivo* characterizations of CNC018.

**Figure S4.** Systemic toxicity of CNC018.

**Figure S5**. Systemic safety and long-term tolerability of CNC018.

**Figure S6.** Biosafety and antitumor effects of VNP20009 and CNC018.

**Figure S7.** Gating strategies of flow cytometry analysis.

**Figure S8**. CNC018-induced pro-inflammatory responses are mediated by the stimulation of multiple signaling pathways in the TME.

**Figure S9**. ScRNA-seq analysis of marker and cytokine transcript expression in $CD3^+$ T cells infiltrating tumors.

**Figure S10.** DAMPs released from CNC018-induced dying tumor cells.

**Figure S11.** Photos of mice with CT26 tumors taken before and after treatment with CNC018 and anti-DAMP antibodies.

**Figure S12.** Generation of colon cancer PDX tumor model.

**Figure S13.** Tumor size and flow cytometry analysis of PDX tumors treated with CNC018.

**Figure S14.** CNC018 suppresses growth of primary and metastatic murine tumors.

**Figure S15.** CNC018 inhibits liver metastasis.

**Figure S16.** PD-L1 expression on cancer cell lines (CT26, MC38, 4T1, B16F10) *in vitro.*

**Figure S17.** CNC018 strongly potentiates the anti-tumor efficacy of ICB against CT26 tumors.

**Figure S18.** HMGB1 is required for the combination of CNC018 and αPD-L1 antibody to suppress tumors.

**Figure S19.** ScRNA-seq analysis of TLR4 and TLR5 mRNA expression in total cells from CT26 tumors.

**Figure S20.** Antitumor effect of CNC018 combined with anti-PD-L1 immunotherapy on tumor growth in knockout mice.

**Figure S21**. IFN-γ is required for the combination of CNC018 and αPD-L1 antibody to suppress tumors.

**Figure S22.** Tumor suppression by the synergistic combination of CNC018 and αPD-L1 in MC38 tumor-bearing mice.

**Figure S23.** Antitumor effects of 5-FU and CNC018 strain in combination with anti-PD-L1 immunotherapy.

**Figure S24.** Tumor suppression by the synergistic combination of CNC018 and αPD-L1 in MC38 dual tumor-bearing mice treated by intratumoral injection.

**Supplementary tables**

**Table S1.** Strains and plasmids are used in this study.

**Table S2.** Oligonucleotide primers of PCR amplification for bacterial genotyping.

**Table S3.** Antibodies used in this study.

**Table S4.** Xenograft pathological subtype changes of patient colon cancer.

**Table S5.** CDI value of CNC018 plus ICB blockade in CT26 and MC38 tumor models.

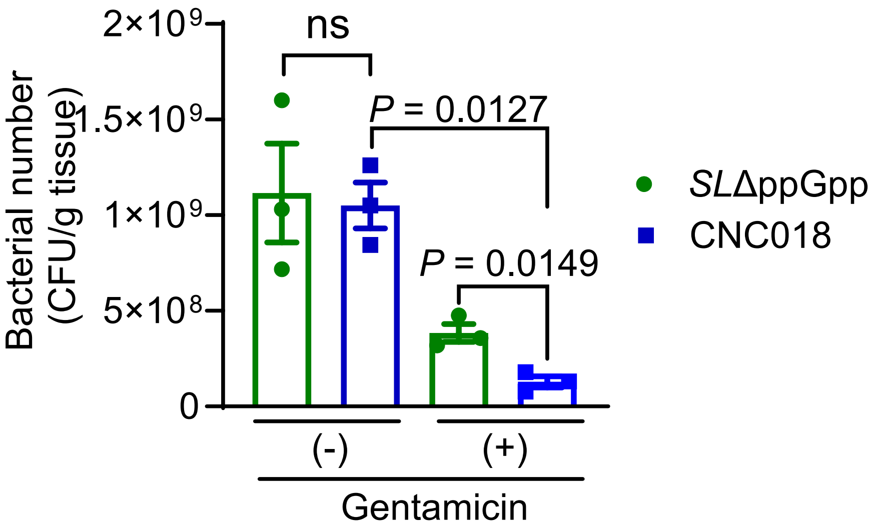


**Figure S1. *In vivo* invasion of tumor-colonizing bacteria in CT26 tumors measured by gentamicin-protection assay** (related to Figure 1E). BALB/c mice carrying CT26 tumors (volume ~120 $mm^3$) were intravenously administered either *SL*ΔppGpp or CNC018 ($2 \times 10^7$ CFU) on day 0. Tumors were excised, weighed, and homogenized on day 3, and bacterial burden was quantified as CFU/ gram of tumor tissue (CFU/g). For the invasion (intracellular) fraction, matched homogenate aliquots were incubated with gentamicin (300 μg/mL) for 1 - 2 h at 37 °C (Gentamicin +) to eliminate extracellular bacteria; untreated aliquots (Gentamicin -) represent total recoverable bacteria ($n$ = 3 mice/group; ns, not significant; unpaired two-tailed $t$-test).

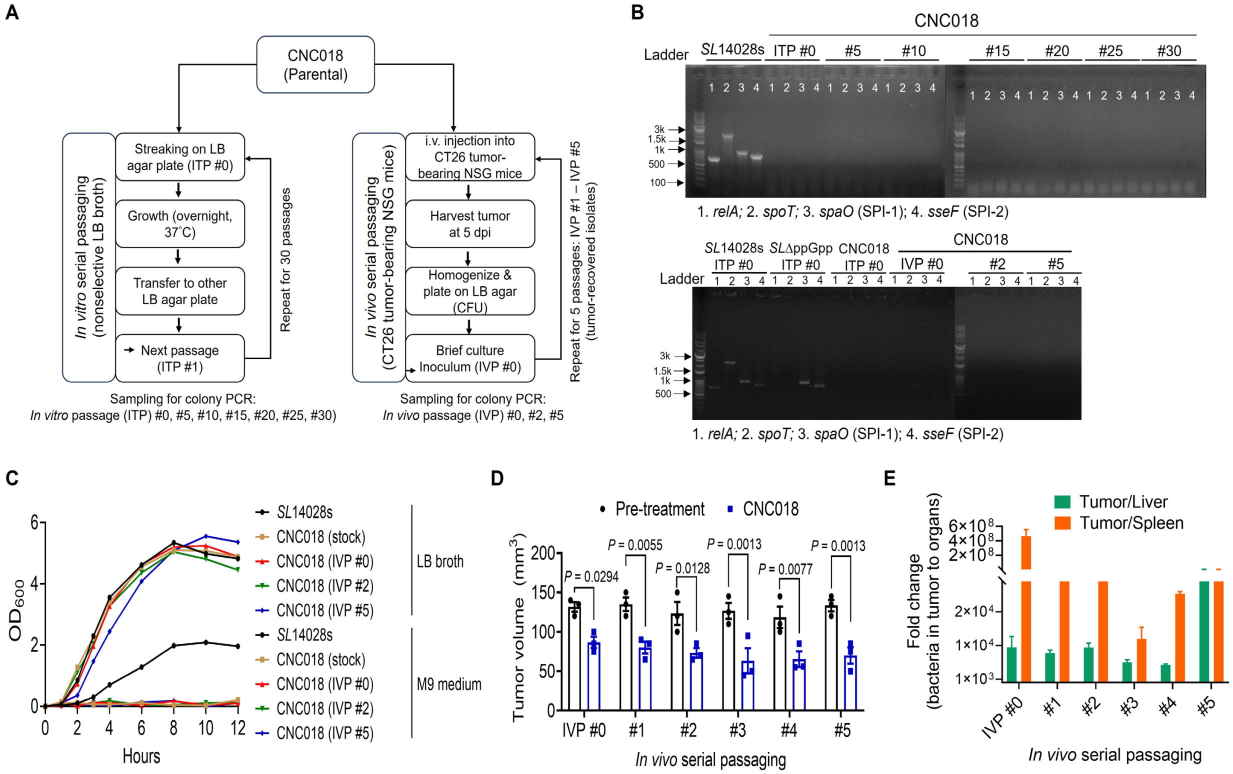


F

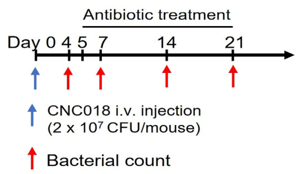


| Antibiotics | MIC (ug/mL) | | | | |
|---|---|---|---|---|---|
| | Pre-injection | 4 dpi | 7 dpi | 15 dpi | 21 dpi |
| Ciprofloxacin | 0.02 | 0.03 | 0.02 | 0.02 | 0.02 |
| Amoxicillin | 0.35 | 0.37 | 0.39 | 0.40 | 0.25 |
| Ceftriaxone | 0.03 | 0.03 | 0.03 | 0.02 | 0.02 |
| Cefixime | 0.07 | 0.07 | 0.06 | 0.06 | 0.04 |

**Figure S2. Genetic stability and safety of CNC018 during serial passaging.** (**A**) Schematic of the stability study design (CNC018 serial passaging). To evaluate potential virulence reactivation under maximal selective pressure, CNC018 was subjected to 30 passages in nonselective LB broth (*in vitro*) and five consecutive passages in CT26 tumor-bearing NSG mice (*in vivo*). For *in vivo* passaging, bacteria were isolated from harvested tumors at 5-day post-injection (dpi), cultured briefly, and used as the inoculum for the subsequent round of tumor-bearing NSG mice. (**B**) PCR genotyping of the engineered deletions. Colony PCR from *in vitro* cultures (*in vitro* passage, ITP #0, #5, #10, #15, #20, #25, #30) (top) and intratumoral isolates recovered from each of the five passages (*in vivo* passage, IVP #0, #2, #5) (bottom). PCR amplicons for regions within *relA*, *spoT*, *spaO* (SPI-1), and *sseF* (SPI-2) were absent across all samples, confirming the permanent maintenance of the attenuation-associated deletions. WT14028S and *SL*ΔppGpp were used as a positive control. (**C**) Preservation of auxotrophy and metabolic attenuation. Growth curves of the original CNC018 and the tumor-recovered isolate (IVP #0, #2, #5) were compared in nutrient-rich LB broth and M9 minimal medium. Both strains showed identical growth kinetics in LB and remained unable to proliferate in M9, demonstrating that the metabolic requirements for attenuation were not bypassed by compensatory mutations. (**D**) Antitumor activity from each of the five recovered isolates (*n* = 3 mice/passage; two-way ANOVA with Tukey's multiple comparisons test). (**E**)

Tumor-preferential colonization. Fold change of bacterial burden in tumors relative to the liver or spleen across the five *in vivo* passages (*n* = 3 mice/passage). (**F**) Antibiotic susceptibility profile. CT26 tumor-bearing mice received CNC018 (2 x $10^7$ CFU) via i.v. injection when the tumor reached approximately 100-120 $mm^3$ in volume (day 0). Antibiotic treatment (ciprofloxacin, amoxicillin, ceftriaxone, and cefixime) was started on day 5 (60 mg/kg), followed by daily oral dosing of 100 mg/kg. The tumors were collected for bacterial count on days 4, 7, 14, and 21 (*n* = 3 mice/time point) to evaluate antibiotic-dependent clearance of CNC018 in tumor-bearing mice. Left: Experimental scheme; right: Minimum Inhibitory Concentrations (MICs) for clinical antibiotics remain unchanged from pre-treatment through 21 dpi, confirming that CNC018 did not acquire antibiotic resistance during prolonged residence in the tumors.

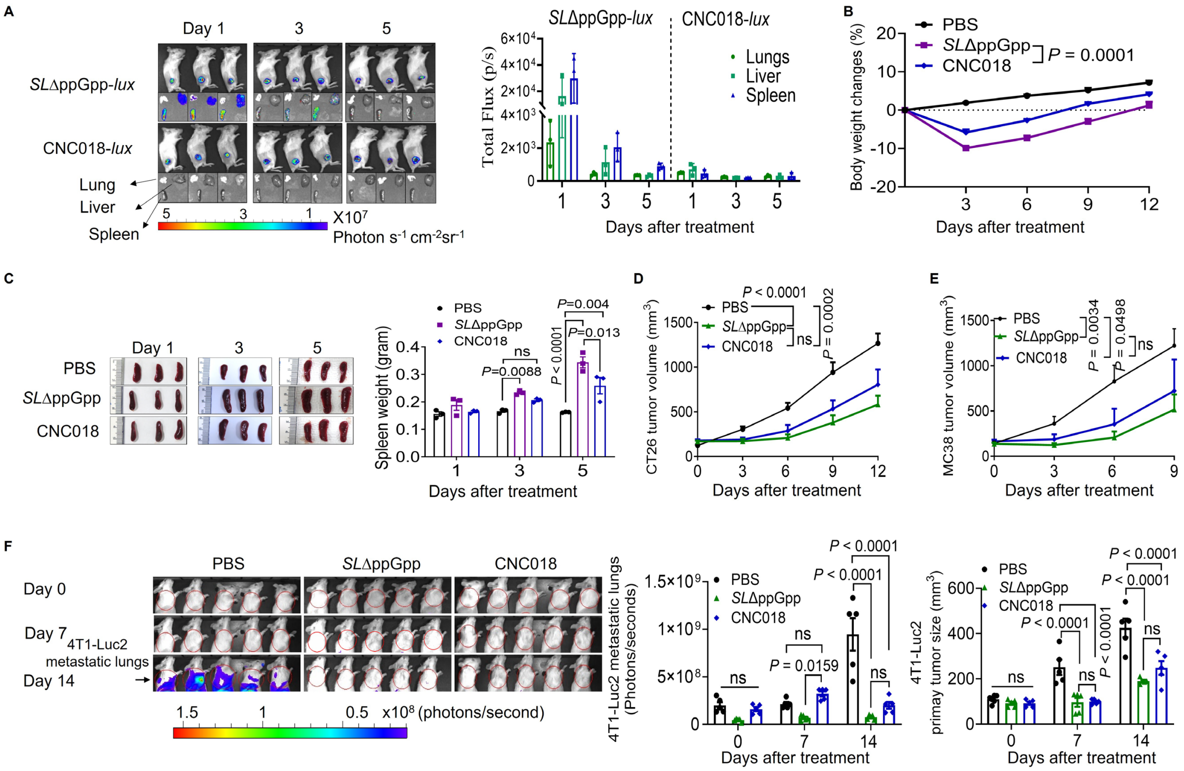


**Figure S3. *In vivo* characterizations of CNC018** (related to Figure 1). CT26, MC38, or 4T1-Luc2 tumor cells were implanted s.c. into mice. When tumor volume reached approximately 120 $mm^3$ (day 0), mice received CNC018 via i.v. injection. (**A**) Tumor-colonizing bacteria in mice. *SL*ΔppGpp-*lux* or CNC018-*lux* ($4 \times 10^7$ CFU/mouse), bioluminescent strains transformed with the *lux* operon, were injected i.v. into BALB/c mice bearing CT26 tumors ($n$ = 3 mice/group). Luminescence images of whole mice and normal tissues (lungs, liver, and spleen) were acquired on days 1, 3, and 5 post-bacterial injections. Left, representative images; right, quantification of bioluminescence signals in normal organs. (**B**) Body weight changes of mice treated with PBS, *SL*ΔppGpp, or CNC018 ($2 \times 10^7$ CFU/mouse) ($n$ = 7 mice/group, two independent experiments). (**C**) Splenomegaly of CT26 tumor-bearing mice after bacterial treatment. Left, *ex vivo* spleen images; right, spleen weights ($n$ = 3 mice/group). **(D, E)** Comparative tumor progression kinetics in **(D)** CT26-bearing BALB/c mice and **(E)** MC38-bearing C57BL/6 mice ($n$ = 7 per group, two independent experiments). **(F)** Assessment of 4T1-Luc2 metastatic burden and primary tumor growth. On days 0, 7, and 14 post-treatment, mice received D-luciferin (150 mg/kg, i.p.) for bioluminescence visualization. Representative images (left), quantified luciferase activity of pulmonary metastases (middle), and primary tumor volumes (right) are displayed ($n$ = 5 mice/group). Data in B-F were analyzed via two-way ANOVA followed by Tukey's multiple comparisons test; ns, not significant.

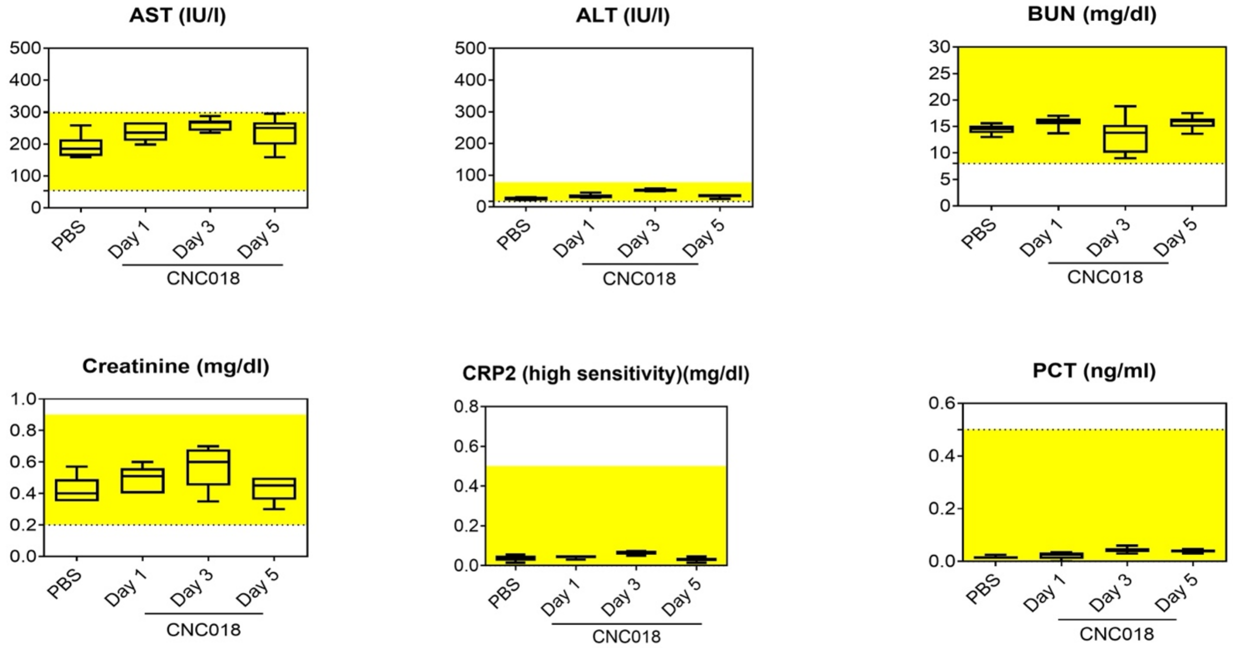


**Figure S4. Systemic toxicity of CNC018**. BALB/c mice carrying CT26 tumors were intravenously administered CNC018 (2 x $10^7$ CFU per animal; *n* = 7 per group, representative of two independent experimental trials) on day 0. To evaluate organ function and systemic inflammation, serum concentrations of alanine aminotransferase (ALT), aspartate aminotransferase (AST), blood urea nitrogen (BUN), and creatinine, alongside plasma levels of C-reactive protein 2 (CRP2) and procalcitonin (PCT), were quantified at 1, 3, and 5 days post-administration. The yellow-shaded regions represent the standard physiological reference ranges for each respective biomarker.

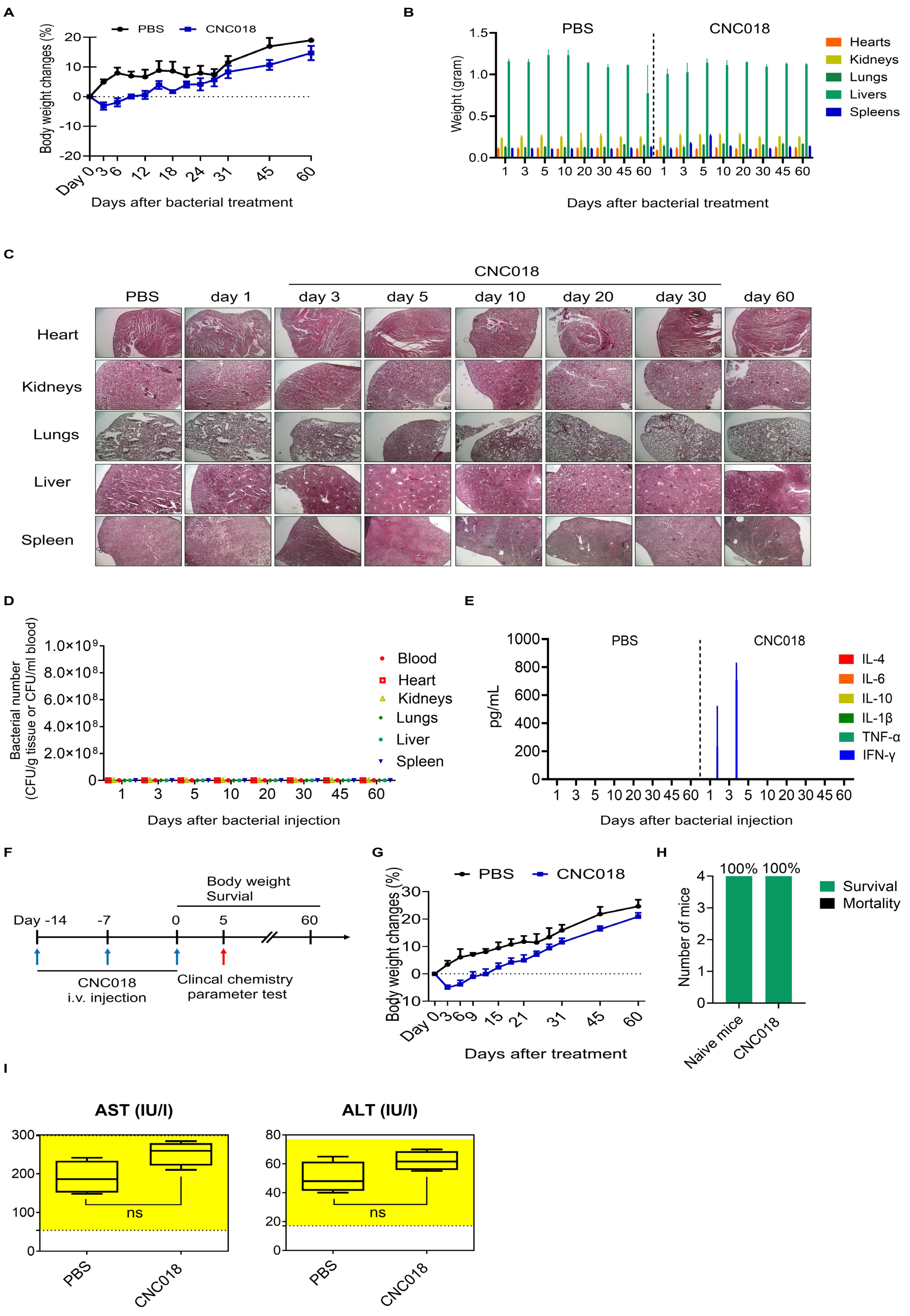
A
PBS
CNC018
Body weight changes (%)
Days after bacterial treatment
B
PBS
CNC018
Hearts
Kidneys
Lungs
Livers
Spleens
Weight (gram)
Days after bacterial treatment
C
CNC018
PBS
day 1
day 3
day 5
day 10
day 20
day 30
day 60
Heart
Kidneys
Lungs
Liver
Spleen
D
Bacterial number (CFU/g tissue or CFU/ml blood)
Blood
Heart
Kidneys
Lungs
Liver
Spleen
Days after bacterial injection
E
PBS
CNC018
pg/mL
IL-4
IL-6
IL-10
IL-1β
TNF-α
IFN-γ
Days after bacterial injection
F
Body weight
Survial
Day -14
-7
0
5
60
CNC018
i.v. injection
Clincal chemistry
parameter test
G
PBS
CNC018
Body weight changes (%)
Days after treatment
H
100%
100%
Survival
Mortality
Number of mice
Naive mice
CNC018
I
AST (IU/l)
ALT (IU/l)
ns
PBS
CNC018

**Figure S5**. **Systemic safety and long-term tolerability of CNC018**. Naïve BALB/c mice were treated with PBS or CNC018 ($2\times10^7$ CFU, i.v. injection) and monitored for 60 days. (**A**) Body weight changes of mice expressed as percent of baseline (Day 0) (*n* = 4 mice/group for PBS, *n* = 5 mice/group for CNC018). (**B**) The weight changes of organs post-treatment (*n* = 3 mice/group). (**C**) Representative H&E images of organs (heart, kidneys, lungs, liver, spleen) were collected on days 1, 3, 5, 10, 20, 30, and 60. (**D**) Bacterial burdens in organs at indicated time points quantified as CFU/g tissue. (**E**) Serum cytokines measured at indicated times post-treatment (*n* = 3 mice/group). (**F - I**) The sustained biosafety of CNC018 in a therapeutic repeat-dosing study for long-term observation. (**F**) Schematic of the triple-dose toxicity study. BALB/c mice (*n* = 4 mice/group) received i.v. injections of CNC018 ($2 \times 10^7$ CFU) or PBS for triple-dose. (**G**) Body weight change during treatment. (**H**) Survival of mice after 60 post-treatments from (F). (**I**) Quantification of serum AST and ALT levels on day 5. Yellow shading indicates normal reference ranges for each parameter. Statistical significance was determined using an unpaired two-tailed *t*-test; ns, not significant.

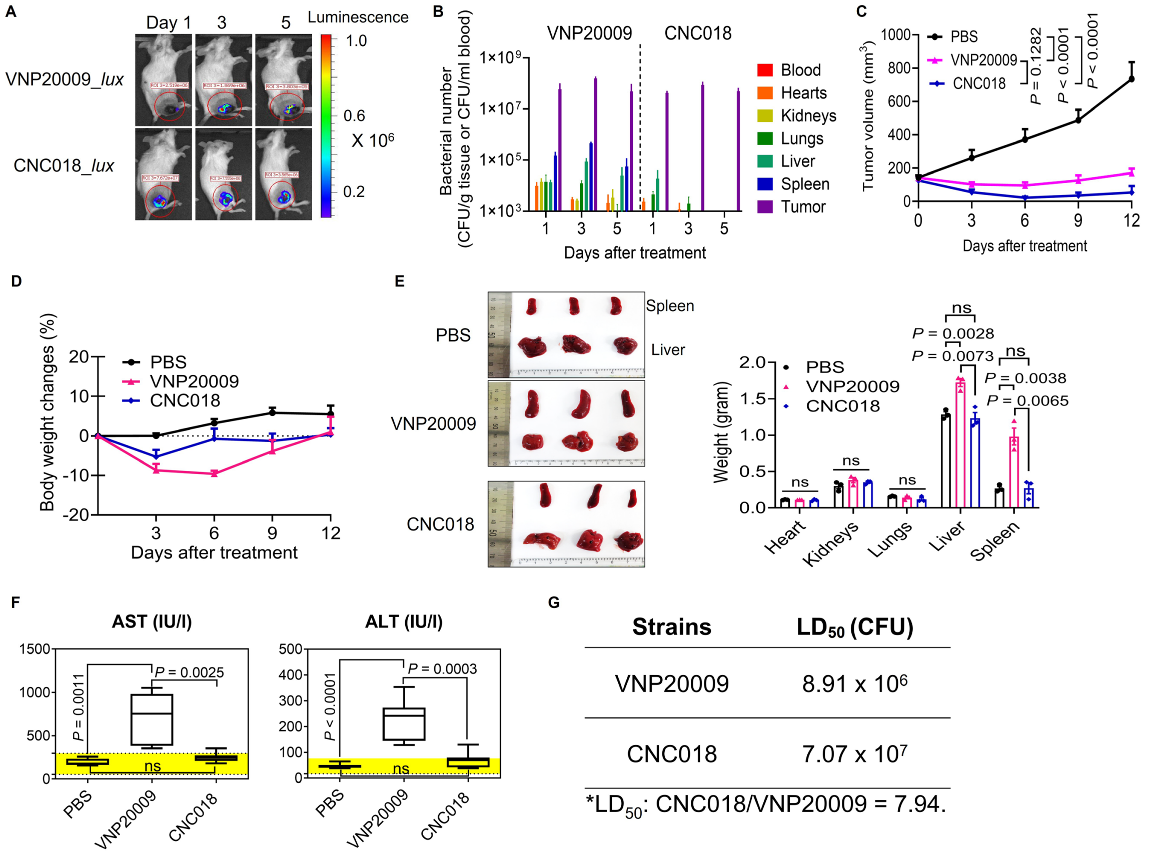


**Figure S6. Biosafety and antitumor effects of VNP20009 and CNC018.** (**A&B**) Tumor-colonizing bacteria in mice. CNC018-*lux*, VNP20009-*lux*, VNP20009, or CNC018 (2 x $10^7$ CFU/mouse) were injected i.v. into BALB/c mice bearing CT26 tumors ($n$ = 3 mice/group) (day 0). (**A**) Luminescence images of mice treated with CNC018-*lux* and VNP20009-*lux* were acquired at 1, 3, and 5 dpi ($n$ = 3 mice/group). (**B**) Bacterial count of CNC018 and VNP20009 at 1, 3, and 5 dpi ($n$ = 3 mice/group). (**C**) Average growth curves of tumors in mice treated with PBS, VNP2009, or CNC018. (**D**) Body weight changes of mice after bacterial treatment. *P*-values in C-D were determined by two-way ANOVA with Tukey's multiple comparisons test ($n$ = 4 mice/group; ns, not significant). (**E**) Organ weight after bacterial treatment on day 3. Left: *ex vivo* spleen and liver images; right: spleen and liver weights ($n$ = 3/group). (**F**) Serum levels of aspartate aminotransferase (AST) and alanine aminotransferase (ALT) were measured on day 3 after treatment. Yellow shading indicates normal reference ranges for each parameter (n = 7 mice/group). *P*-values in E-F were determined by an unpaired two-tailed *t*-test; ns, not significant. (**G**) Median lethal dose ($LD_{50}$) in mice following bacterial infection. BALB/c mice ($n$ = 5 mice/group) were treated with VNP20009 or CNC018 via i.v. injection. Mouse survival was monitored for 30 days to determine the $LD_{50}$ using the Reed and Muench method.

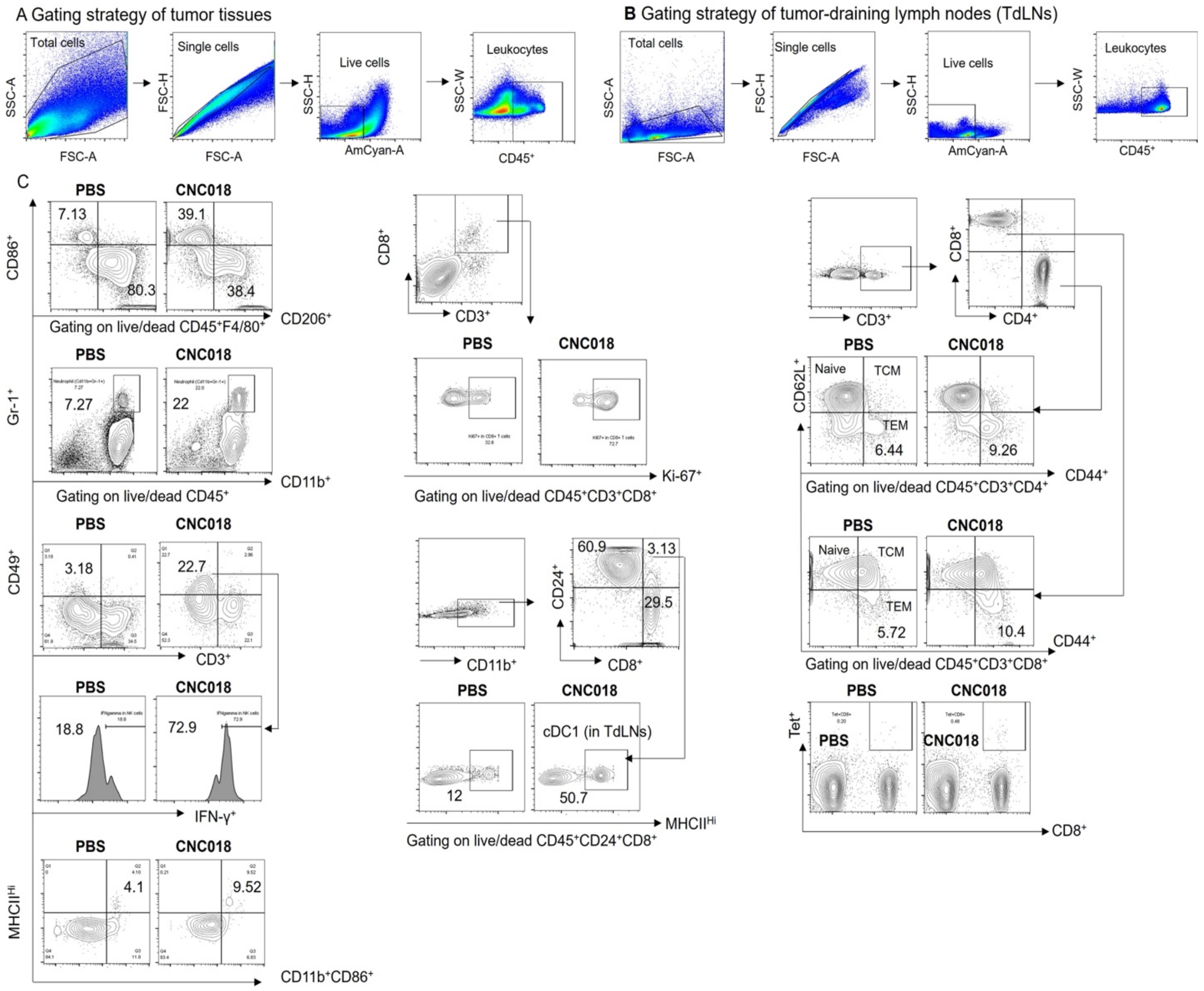


**Figure S7. Gating strategies of flow cytometry analysis** (related to Figure 2). Immune cells were harvested from CT26 tumors and TdLNs. Cells were identified by flow cytometry after staining with a Live/Dead cell kit, anti-CD45 (leukocyte marker), anti-CD16/CD32 (to block non-specific Fc receptor binding), and additional immune cell-specific markers. (**A**) Gating strategy for tumor tissue analysis. (**B**) Gating strategy for TdLN analysis. (**C**) Immune cell profiles determined from gates shown in (A) and (B).

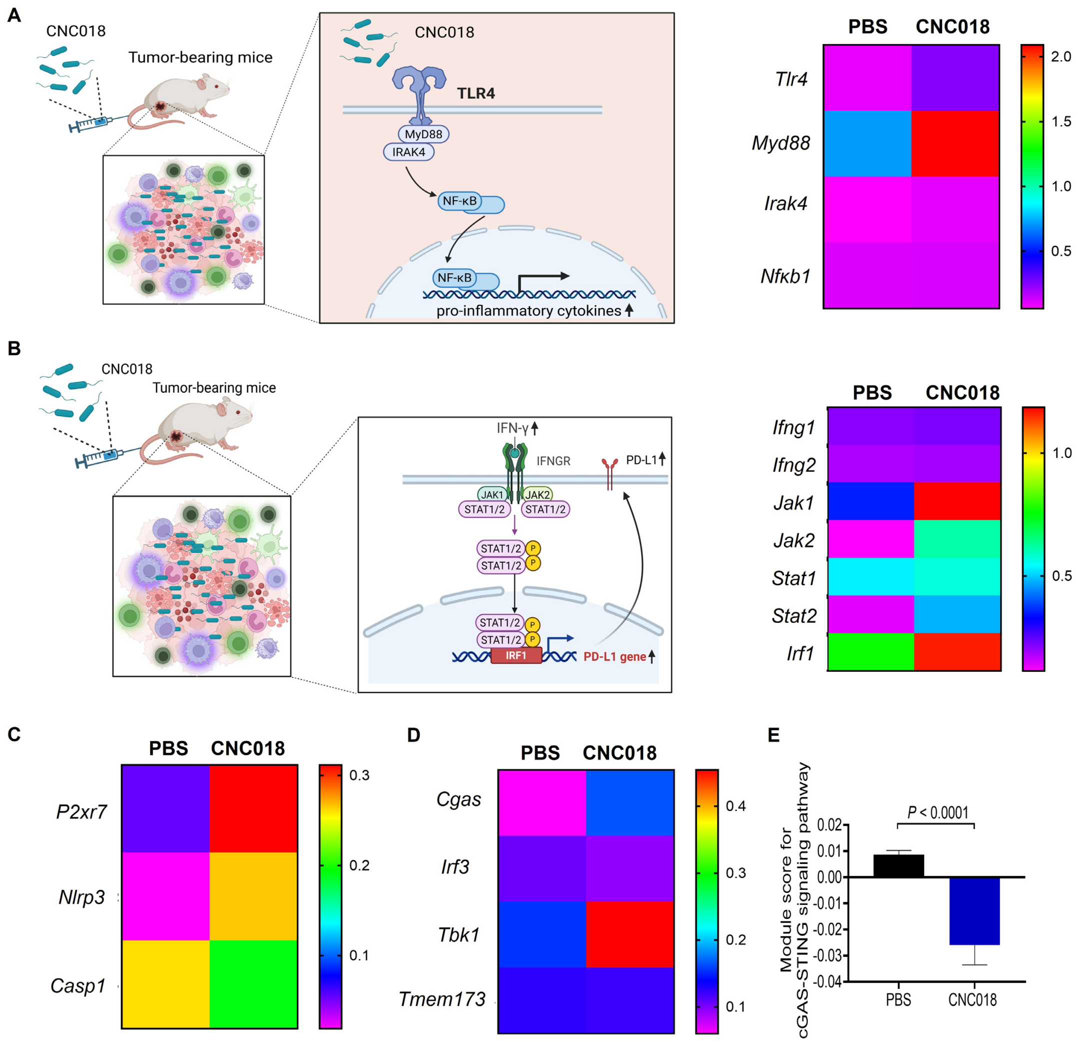


**Figure S8. CNC018-induced pro-inflammatory responses are mediated by the stimulation of multiple signaling pathways in the TME** (related to Figure 2P-R). BALB/c mice carrying CT26 tumors were intravenously challenged with either PBS or CNC018 (2 x $10^7$ CFU) once tumors reached a volume of ~120-140 mm³ (designated as day 0). Tumors were harvested on day 3 for scRNA-seq analysis (*n* = 2 pooled tumors per group). (**A**) Schematic diagram of a signaling pathway relating CNC018-induced pro-inflammatory cytokines (left); heatmaps show representative mRNA gene expression of TLR4 signaling, including 4 genes (*Tlr4, Myd88, Irak4,* and *Nfκb*) (right). (**B**) Schematic diagram of the signaling pathway relating to the CNC018-induced IFN-γ signaling pathway (left); heatmaps show representative mRNA gene expression of IFN-γ signaling, including 7 genes (*Ifng1*, *Ifng2, Jak1*, *Jak1, Stat1*, *Stat2,* and *Irf1*) (right). (**C**) Heatmaps show representative mRNA gene expression of inflammasome activation (*P2xr7,*

*Nlrp3*, and *Casp1*), and (**D**) heatmaps show representative mRNA gene expression of the cGAS–STING signaling pathway (*Cgas*, *Irf3*, *Tbk1*, and *Tmem173*). (**E**) The mRNA levels of signaling module scores from (D) (n = 2 combined tumors for each group; unpaired two-tailed *t*-test).

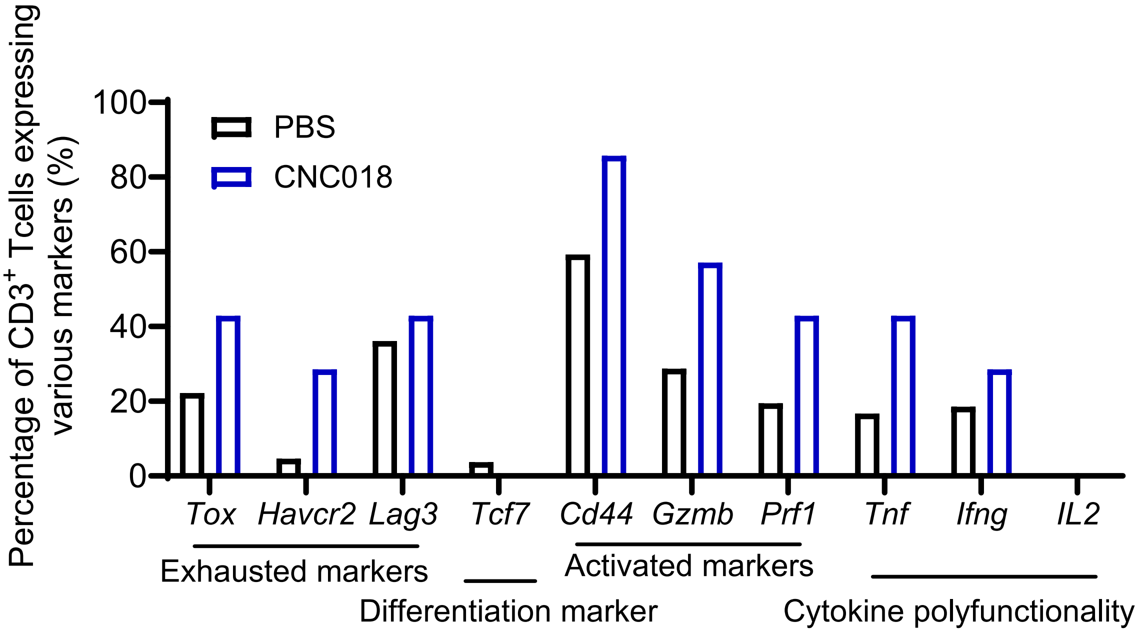


**Figure S9**. **ScRNA-seq analysis of marker and cytokine transcript expression in CD3⁺ T cells infiltrating tumors.** scRNA-seq was performed on CT26 tumors in mice treated with PBS or CNC018 ($n$ = 2 pooled tumors per group) on day 3 after bacterial treatment. Bar plots show the frequencies of $CD3^+$ T cells expressing *Tox* (TOX), *Havcr2* (TIM3), *Lag3* (LAG3), *Tcf7* (TCF-1), *Cd44* (CD44), *Gzmb* (GzmB), *Prf1* (Perforin), *Tnf* (TNF-α), *Ifng* (IFN-γ), and *Il2* (IL-2) mRNA in the PBS and CNC018 groups. The markers are grouped according to exhaustion, differentiation, activation, and cytokine polyfunctionality, as indicated in the figure.

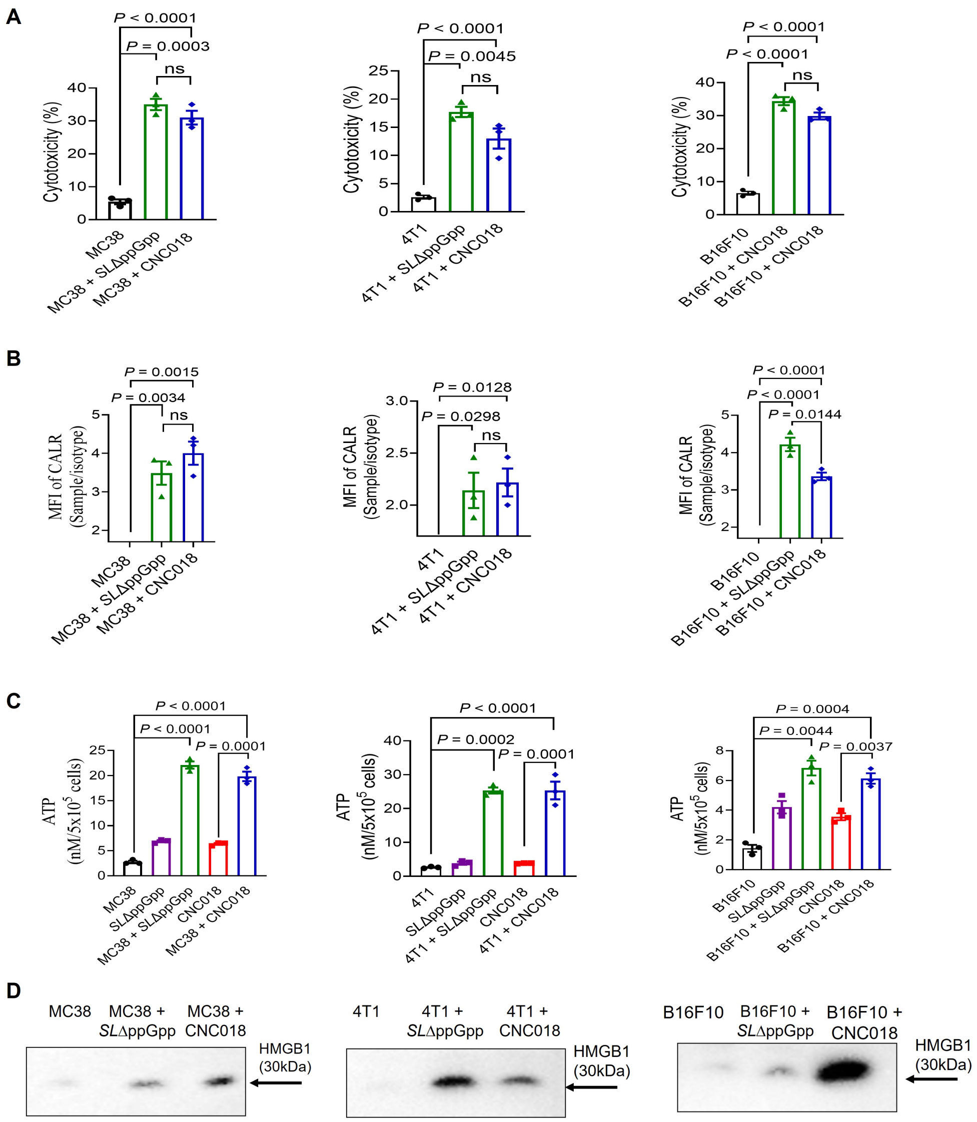


**Figure S10. DAMPs released from CNC018-induced dying tumor cells** (related to Figure 3A-D). CT26, MC38, 4T1, or B16F10 cells (1 x $10^5$) were cultured in antibiotic-free complete medium for 14-16 h, then treated with PBS, *SL*ΔppGpp, or CNC018 (1 x $10^6$ CFU) in antibiotic-free medium for an additional 20-24 h. (**A**) Cytotoxicity assay. Cell viability was performed by evaluating lactate dehydrogenase (LDH) release from the supernatant (*n* = 3 technical replicates). (**B**) CALR exposure on the cell surface. Cells were stained with anti-CALR (αCALR) or IgG control, and mean fluorescence intensity (MFI) was measured by flow cytometry (*n* = 3 technical replicates). (**C**) ATP secretion from cancer cells. ATP levels were quantified in the culture supernatant (*n* = 3 technical replicates). *P*-values in A-C were calculated by an unpaired two-tailed *t*-test; ns, not significant. (**D**) HMGB1 release from cancer cells. Culture supernatants were analyzed by Western blotting using anti-HMGB1 antibody.

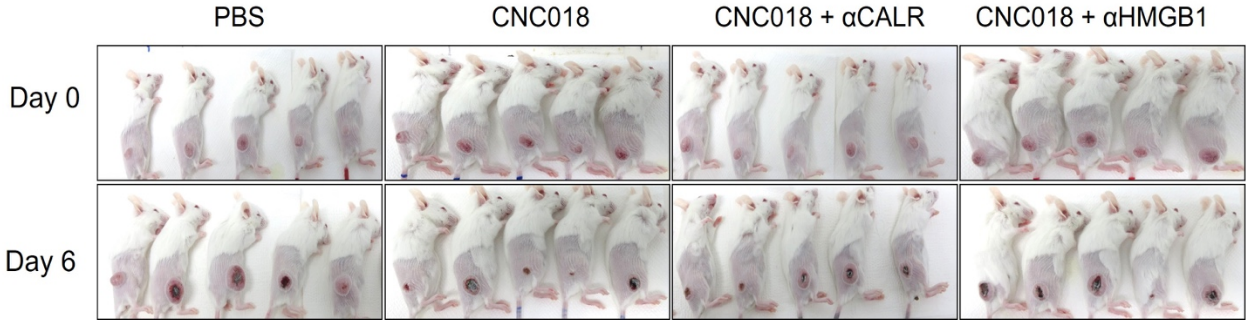


**Figure S11. Photos of mice with CT26 tumors taken before and after treatment with CNC018 and anti-DAMP antibodies** (related to Figure 3F-J). BALB/c mice underwent subcutaneous inoculation with CT26 cells (5 x $10^5$). Upon reaching a mean tumor volume of ~120 mm³, subjects were administered either PBS or CNC018 (2 x $10^7$ CFU) through a single tail-vein injection (day 0). To sequester DAMPs, anti-CALR (αCALR) or anti-HMGB1 (αHMGB1) monoclonal antibodies were delivered intraperitoneally on a biweekly schedule (commencing on day -1) for three consecutive doses. Mouse images on days 0 and 6 are shown for each treatment group: PBS, CNC018, CNC018 + αCALR, and CNC018 + αHMGB1.

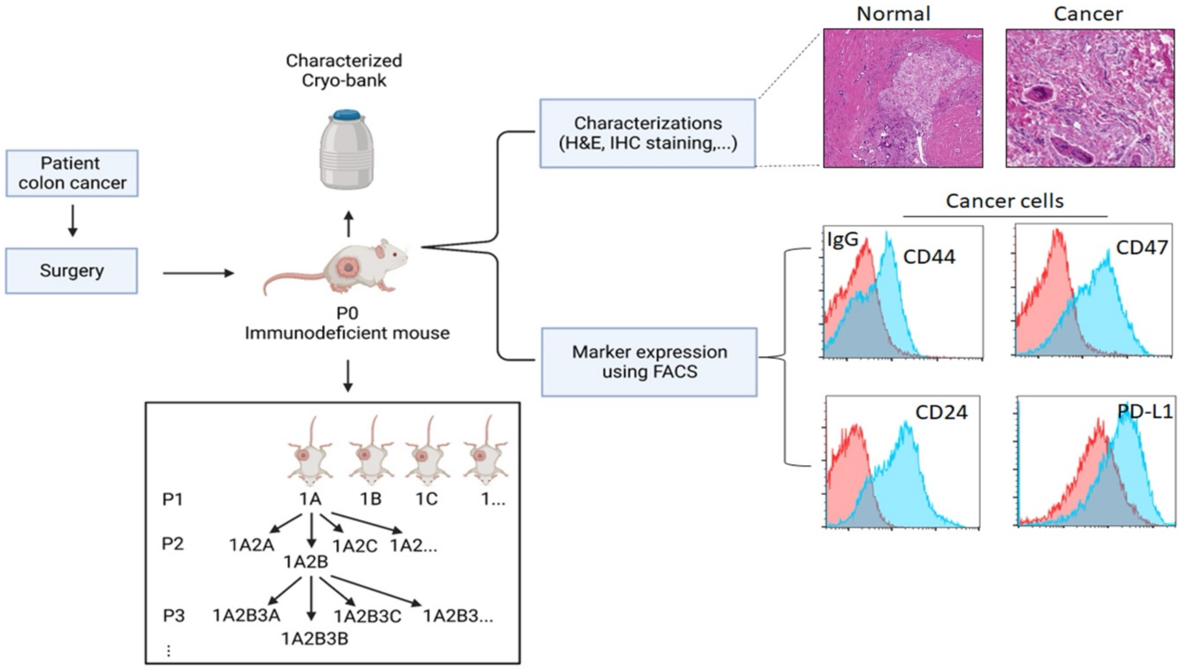


**Figure S12. Generation of colon cancer PDX tumor model** (related to Figure 4A-H). Surgical specimens from colon cancer patients were divided into small pieces and transplanted s.c. into NSG mice (P0). Tumor xenografts from P0 mice were characterized by hematoxylin and eosin (H&E) staining and flow cytometry, stored in cryobanks for subsequent implantation. Xenografting was repeated in NSG mice (P1 and subsequent passages). Final xenograft mice (P3) were used for subsequent bacterial treatment. Representative micrographs show the H&E staining of normal and tumor tissue (top right). Flow cytometry histograms (bottom right) indicate expression of CD44, CD47, CD24, and PD-L1 on tumor cells (blue) versus IgG control (red). See Materials and Methods for details.

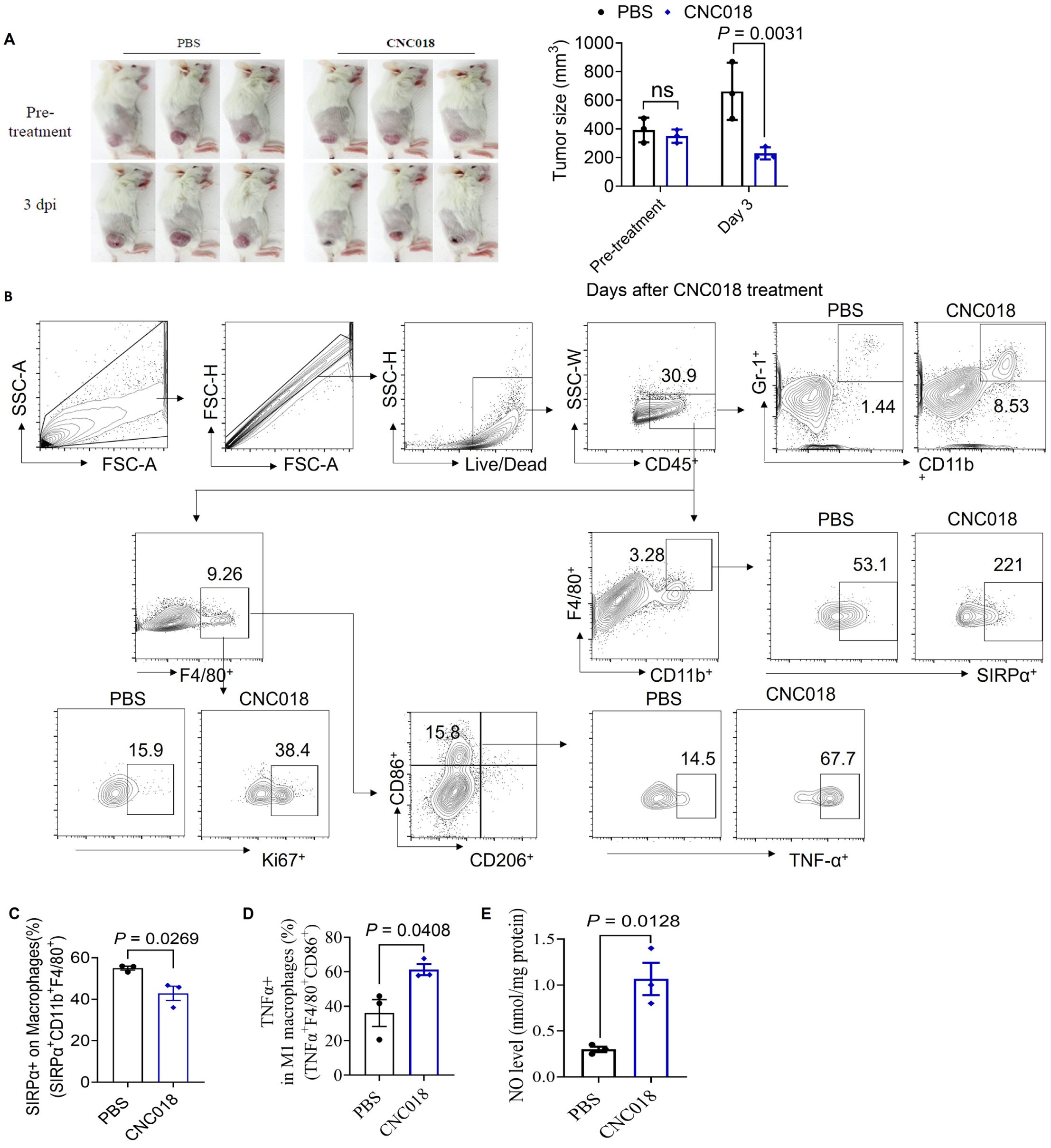


**Figure S13. Tumor size and flow cytometry analysis of PDX tumors treated with CNC018** (related to Figure 4A-H)**.** Tumor cells from colorectal cancer patients ($1 \times 10^6$) were inoculated s.c. into the flank of NSG mice to establish a patient-derived xenograft (PDX) tumor model (P0). After four passages, P3 tumor-bearing mice were injected i.v. with PBS or CNC018 ($2 \times 10^7$ CFU) when tumor size reached approximately 300 mm³ (day 0). Intratumoral immune cells were analyzed by flow cytometry. (**A**) Tumor size at 0 dpi (pre-treatment) and 3 dpi. Left, representative images of mice; right, tumor volumes (*n* = 3 mice/group; ns, not significant; two-way ANOVA with Tukey's multiple comparisons test). (**B**) Flow cytometry gating strategy. (**C**) Proportion of activated macrophages (SIRPα$^+$CD11b$^+$F4/80$^+$). (**D**)

Frequency of activated M1 macrophages ($TNF\alpha^{+}F4/80^{+}CD86^{+}$). (**E**) NO concentrations in tumor lysates. *P*-values in C-E were detected by an unpaired two-tailed *t*-test (*n* = 3 mice/group).

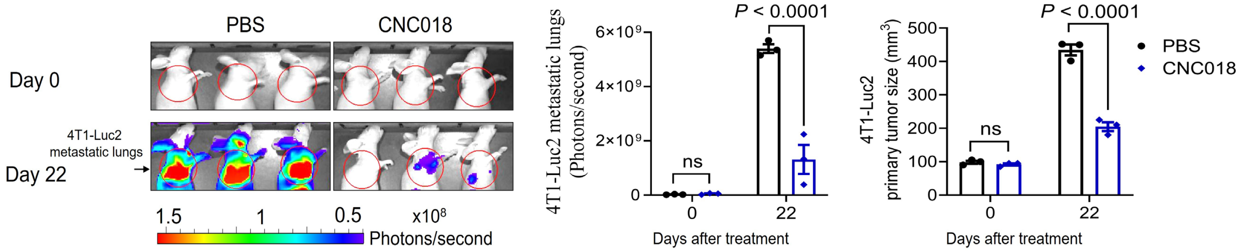


**Figure S14. CNC018 suppresses growth of primary and metastatic murine tumors.** BALB/c athymic *nu/nu* mice were implanted s.c. with 4T1-Luc2 cells ($5 \times 10^5$). When primary tumor volumes reached 120 - 140 mm³, mice received PBS or CNC018 ($2 \times 10^7$ CFU/mouse) via i.v. injection (day 0). *In vivo* bioluminescence imaging was performed after i.p. injection of D-luciferin (150 mg/kg, FLuc activity. Left: Longitudinal macroscopic bioluminescence imaging of representative mice at baseline (day 0) and day 22. Middle: Assessment of metastatic load in the lungs based on bioluminescence signal intensity on days 0 and 22. Right: Statistical comparison of primary tumor volumes on days 0 and 22 ($n$ = 3 mice/group; ns, not significant; analysis via two-way ANOVA with Tukey's multiple comparisons test).

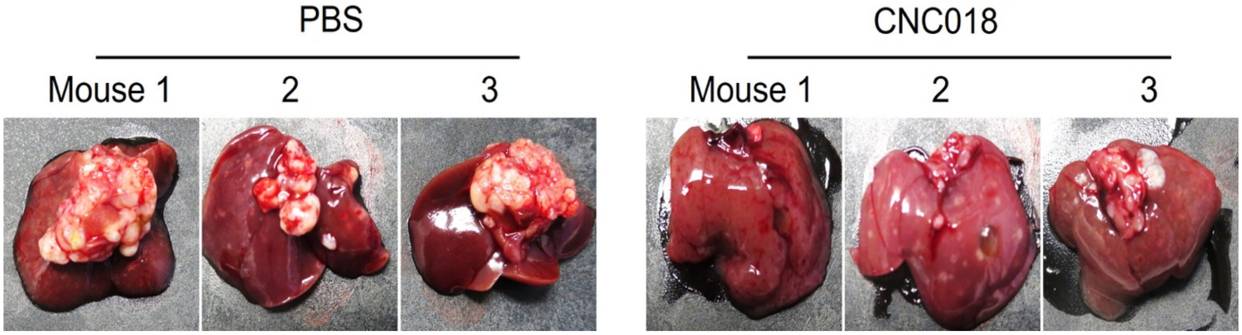


**Figure S15. CNC018 inhibits liver metastasis** (related to Figure 4I). Liver metastases were established by i.p. injection of human hepatoma HepG2-Fluc cells ($3 \times 10^5$) into immunocompromised athymic *nu*$^-$/*nu*$^-$ BALB/c mice. Three days after tumor implantation, CNC018 ($2 \times 10^7$ CFU) was administered via i.v. injection (day 0). Representative *ex vivo* images of livers were harvested at the experimental endpoint (day 29) (n = 3 mice/group).

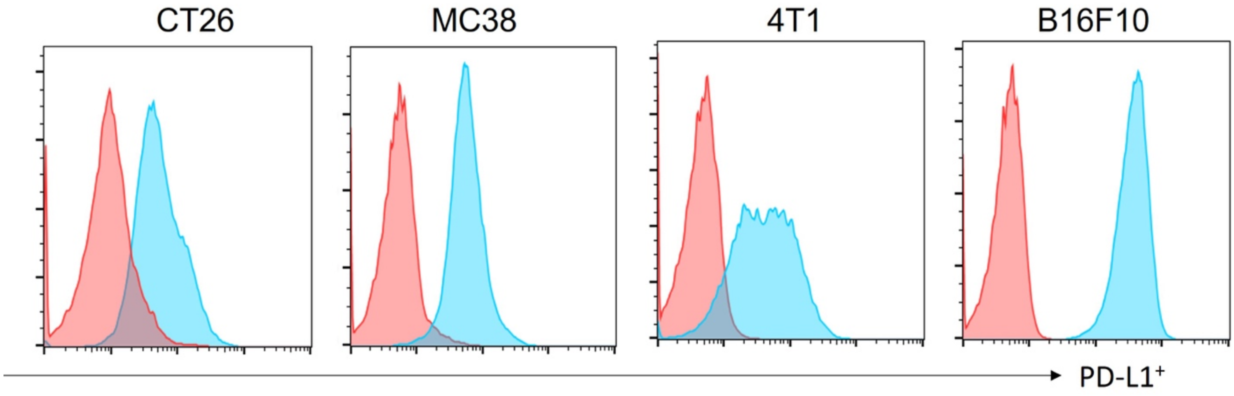


**Figure S16. PD-L1 expression on cancer cell lines (CT26, MC38, 4T1, B16F10) *in vitro*** (related to Fig. 5A). Cells were stained with IgG control antibody (red) or anti-PD-L1 antibody (green) and analyzed by flow cytometry.

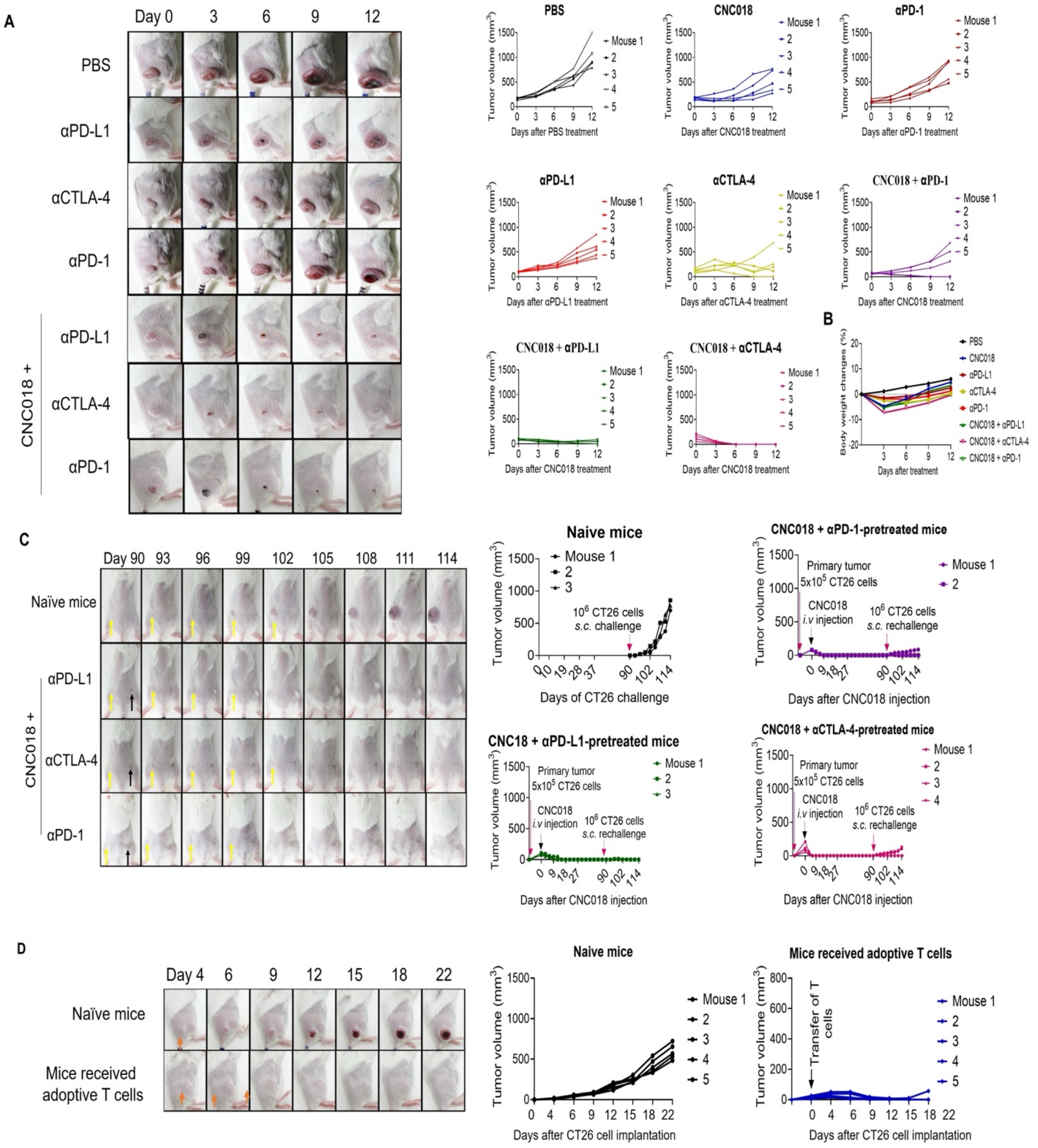


**Figure S17. CNC018 strongly potentiates the anti-tumor efficacy of ICB against CT26 tumors** (related to Figure 5I-L). CT26 cells were inoculated s.c. into the flank of BALB/c mice. Tumor-bearing mice received CNC018 ($2 \times 10^7$ CFU) via i.v. injection (day 0) and/or ICBs via i.p. injection (200 μg/mouse, twice weekly for five doses, starting on day -1). The anti-CD8 (αCD8) antibody was injected i.p. to deplete $CD8^+$ T cells. Tumor-eradicated mice were rechallenged by s.c. implantation of CT26 cells

(1 × $10^6$) into the contralateral flank on day 90; black arrows indicate the implantation sites. Naïve age-matched mice were used as controls (*n* = 3 mice). Separately, CT26 cells were implanted s.c. into the right hind flanks of mice (day 0). Adoptive transfer of T-cells (1 x $10^6$) from tumor-eradicated mice was transferred into tumor-bearing mice on day 4. (**A**) Representative images (left) and individual tumor growth curves (right) of each treated group (related to Figure 5**I**-K). (**B**) The changes of body weight after treatment. (**C**) Representative images (left) and individual growth curves of primary and rechallenged tumors (right) in naïve and tumor-eradicated mice (related to Fig. 5L). Scars of primary tumors and rechallenge sites are marked by black and yellow arrows, respectively. (**D**) Representative images (left) and individual tumor growth curves (middle and right) of mice receiving adoptive T-cells from tumor-eradicated mice (related to Fig. 5N).

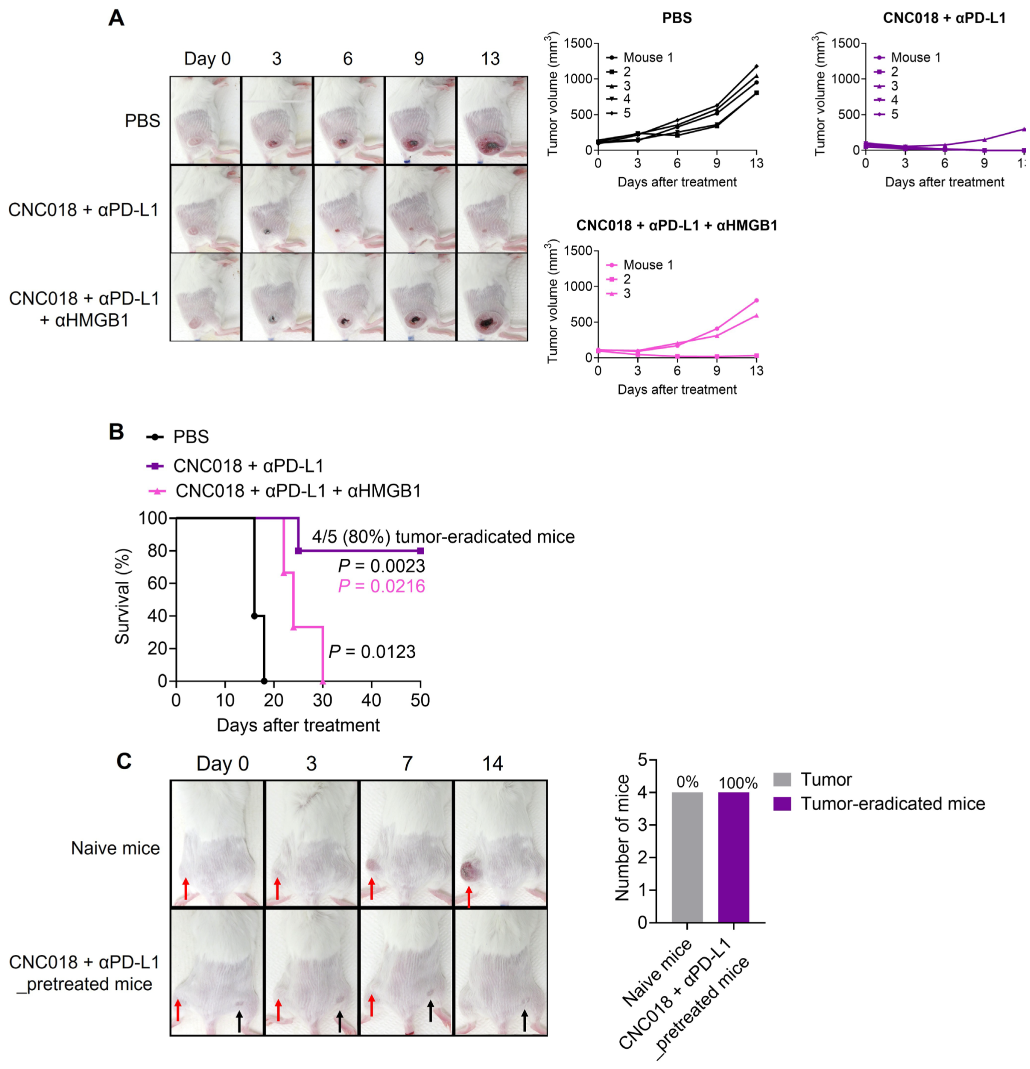


**Figure S18. HMGB1 is required for the combination of CNC018 and αPD-L1 antibody to suppress tumors** (related to Figure 5O**).** CT26 cells were inoculated *s.c.* into the right hind flank of BALB/c mice. Tumor-bearing mice received CNC018 ($2 \times 10^7$ CFU) via *i.v.* injection (day 0) and/or ICBs via *i.p.* injection (200 μg/mouse, twice weekly for five doses, starting on day -1). For HMGB1 neutralization, an αHMGB1 antibody (20 μg/mouse) was injected *i.p.* (twice weekly for three doses, starting on day -1). (**A**) Left: representative images; Right: individual tumor growth curves of each treatment group. (**B**) Kaplan-Meier survival curves of CT26 tumor-bearing BALB/c mice from (A) [*n* = 3 - 5 mice/group; log-rank (Mantel-Cox) test]. (**C**) Representative images (left) and percentages of mice without recurrence of secondary tumors after tumor rechallenge (right) in naïve and tumor-eradicated mice after treatment with CNC018 + αPD-L1 for 50 days. Scars of primary tumors and rechallenge sites are marked by black and red arrows, respectively (*n* = 4 mice/group).

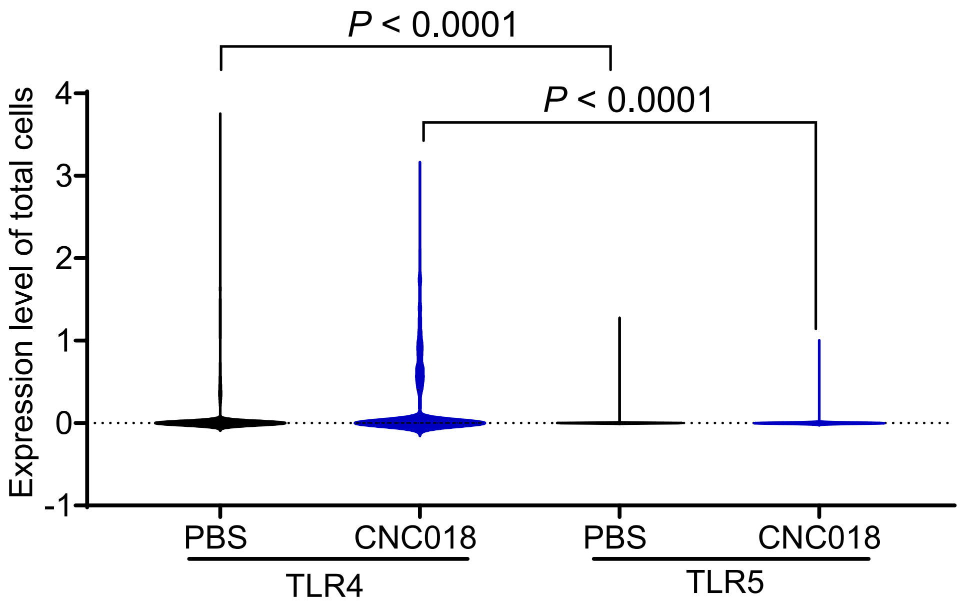


**Figure S19. ScRNA-seq analysis of TLR4 and TLR5 mRNA expression in total cells from CT26 tumors**. Mice bearing CT26 tumor were i.v. injected with PBS or CNC018 ($2 \times 10^7$ CFU) when tumor size was approximately 120 $mm^3$ (day 0). Tumors were harvested on day 3 for single-cell RNA sequencing analysis. Violin plots show the expression levels of TLR4 and TLR5 mRNA in total cells from the PBS and CNC018 groups (*n* = 2 pooled tumors/group; unpaired two-tailed *t*-test).

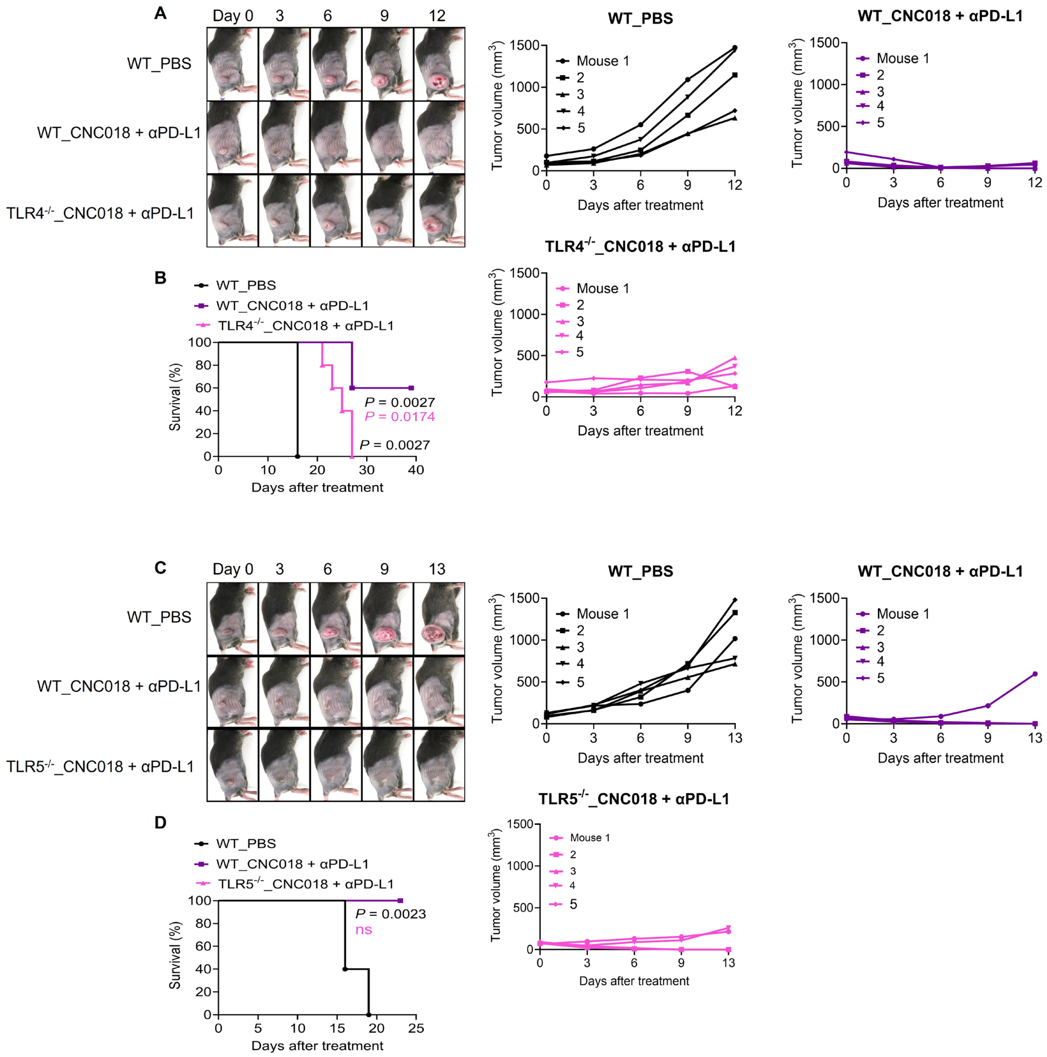


**Figure S20. Mechanistic evaluation of CNC018 and αPD-L1 combination therapy in TLR-deficient mouse models** (related to Figure 5P-Q). WT, TLR4$^{-/-}$, and TLR5$^{-/-}$ C57BL/6 mice ($n$ = 5 mice/group) were subcutaneously challenged with MC38 tumor cells. Upon reaching a mean tumor burden of ~80-100 mm³, subjects were administered either a combination of CNC018 (2 × $10^7$ CFU, i.v.) and an αPD-L1 antibody or a PBS control on day 0. (**A**) Left: representative images; Right: individual tumor growth curves (right) of each treatment group (WT_PBS, WT_CNC018 + αPD-L1, and TLR4$^{-/-}$_CNC018 + αPD-L1). (**B**) Kaplan-Meier survival curves of MC38 tumor-bearing C57BL/6 mice from (A) [$n$ = 5 mice/group; log-rank (Mantel-Cox) test]. (**C**) Left: representative images; Right: individual tumor growth curves (right) of each treatment group (WT_PBS, WT_CNC018 + αPD-L1, TLR5$^{-/-}$_CNC018 + αPD-L1). (**D**) Kaplan-Meier survival curves of MC38 tumor-bearing C57BL/6 mice from (C) [$n$ = 5 mice/group; ns, not significant; log-rank (Mantel-Cox) test].

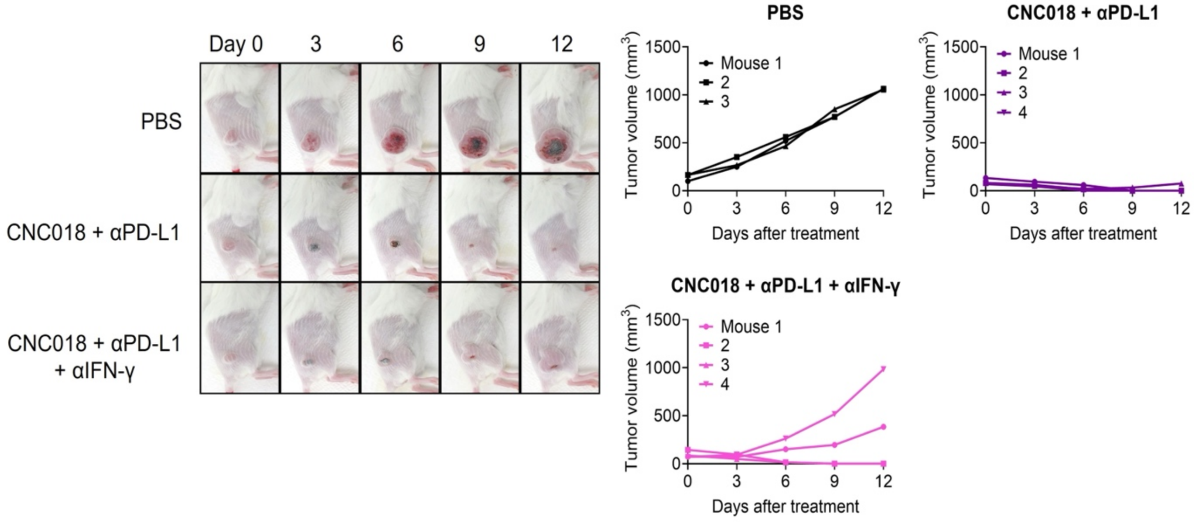


**Figure S21**. **IFN-γ is required for the combination of CNC018 and αPD-L1 antibody to suppress tumors** (related to Figure 5R)**.** BALB/c mice were subcutaneously inoculated with 5 × $10^5$ CT26 cells in the right hind flank. Once tumors were established, mice were administered CNC018 (2 × $10^7$ CFU) intravenously (day 0) and/or anti-PD-L1 antibody via intraperitoneal delivery (twice weekly for five doses, starting on day -1). For IFN-γ neutralization, an αIFN antibody (200 µg/mouse) was injected i.p. (twice weekly for five doses, starting on day -1). Left: representative images (left); Right: individual tumor growth curves of each treatment group.

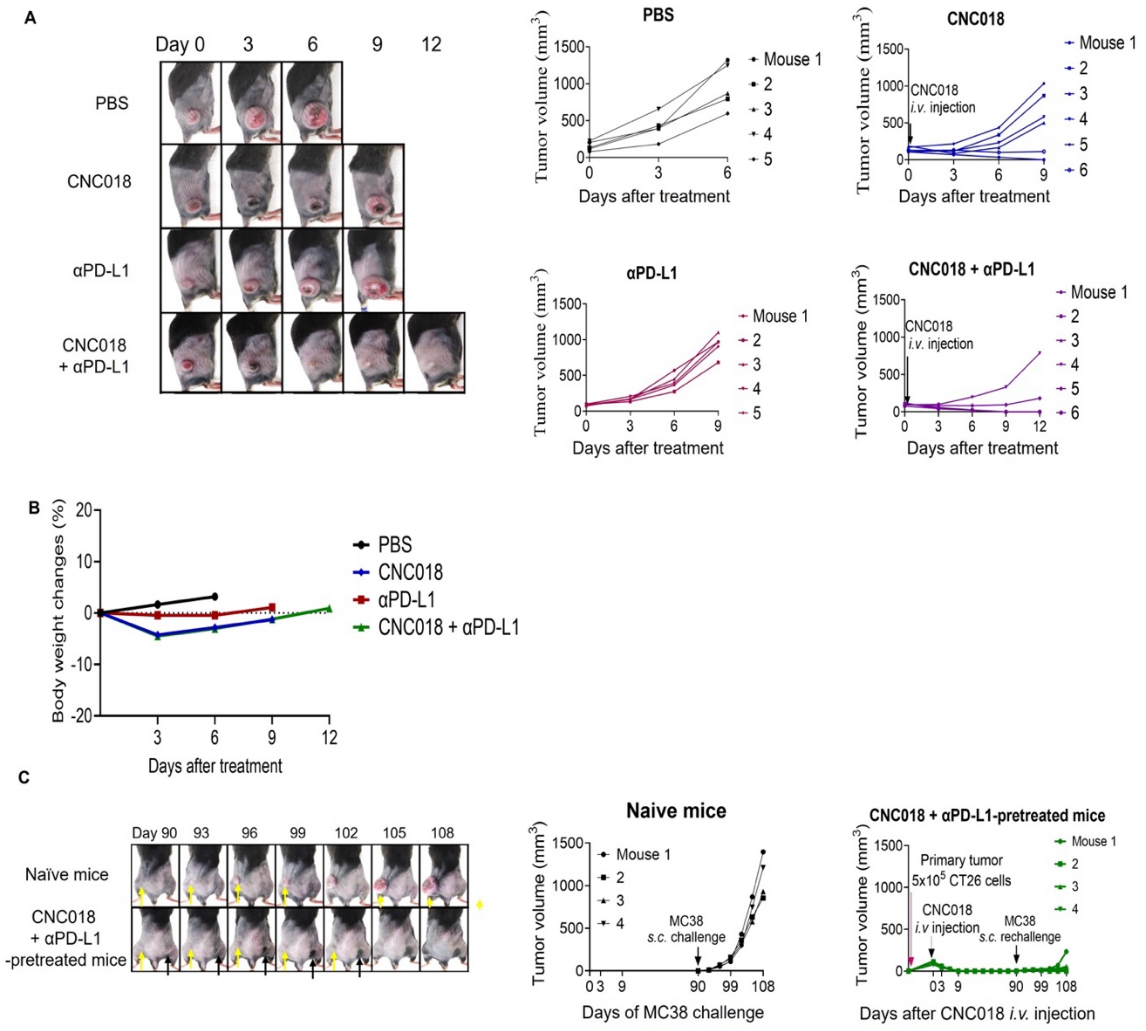

**Figure S22. Tumor suppression by the synergistic combination of CNC018 and αPD-L1 in MC38 tumor-bearing mice** (related to Figure 6A-C). C57BL/6 mice were inoculated s.c. with MC38 tumor cells ($5 \times 10^5$) on the right hind flanks. When tumors reached 100 - 120 $mm^3$ in volume, mice were injected i.v. with CNC018 ($2 \times 10^7$ CFU) (day 0). An αPD-L1 antibody was injected i.p. on day -1 (200 μg/mouse, twice weekly for five doses). For tumor rechallenge, cured mice were re-implanted s.c. with MC38 cells ($1 \times 10^6$) on the contralateral flanks on day 90. Naïve age-matched mice (*n* = 4 mice) were used as rechallenge controls. (**A**) Representative images (left) and individual tumor growth curves (right) of mice treated with PBS, CNC018, αPD-L1, or CNC018 + αPD-L1. (**B**) Body weight changes of mice after treatment. (**C**) Representative images (left) and individual tumor growth curves (right) of rechallenged tumors in naïve and cured mice. Scars of primary tumors and rechallenge sites are marked by black and yellow arrows, respectively.

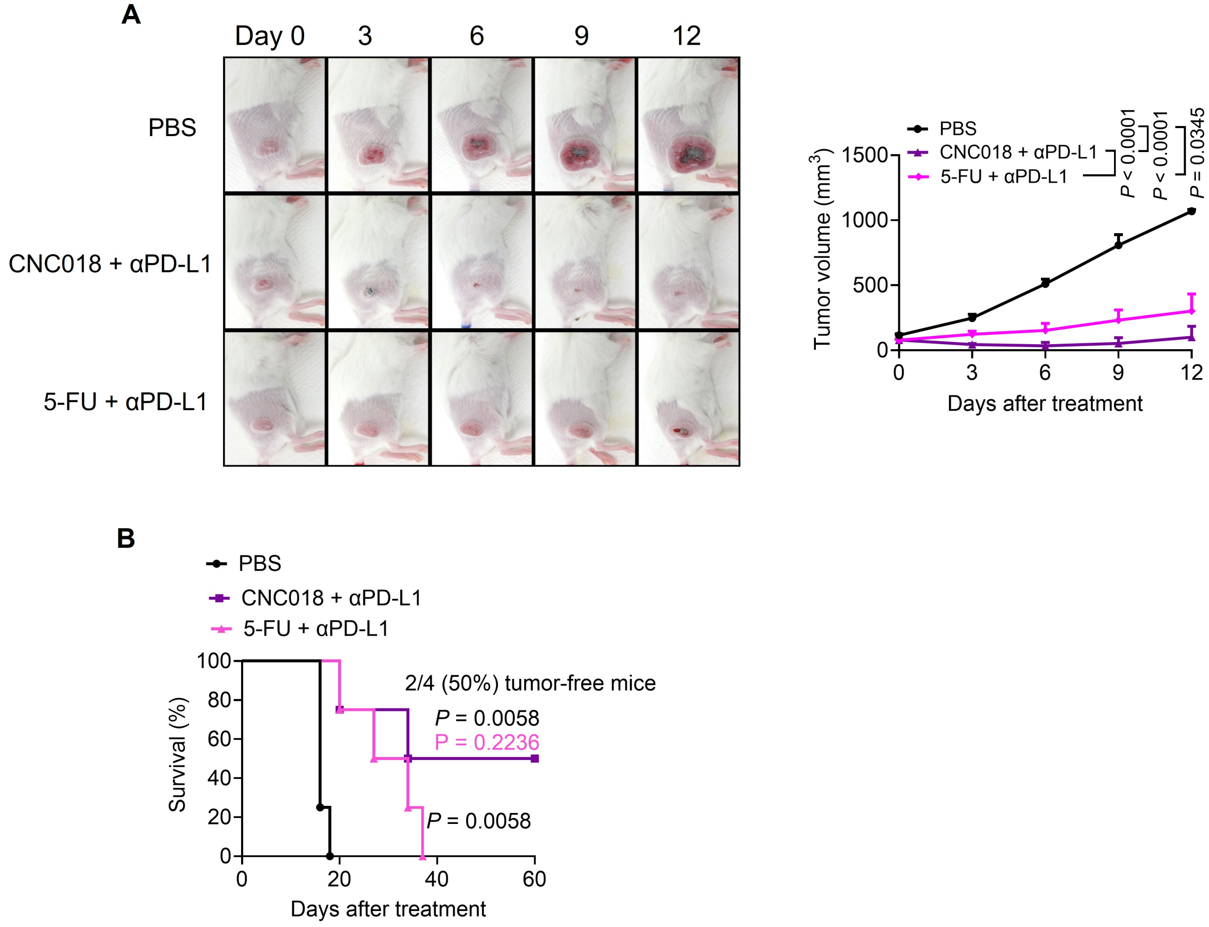


**Figure S23. Antitumor effects of 5-FU and CNC018 strain in combination with anti-PD-L1 immunotherapy.** BALB/c mice were s.c. implanted with 5 × $10^5$ of CT26 cells. When tumors reached ~100 $mm^3$ in volume, mice received CNC018 (2 × $10^7$ CFU) or 5-FU (50 mg/kg) and αPD-L1 antibody. CNC018 or 5-FU was i.v. or i.p. injected on day 0, respectively. Anti-PD-L1 (200 µg/mouse, twice weekly schedule for five doses) was i.p. injected starting on day -1. (**A**) Left: representative images of each treated group; Right: average CT26 tumor growth curves ($n$ = 4 mice/group; two-way ANOVA with Tukey's multiple comparisons test). (**B**) Kaplan-Meier survival curves of mice from (A) [$n$ = 4 mice/group; log-rank (Mantel-Cox) test].

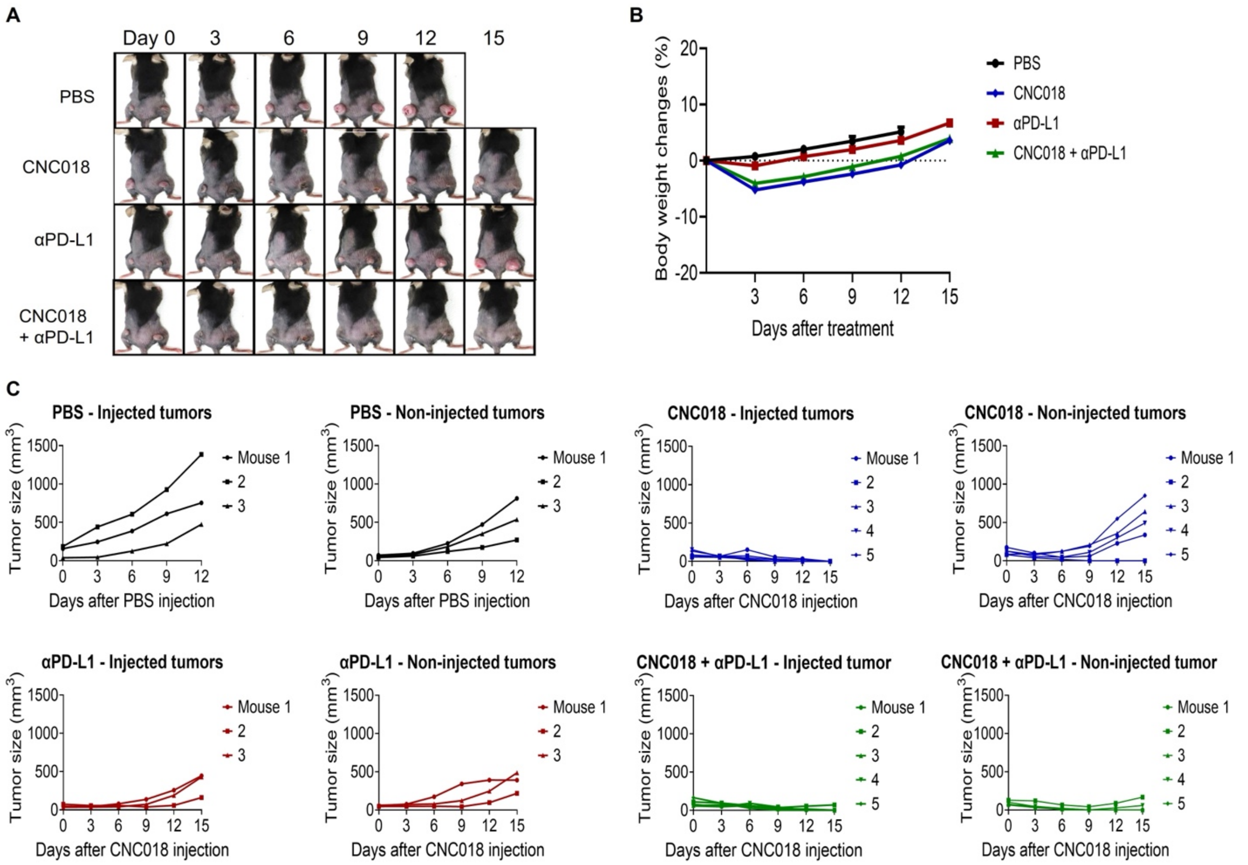


**Figure S24. Tumor suppression by the synergistic combination of CNC018 and αPD-L1 in MC38 dual tumor-bearing mice treated by intratumoral injection** (related to Figure 6N). MC38 tumor cells (5 × $10^5$) were simultaneously inoculated s.c. into both hind flanks of C57BL/6 mice. When both tumors were 130 - 150 mm³ in volume, mice were intratumorally injected with CNC018 (2 × $10^7$ CFU) into the right tumor (day 0). αPD-L1 antibody (200 µg/mouse) was injected i.p. (day -1, twice weekly for five doses). (**A**) Representative images, (**B**) body weight changes, and (**C**) individual tumor growth curves of bacteria-injected and non-injected tumors are shown.

**Table S1.** Strains and plasmids are used in this study.

| Strains or Plasmids | Descriptions | Sources/Ref. |
|---|---|---|
| Bacterial strains *S. typhimurium* | | |
| 14028S | Wild type | ATCC, USA |
| VNP20009 | *Δ$purI$, Δ$msbB$* | ATCC, USA |
| SHJ2168 | *ΔrelA, ΔspoT* (ΔppGpp) | [11] |
| CNC018 | *ΔrelA, ΔspoT, ΔSPI-1,ΔSPI-2* | This study |
| Plasmids | | |
| pKD13 | - 3.4 kb, KanR flanking FRT sequence, R6K gamma replication origin.<br>- Kanamycin resistance gene template plasmid. | Gift from Prof. Hyon E. Choy, Chonnam National University, Korea |
| pKD46 | - Ampicillin resistance.<br>- Temperature-sensitive replication origin. | |
| pCP20 | - Flipase system.<br>- Ampicillin and chloramphenicol resistance.<br>- Temperature-sensitive replication origin | |

**Table S2.** Oligonucleotide primers of PCR amplification for bacterial genotyping.

| Gene | Primer | Primer name* | Sequence | Expected size (bp) |
|---|---|---|---|---|
| *relA* | 1 | m_wtS_relA-F | AATTTCAATTTGATCACCCATCTGCAACTGA | 715 |
| | | m_wtS_relA-R | CGAACATTACATCGAAGAGTTTGTTGGAC | |
| *SpoT* | 2 | m_wtS-spoT-F | ATTGCTGAAGGTCGTCGTTAATCACA | 2185 |
| | | m_wtS-spoT-R | GCATATACGCGCATAACGTTTTGGATTC | |
| *SPI-1* | 3 | m_wtS-SPI1-spaO-F | GGGTGGAAAATGCCAGTAAGGCAATTAATGA | 990 |
| | | m_wtS-SPI1-spaO-R | AGCAACAGCACAGACAGCAAT | |
| *SPI-2* | 4 | m_wtS-SPI2-sseF-F | GGTTGATACTCTTATTGCTTAAATAACAGAACGAA | 843 |
| | | m_wtS-SPI2-sseF-R | CTGAGCATTTGGGCTAACAGGTTTCAT | |

*F, forward primer; R, reverse primer

*relA*, *spoT*: Bifunctional (p)ppGpp synthase/hydrolase (ppGpp synthesis/degradation)

*spaO*: Surface presentation of antigens protein (belongs to SPI-1 locus)

*sseF*: Secretion system effector (belongs to SPI-2 locus)

**Table S3.** Antibodies used in this study.

| Ab ID | Ab description | Manufacturer/Cat. No | Application |
|---|---|---|---|
| Anti-HMGB1 antibody – ChIP Grade | Rabbit Polyclonal Ab | Abcam/ab18256 | Western blotting |
| Goat Anti-Rabbit IgG H&L (HRP) | Polyclonal Ab | Abcam/ab205718 | |
| IL-1β Mouse ELISA Kit | Specific monoclonal Ab | Thermo Fisher Scientific / BMS6002 | Elisa |
| IFN-γ mouse ELISA Kit | Specific monoclonal Ab | Thermal Fisher/BMS606-2 | |
| TNF alpha Mouse ELISA Kit | Specific monoclonal Ab | Thermo Fisher/BMS607HS | |
| ATP Assay Kit (Colorimetric/Fluorometric) | | Abcam/ab83355 | |
| Nitric Oxide, detection kit | | Enzo/ADI-917-202 | |
| Calreticulin (D3E6) XP® Rabbit mAb (PE Conjugate) | Specific monoclonal Ab | Cell Signalling/19780S | Flow cytometry |
| Rabbit (DA1E) mAb IgG XP® Isotype Control (PE Conjugate) | Specific monoclonal Ab | Cell Signalling/5742S | |
| PE anti-mouse CD274 (B7-H1, PD-L1) Ab | Specific monoclonal Ab | Biolegend/124308 | |
| PE Mouse IgG2b, κ Isotype Ctrl Antibody | Specific monoclonal Ab | Biolegend/400312 | |

| | | |
|---|---|---|
| PE Rat IgG2a, κ Isotype Ctrl Antibody | Specific monoclonal Ab | BioLegend/400508 |
| PE Mouse IgG1, κ Isotype Ctrl Antibody | Specific monoclonal Ab | BioLegend/400112 |
| PE anti-mouse Ki-67 Antibody | Specific monoclonal Ab | BioLegend/652403 |
| Mouse IgG1 APC-conjugated Antibody | Specific monoclonal Ab | Biotechne/400312 |
| Pacific Blue anti-mouse CD45.2 antibody | Specific monoclonal Ab | BioLegend/109820 |
| Anti-mouse CD16/32 | TruStain FcX™ Fc blocking solution | BioLegend/101320 |
| Live/dead Fixable aqua dead cell stain kit | | Invitrogen/L34957 |
| Anti-mouse CD3-FITC | Clone 17A2 | eBioscience/11-0032-82 |
| Anti-mouse CD11c-FITC | Clone N418 | eBioscience/11-0114-82 |
| Anti-mouse 11b-FITC | Clone M1/70 | BioLegend/101205 |
| Anti-mouse CD4-APC | Clone GK1.5 | BioLegend/100412 |
| Anti-mouse CD8a-PE | Clone 53-6.7 | eBioscience/12-0081-82 |
| Anti-mouse CD274-PE | Clone 10F.9G2 | BioLegend/12-0081-82 |

| | | | |
|---|---|---|---|
| Anti-mouse F4/80-PE | Clone BM8 | eBioscience/124801-82 | |
| Anti-mouse IL-1β PE | Clone CRM56 | eBioscience/12-7018-82 | |
| Anti-mouse CD86-PE | Clone GL1 | eBioscience/12-0862-82 | |
| Anti-mouse CD49b-PE | Clone DX5 | BioLegend/108908 | |
| FOXP3 Monoclonal Antibody (FJK-16s)-PE | Clone FJK-16S | Ebioscience/12577383 | |
| Anti-mouse FOXP3-APC | Clone FJK-16s | eBioscience/77-5775-40 | |
| Anti-mouse CD49b-APC | Clone DX5 | BioLegend/108909 | |
| Anti-mouse CD4-APC | Clone GK1.5 | BioLegend/100412 | |
| Anti-mouse Gr-1-APC | Clone 1A8 | BioLegend/127614 | |
| Anti-mouse CD274-APC | Clone B7-H1 | BioLegend/124312 | |
| Anti-mouse CD206-APC | Clone B7-H1 | BioLegend/C068C2 | |
| Anti-mouse CD25-PerCP-Cy5.5 | Clone PC61 | BioLegend/102030 | |
| Anti-mouse CD279-PerCP-Cy5.5 | Clone RMP130 | BioLegend/109120 | |
| Anti-mouse granzyme B-PerCP-Cy5.5 | Clone QA16A02 | BioLegend/372212 | |
| Anti-mouse CD8-PerCP-Cy5.5 | Clone 53-6.7 | BioLegend/100733 | |
| Anti-mouse CD62L-PerCP-Cy5.5 | Clone MEL-14 | BioLegend/104432 | |

| | | |
|---|---|---|
| FITC anti-mouse/human CD44 Antibody | Clone IM7 | BioLegend/103006 |
| Anti-mouse MHCII-PerCP-Cy5.5 | Clone M5/114.15.2 | BioLegend/107625 |
| Anti-mouse Ly-6G-PerCP-Cy5.5 | Clone RB6-8C5 | BioLegend/127615 |
| Anti-mouse F4/80-PerCP-Cy5.5 | Clone BM8 | BioLegend/123128 |
| Anti-mouse Ki-67-PE-Cy7 | Clone SolA15 | Thermo Fisher Scientific/25469882 |
| PE/Cy7 anti-mouse CD152 | Clone UC10-4B9 | BioLegend/106313 |
| Anti-mouse CD8 PE-Cy7 | Clone 53-6.7 | eBioscience/25-0081-82 |
| Anti-mouse CD206 PE-Cy7 | Clone C068C2 | BioLegend/141719 |
| Anti-mouse CD11c PE-Cy7 | Clone N418 | Thermo Fisher Scientific/25-0114-82 |
| Anti-mouse CTLA-4 PE-Cy7 | Clone UC10-4B9 | Biolegend/106313 |
| Anti-mouse PD-L1 PE-Cy7 | Clone B7-H1 | EbioScience/25598342 |
| Anti-mouse IFNγ-APC-Cy7 | Clone XMG1.2 | BioLegend/505849 |
| Anti-mouse PD-1-APC-Cy7 | Clone 29F.1A12 | BioLegend/135224 |
| Anti-mouse CD44-APC-Cy7 | Clone IM7 | BioLegend/103028 |
| Anti-mouse CD86-APC-Cy7 | Clone GL-1 | BioLegend/105029 |
| PE anti-mouse TCR β chain Antibody | Clone H57-597 | BioLegend/109208 |

| | | | |
|---|---|---|---|
| PE anti-mouse CD170 (Siglec-F) Antibody | Clone S17007L | Biolegend/155506 | |
| APC/Cyanine 7 anti-mouse CD16 Antibody | Clone S17014E | Biolegend/158014 | |
| PE/Cyanine7 anti-mouse CD54 Antibody | Clone YN1.1.7.4 | Biolegend/116122 | |
| H-2Ld MuLV gp70 Tetramer-SPSYVYHQF-APC | Clone 53-6.7 | MBL/TM-M521-1 | |
| Anti-HMGB1, ChIP grade | Polyclonal Ab | Abcam | HMGB1 Western blotting |
| rabbit anti-CALR antibody | Polyclonal Ab | Abcam | CALR Western blotting |
| InVivoMAb anti-mouse IFNγ | XMG1.2 | Bioxcell/BE0055 | IFN-γ neutralizing |
| HMGB1 Neutralizing antibody [SQab20175] | Polyclonal Ab | Arigo/ARG66714 | HMGB1 neutralization |
| InVivoMAb anti-mouse PD-L1 (B7-H1) | Clone 10F.9G2 | Bio X Cell/BE0101 | PD-L1 neutralization |

| | | | |
|---|---|---|---|
| *InVivo*MAb rat IgG2b isotype control | Clone LTF-2 | Bio X Cell/BE0090 | PD-L1 neutralization control |
| *InVivo*MAb anti-mouse PD-1 (CD279) | Clone RMP1-14 | Bio X Cell/BE0146 | PD-1 neutralization |
| *InVivo*MAb rat IgG2a isotype control, anti-trinitrophenol | Clone 2A3 | Bio X Cell/BE0089 | PD-1 neutralization control |
| *InVivo*MAb anti-mouse CTLA-4 (CD152) | Clone 9H10 | Bio X Cell/BE0131 | CTLA-4 neutralization |
| *InVivo*MAb polyclonal Syrian hamster IgG | Clone polyclonal | Bio X Cell/BE0087 | CTLA-4 neutralization control |
| Anti-mouse CD8α | Clone 2.43 | Bio X Cell/BP0061 | $CD8^+$ T depletion |
| Rat IgG2b isotype control Ab | Clone LTF-1 | Bio X Cell/BP0090 | T-cell depletion control |
| CD3ε MicroBead Kit, mouse | | Miltenyi Biotec/130-094-973 | Harvesting $CD3^+$ T cells |
| LS columns | | Miltenyi Biotec/130-042-401 | Harvesting $CD3^+$ T cells |

**Table S4.** Xenograft pathological subtype changes of patient colon cancer.

| Patient ID | Age | Gender | Histocompatibility | | | | | | |
|---|---|---|---|---|---|---|---|---|---|
| | | | Cancer | Stroma | Tumor necrosis | Extracellular mucus in tumors | Inflammatory response around the tumor | Intratumor interstitial fibrosis | Control normal tissue in the slide |
| NM-P-C-6 | 61 | Female | 70% | 30% | Yes, 5% | Yes, 10% | Intermediate | 60% | No |

**Table S5.** CDI value of CNC018 plus ICB blockade in CT26 and MC38 tumor models.

| Average tumor volume ($mm^3$) | Control (PBS) | A (CNC018) | B (αPD-L1) | AB (CNC018 + αPD-L1) | CDI | Interpretation |
|---|---|---|---|---|---|---|
| CT26 tumor volume (day 12) | 1033.4 | 515.8 | 564.8 | 37.74 | 0.13 | Significant synergism |
| MC38 tumor volume (day 9) | 1629 | 925.6 | 597.8 | 85.2 | 0.25 | Significant synergism |
| Average tumor volume ($mm^3$) | Control (PBS) | A (CNC018) | B (αCTLA-4) | AB (CNC018 + αCTLA-4) | CDI | Interpretation |
| CT26 tumor volume (day 12) | 1033.4 | 515.8 | 252.1 | 0 | 0 | Significant synergism |
| Average tumor volume ($mm^3$) | Control (PBS) | A (CNC018) | B (αPD-1) | AB (CNC018 + αPD-1) | CDI | Interpretation |
| CT26 tumor volume (day 12) | 1033.4 | 515.8 | 758.7 | 301.8 | 0.80 | Synergism |

CDI, coefficient of drug interaction; CDI = (AB/control)/[(A/control) x (B/control)].
